# Genetic predisposition to metabolically unfavourable adiposity and prostate cancer risk: A Mendelian randomization analysis

**DOI:** 10.1002/cam4.6220

**Published:** 2023-06-12

**Authors:** Aurora Perez‐Cornago, Karl Smith‐Byrne, Emma Hazelwood, Cody Z. Watling, Susan Martin, Timothy Frayling, Sarah Lewis, Richard M. Martin, Hanieh Yaghootkar, Ruth C. Travis, Timothy J. Key

**Affiliations:** ^1^ Cancer Epidemiology Unit, Nuffield Department of Population Health University of Oxford Oxford UK; ^2^ MRC Integrative Epidemiology Unit University of Bristol Bristol UK; ^3^ Population Health Sciences, Bristol Medical School University of Bristol Bristol UK; ^4^ Institute of Biomedical and Clinical Science, University of Exeter Medical School, Research, Innovation, Learning and Development building, Royal Devon & Exeter Hospital Exeter UK; ^5^ NIHR Bristol Biomedical Research Centre University Hospitals Bristol and Weston NHS Foundation Trust and the University of Bristol Bristol UK; ^6^ Centre for Inflammation Research and Translational Medicine (CIRTM), Department of Life Sciences Brunel University London Uxbridge UK; ^7^ Research Centre for Optimal Health, School of Life Sciences University of Westminster London UK

**Keywords:** adiposity, advanced disease, Mendelian randomization, prostate cancer

## Abstract

**Background:**

The associations of adiposity with aggressive prostate cancer risk are unclear. Using two‐sample Mendelian randomization, we assessed the association of metabolically unfavourable adiposity (UFA), favourable adiposity (FA) and for comparison body mass index (BMI), with prostate cancer, including aggressive prostate cancer.

**Methods:**

We examined the association of these genetically predicted adiposity‐related traits with risk of prostate cancer overall, aggressive and early onset disease using outcome summary statistics from the PRACTICAL consortium (including 15,167 aggressive cases).

**Results:**

In inverse‐variance weighted models, there was little evidence that genetically predicted one standard deviation higher UFA, FA and BMI were associated with aggressive prostate cancer [OR: 0.85 (95% CI:0.61–1.19), 0.80 (0.53–1.23) and 0.97 (0.88–1.08), respectively]; these associations were largely consistent in sensitivity analyses accounting for horizontal pleiotropy. There was no strong evidence that genetically determined UFA, FA or BMI were associated with overall prostate cancer or early age of onset prostate cancer.

**Conclusions:**

We did not find differences in the associations of UFA and FA with prostate cancer risk, which suggest that adiposity is unlikely to influence prostate cancer via the metabolic factors assessed; however, these did not cover some aspects related to metabolic health that may link obesity with aggressive prostate cancer, which should be explored in future studies.

## 1 BACKGROUND

In men, prostate cancer is the most commonly diagnosed cancer and is the second leading cause of cancer death worldwide.^1^ The only potentially modifiable risk factors identified to date are insulin‐like growth factor‐I (IGF‐I) and free testosterone.^2^, ^3^ Some evidence suggests that adiposity may be related to prostate cancer risk, but this association appears to vary by tumour subtypes; an inverse association has been observed between obesity and risk of overall prostate cancer and non‐aggressive prostate cancer (slow‐growing tumours), while obesity has been positively associated with risk for aggressive (fast‐growing with lethal progression) prostate cancer, including dying from prostate cancer.^4^, ^5^ However, it is unclear whether the association with aggressive forms of the disease is due to late detection (which may lead to poorer prognosis), is biologically driven, or is a combination of both.

Previous prospective studies investigating the association between adiposity and prostate cancer risk have mainly used body mass index (BMI), which does not distinguish between fat and muscle mass, as a surrogate of adiposity instead of a more accurate measure. Moreover, there is some evidence suggesting that while some individuals within the normal range of BMI may have excessive adiposity, some individuals within the overweight/obese range of BMI do not seem to have metabolic disturbances.^6^ However, it is unknown if these metabolic disturbances may be the mechanisms linking obesity to prostate cancer. A recent genome‐wide association study (GWAS) has identified two clusters of genetic variants associated with higher adiposity: one associated with an ‘unfavourable’ metabolic profile (unfavourable adiposity, UFA) and another with a ‘favourable’ metabolic profile (favourable adiposity, FA), using body fat percentage and metabolic biomarkers [i.e. HDL cholesterol (HDL‐C), sex hormone–binding globulin (SHBG), triglycerides (TG), aspartate transaminase (AST) and alanine aminotransferase (ALT)]^6^ to define the clusters. The UFA were associated with lower HDL‐cholesterol and SHBG, and higher triglycerides and liver enzymes, and vice‐versa for FA. In this GWAS, the adiposity‐increasing alleles in both UFA and FA were associated with higher subcutaneous adipose tissue (SAT), BMI and C‐reactive protein (CRP). The UFA alleles were associated with higher deposition of all fat depots including visceral fat and ectopic fat (i.e. liver and pancreas), and were associated with higher risk of cardio‐metabolic disease, while the FA alleles were associated with lower liver fat and a lower risk of cardiometabolic diseases (e.g. Type 2 diabetes, heart disease, stroke).^6^

Mendelian randomization (MR) analyses, which use genetic variants as proxies for exposures,^7^ may help to address reverse causation and confounding in observational studies, and therefore help to clarify the association between adiposity and prostate cancer. In this study, we sought to estimate the effects of UFA and FA on prostate cancer risk using a two‐sample MR framework, updating previously published results on total prostate cancer risk based on 79,194 cases and 61,112 controls,^8^ and for the first time describing associations with aggressive and early‐onset disease. For this, we used genetic instruments identified from UK Biobank and genetic data from the PRACTICAL consortium (up to 85,554 prostate cancer cases [15,167 aggressive and 6988 early‐onset subtypes] and 91,972 controls).^9^, ^10^ For comparison of these new adiposity measurements with classic measurements of adiposity, our secondary aim was to investigate the association of BMI‐related genetic variants with prostate cancer risk.

## 2 METHODS

### 2.1 Selection of instrumental variables

Single nucleotide polymorphisms (SNPs) predicting UFA and FA were taken from a large genome‐wide association study (GWAS) based on up to 429,203 men and women of European ancestry from the UK Biobank.^6^ For BMI, we used genome‐wide significant and independent SNPs from a large genome‐wide association study (GWAS) in the Genetic Investigation of ANthropometric Traits (GIANT) and UK Biobank in 694,649 men and women of European ancestry.^11^ SNPs were variants associated with these adiposity traits at the *p* < 5 × 10^−8^ significance level and had linkage disequilibrium (LD) threshold of *r* ^2^ < 0.01.

No evidence of between‐sex heterogeneity was observed for SNP associations with the three genetic risk scores above,^6^, ^11^ therefore summary statistics from the GWASs on men and women combined were used. All SNPs left after clumping were considered as the instrumental variables and included a total of up to 27 UFA, 34 FA, and 506 BMI variants (Tables S1–S3).

### 2.2 Genetic associations with prostate cancer

We obtained GWAS summary statistics from the PRACTICAL (including GAME‐ON/ELLIPSE) consortia^9^, ^12^ for aggressive prostate cancer cases (also referred to as ‘advanced’ by the PRACTICAL Consortium), which was defined as metastatic disease, a Gleason score ≥8, a prostate‐specific antigen (PSA) >100 ng/mL, or death due to prostate cancer (15,167 cases, 58,308 controls) as well as summary GWAS statistics for early age of onset prostate cancer (age at diagnosis ≤55 years; 6988 cases, 44,256 controls). GWAS summary statistics for prostate cancer overall (85,554 prostate cancer cases and 91,972 controls) were accessed in the database of Genotypes and Phenotypes (dbGaP) (application Project # 31553).^10^ All participants were of European ancestry.

### 2.3 MR analyses

We used a two‐sample MR approach to estimate the associations of UFA, FA and BMI with overall, aggressive and early‐onset prostate cancer risk. Data were harmonised to ensure that the exposure dataset (adiposity measures) had the same effect allele as the outcome dataset (overall, aggressive and early‐onset prostate cancer). If a SNP was found to not have the same effect allele, the SNP would be oriented to the effect allele. Palindromic SNPs were excluded if the effect allele frequency was found to be >0.42. ~10% of all SNPs did not harmonise from the exposure dataset for some of the outcomes of interest, due to them being unavailable in the outcome dataset, and in these specific instances the SNPs were excluded for the respective analyses. The main MR estimation method was the multiplicative random effects inverse‐variance weighted (IVW) method.^13^ We additionally calculated the *I* ^2^ statistic to assess the potential violations of the no‐measurement error (NOME) assumption in SNP‐exposure associations and the Cochran's *Q* statistic for heterogeneity between the MR estimates for each SNP. Moreover, we used PhenoScanner to assess whether the selected genetic instruments were associated with secondary phenotypes (Figures S1–S3). All MR analyses were repeated for each outcome (i.e. overall prostate cancer, aggressive prostate cancer, early‐onset prostate cancer) and estimates from MR are presented as odds ratios per one standard deviation (1‐SD) higher UFA, FA or BMI.

#### 2.3.1 Sensitivity analyses

We additionally estimated the associations of adiposity traits with prostate cancer using methods that are more robust to horizontal pleiotropy. For this, we used the weighted median method, the MR residual sum and outlier (MR‐PRESSO) method, and the contamination mixture method. The weighted median model provides an unbiased estimate when genetic variants without horizontal pleiotropic effects contribute at least 50% of the information in an instrument. The MR residual sum and outlier (MR‐PRESSO) method^14^ can detect and adjust for horizontal pleiotropy by removing outliers, while the contamination mixture method performs MR analyses more robustly and efficiently when there are invalid instrumental variables.^15^ We also estimated the MR‐Egger intercept,^16^ and reported risk associations using the MR‐Egger method if there was evidence of directional pleiotropy. Finally, ‘leave‐one‐out’ sensitivity analyses were performed using the TwoSampleMR package in R.^17^

#### 2.3.2 Software

We used the ‘TwoSampleMR’ R package to undertake all MR analyses^17^ and figures were plotted in R version 4.0.5. All tests of significance were two‐sided.

## 3 RESULTS

There was limited evidence of weak instrument bias (*F*‐statistic ranged from 71.7 to 149.4), and the proportion of variance in the phenotypes (*R* ^2^) explained by the genetic instruments was 1.44% for UFA, 0.64% for FA and 5.8% for BMI. There was no strong evidence of violation of the NOME assumption for the UFA, FA and BMI genetic instruments (*I* ^2^ statistic >0.90).

**FIGURE 1:**
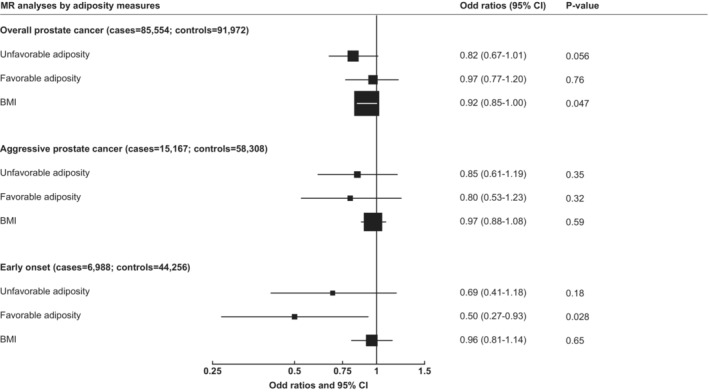
Mendelian randomization estimates of the associations of genetically predicted unfavourable and favourable adiposity and BMI with overall, aggressive and early‐onset prostate cancer using the inverse weighted variance method. Estimates are presented as odds ratios per one standard deviation increase of each adiposity measurement. Aggressive disease was defined as Gleason Score 8+, death from prostate cancer, metastatic disease, or PSA > 100 ng/mL. Early‐onset defined as diagnosed aged ≤55 years.

There was little evidence that genetically determined 1‐SD higher UFA was associated with risks of overall, aggressive, or early onset prostate cancer in the IVW MR analyses [OR: 0.82 (0.67–1.01), 0.85 (0.61–1.19) and 0.69 (0.41–1.18), respectively] (Figure 1). Methods robust to violations of the MR assumptions were largely consistent with the IVW estimates, although some evidence of an inverse association between UFA and overall prostate cancer was observed when the weighted median [0.73 (0.57–0.94)] method was used (Table 1).

**TABLE 1:** Mendelian randomization estimates of the associations of genetically predicted unfavourable and favourable adiposity and BMI with overall, advanced and early‐onset prostate cancer.

	Overall prostate cancer (85,554 cases, 91,972 controls)			Aggressive prostate cancer^a^ (15,167 cases, 58,308 controls)			Early onset^b^ (6988 cases, 44,256 controls)		
	N SNPs	OR (95% CI) per 1 SD increase	*p*‐Value	N SNPs	OR (95% CI) per 1 SD increase	*p*‐Value	N SNPs	OR (95% CI) per 1 SD increase	*p*‐Value
Unfavourable adiposity	27			22			22		
Inverse weighted variance		0.82 (0.67–1.01)	0.056		0.85 (0.61–1.19)	0.350		0.69 (0.41–1.18)	0.177
Weighted median		0.73 (0.57–0.94)	0.014		0.83 (0.54–1.29)	0.411		0.67 (0.34–1.30)	0.236
MR‐Egger intercept			0.974			0.974			0.070
Contamination mixture		0.59 (0.45–1.02)	0.066		0.75 (0.43–1.32)	0.203		0.71 (0.36–1.30)	0.203
Favourable adiposity	34			27			27		
Inverse weighted variance		0.97 (0.77–1.20)	0.759		0.80 (0.53–1.23)	0.316		0.50 (0.27–0.93)	0.028
Weighted median		0.80 (0.58–1.09)	0.149		0.77 (0.43–1.38)	0.386		0.60 (0.24–1.45)	0.255
MR‐Egger intercept			0.526			0.826			0.729
Contamination mixture		1.02 (0.53–1.70)	0.852		0.71 (0.40–1.29)	0.217		0.88 (0.28–1.87)	0.708
BMI	506			450			446		
Inverse weighted variance		0.92 (0.85–1.00)	0.047		0.97 (0.88–1.08)	0.589		0.96 (0.81–1.14)	0.653
Weighted median		0.87 (0.79–0.96)	0.004		0.94 (0.78–1.13)	0.499		1.04 (0.79–1.36)	0.783
MR‐Egger intercept			0.722			0.977			0.553
MR‐PRESSO		0.91 (0.86–0.97)^c^	0.003		0.96 (0.87–1.06)^c^	0.391		0.95 (0.83–1.10)^c^	0.411
Contamination mixture		0.90 (0.85–1.01)	0.090		1.13 (0.98–1.28)	0.105		1.03 (0.83–1.24)	0.818

Abbreviations: BMI, body mass index; CI, confidence interval; MR, Mendelian randomization; OR, odds ratio; PRESSO, pleiotropy residual sum and outlier; PSA, prostate‐specific antigen; SD, standard deviation.

^a^ Aggressive disease was defined as Gleason Score 8+, death from prostate cancer, metastatic disease, or PSA > 100 ng/mL.

^b^ Early‐onset defined as diagnosed aged ≤55 years.

^c^ For MR‐PRESSO, only outlier corrected results are presented.

For genetically determined FA, there was little evidence of associations with overall or aggressive prostate cancer [0.97 (0.77–1.20) and 0.80 (0.53–1.23), respectively] (Figure 1), and similar estimates were found in the sensitivity analyses conducted to provide robust estimates in the presence of pleiotropy. An inverse association between FA and early onset prostate cancer was observed in IVW model [0.50 (0.27–0.93)], although there was little evidence to support this association in MR sensitivity analyses **(**Table 1).

In analyses looking at the association of genetically predicted BMI with prostate cancer risk, little evidence of associations with aggressive and early onset prostate cancer was observed. The odds ratio for BMI in relation to overall prostate cancer from the IVW MR analysis was 0.92 (0.85–1.00) per SD, and results were similar in the sensitivity analyses (Table 1).

There was significant heterogeneity in the MR estimates for UFA SNPs with overall prostate cancer and also for BMI SNPs with overall, aggressive and early onset prostate cancer (Cochran's *Q p* < 0.05), while no evidence of heterogeneity was observed from the MR analyses for the rest of exposure SNPs and outcomes. Moreover, scatter plots comparing different MR models (Figures S4–S6) and results of the ‘leave‐one‐out’ analyses (Figures S7–S15) show that our results do not seem to be swayed by one very influential SNP.

## 4 DISCUSSION

In MR analyses using published instruments for UFA and FA we sought to separately examine the roles of higher adiposity with and without its adverse metabolic effects on risk for prostate cancer and, in particular, aggressive prostate cancer. The results do not support a strong association of either the adverse metabolic component of obesity or the non‐metabolic component with prostate cancer risk. Moreover, we did not find differences in the associations of UFA and FA with prostate cancer risk, which suggests that adiposity is unlikely to influence prostate cancer via the metabolic factors assessed.

A recent MR study looking at the associations of UFA and FA genetic variants with 37 chronic diseases, including multiple cancer sites, found that for conditions where the metabolic effect of higher adiposity is likely the primary cause of the disease (e.g. coronary artery disease, hypertension, Type 2 diabetes), the FA genetic variants showed inverse associations with disease risk, while UFA genetic variants showed positive associations with risk of these diseases.^8^ However, for diseases where the non‐metabolic effects of excess body weight (e.g. mechanical effect) are likely to cause the disease, both FA and UFA genetic variants showed a positive association with risk (e.g. osteoarthritis, gastro‐oesophageal reflux disease).^8^ This previous study^8^ also looked at the associations of UFA, FA and BMI genetic variants with risk of overall prostate cancer, with inconclusive findings; however, this study included a GWAS of overall prostate cancer with a smaller number of overall prostate cancer cases and controls than in the current analysis, and did not look at aggressive and early‐onset disease.

The possible inverse association of BMI with overall prostate cancer found in our MR analyses is consistent with evidence from a meta‐analysis of individual participant prospective observational data.^18^ Understanding the reasons why excessive adiposity is associated with prostate cancer is important to be able to advise health professionals and individuals about the possible health risks associated with obesity. A previous large cross‐sectional study found that men with higher BMI also have lower free testosterone concentrations,^19^ and free testosterone has been positively associated with prostate cancer risk in observational and Mendelian randomization studies,^2^ which might explain this association. However, it is also likely that differences in prostate cancer detection play a role in the possible inverse association of BMI with risk of overall prostate cancer. It has been hypothesised that men with obesity may have a delayed prostate cancer diagnosis compared to men with normal weight due to several reasons: (1) a previous meta‐analysis showed that men with obesity have a 12.9% lower prostate‐specific antigen concentration compared to men with a normal weight^20^; (2) greater difficulty for healthcare professionals to complete a comprehensive digital rectal examination in men with obesity, and thus lower likelihood of undergoing a biopsy^20^, ^21^, ^22^; (3) large prostate size may make detecting cancer via biopsy more difficult due to needles missing the cancer.^20^, ^21^, ^22^ This possible delayed prostate cancer diagnosis would be expected to lead to worse prognosis, which would support findings from previous observational studies on obesity and aggressive prostate cancer.^4^, ^5^ Moreover, we found suggestive evidence that FA had an effect on early‐onset prostate cancer risk, which would support the hypothesis that prostate cancer may be detected sooner in men with lower adiposity; however, sensitivity analyses did not support this association. Prospective studies with adiposity measurements and tumour characteristic data at prostate cancer diagnosis, together with studies with data on PSA testing during the follow‐up period, are needed to clarify this association.

Our study has several limitations that should be considered. While MR can provide valid estimates when assumptions are satisfied, UFA and FA are complex traits derived from a statistical decomposition of genetic determinants of a large number of metabolic exposures and so may be vulnerable to horizontal pleiotropy. However, we did not find evidence for directional pleiotropy, though modest heterogeneity in the SNP association for UFA and BMI was observed for prostate cancer overall. We also note that, while *F* statistics were above the conventional cut‐off of 10, there was relatively low variance explained by the UFA and FA SNPs, which would lead to low precision in some analyses and insufficient power to detect an effect of UFA and FA on prostate cancer, particularly in analyses assessing associations with aggressive and early onset disease. Additionally, our analyses did not control for the influence of PSA on prostate cancer diagnosis and the association of UFA and FA with PSA is unknown. Our results may therefore also have been limited by detection bias and its influence on case ascertainment in cancer GWAS. The genetic variants used to define UFA and FA are both associated with higher total adiposity and BMI, subcutaneous adipose tissue and CRP, and so there were some genetic variants present in both genetic risk scores. The UFA and FA genetic instruments only captured certain aspects of metabolic health (i.e. HDL‐C, SHBG, TG, AST and ALT) and did not include genetic variants related to low‐grade inflammation profile, the insulin pathway, and glycaemic control,^6^ which are related to metabolic health and have been proposed to increase prostate cancer risk in previous studies.^23^, ^24^ Finally, our analyses included only individuals of European descent therefore these findings may not be generalizable to other populations and further research is needed to assess this.

In summary, our findings from Mendelian randomization analyses do not support a strong effect of specific adiposity and metabolic profiles using genetic instruments identified in a large prospective cohort on risk for aggressive prostate cancer: as for BMI, there was no clear evidence that either the adverse metabolic component of obesity or the non‐metabolic component of obesity was associated with risk for aggressive prostate cancer, overall prostate cancer or early onset disease. Moreover, we did not find differences in the associations of UFA and FA with prostate cancer risk, which suggests that adiposity is unlikely to influence prostate cancer via the metabolic factors assessed. However, the UFA and FA genetic instruments did not cover some aspects related to metabolic health that may link obesity with aggressive prostate cancer and it was not possible to account for detection differences, which should be explored in future studies.

## AUTHOR CONTRIBUTIONS

**Aurora Perez‐Cornago:** Conceptualization (lead); formal analysis (lead); funding acquisition (lead); investigation (lead); methodology (lead); project administration (lead); visualization (lead); writing – original draft (lead); writing – review and editing (lead). **Karl Smith‐Byrne:** Data curation (supporting); methodology (supporting); writing – review and editing (supporting). **Emma Hazelwood:** Investigation (supporting); writing – review and editing (supporting). **Cody Z. Watling:** Visualization (supporting); writing – review and editing (supporting). **Susan Martin:** Writing – review and editing (supporting). **Timothy Frayling:** Writing – review and editing (supporting). **Sarah Lewis:** Writing – review and editing (supporting). **Richard M. Martin:** Writing – review and editing (supporting). **Hanieh Yaghootkar:** Writing – review and editing (supporting). **Ruth C. Travis:** Investigation (supporting); writing – review and editing (supporting). **Timothy J. Key:** Investigation (supporting); writing – review and editing (supporting).

## FUNDING INFORMATION

This research was funded by Cancer Research UK Population Research Fellowship number C60192/A28516 to APC. APC is also supported by the World Cancer Research Fund (WCRF UK), as part of the Word Cancer Research Fund International grant programme (2019/1953). KS‐M, RT and TJK are supported by Cancer Research UK (C8221/A29017). EH is supported by a Cancer Research UK Population Research Committee Studentship (C18281/A30905). RMM is a National Institute for Health Research Senior Investigator (NIHR202411). EH, RMM and SJL are supported by a Cancer Research UK 25 (C18281/A29019) programme grant (the Integrative Cancer Epidemiology Programme). RMM is also supported by the NIHR Bristol Biomedical Research Centre which is funded by the NIHR (BRC‐1215‐20011) and is a partnership between University Hospitals Bristol and Weston NHS Foundation Trust and the University of Bristol. RMM and EH are affiliated with the Medical Research Council Integrative Epidemiology Unit at the University of Bristol which is supported by the Medical Research Council (MC_UU_00011/1, MC_UU_00011/3, MC_UU_00011/6 and MC_UU_00011/4) and the University of Bristol. Department of Health and Social Care disclaimer: The views expressed are those of the author(s) and not necessarily those of the NHS, the NIHR or the Department of Health and Social Care. SM is supported by an ‘Expanding excellence in England’ award from Research England and by the National Institute for Health and Care Research Exeter Biomedical Research Centre.

## CONFLICT OF INTEREST STATEMENT

The authors declare no competing interests.

## ETHICS STATEMENT

Each individual study obtained ethical approval, therefore additional ethical approval for this study was not required.

## Supporting information


Data S1.

## Data Availability

The data used in this analysis were obtained from the PRACTICAL Consortium, dbGAP (application Project # 31553), and GIANT and UK Biobank. These data are available to researchers upon application to each respective resource.
