# Supplementary material for: Genetic predisposition to metabolically unfavourable adiposity and prostate cancer risk: A Mendelian randomization analysis
Source: Cancer Med. 2023 Jun 12;12(15):16482–9. doi: 10.1002/cam4.6220 (PMC10469819; doi:10.1002/cam4.6220)
Supplement: Supplementary file 1 — Data S1. [file CAM4-12-16482-s001.docx]

**Supplementary Materials**

**Supplementary Tables**

[Supplementary Table 1: Association of unfavourable adiposity SNPs used in two-sample Mendelian randomization analyses with prostate cancer (- represents if SNPs not present in outcome dataset) 3](#_Toc132884200)

[Supplementary Table 2: Association of favourable adiposity SNPs used in two-sample Mendelian randomization analyses with prostate cancer (- represents if SNPs not present in outcome dataset) 5](#_Toc132884201)

[Supplementary Table 3: Association of BMI SNPs used in two-sample Mendelian randomization analyses with prostate cancer (- represents SNPs not present in outcome dataset) 7](#_Toc132884202)

**Supplementary Figures**

[Supplementary Figure 1: Traits associated with each metabolically unfavourable adiposity SNP used in the Mendelian randomization analyses. 22](#_Toc132884210)

[Supplementary Figure 2: Traits associated with each metabolically favourable adiposity SNP used in the Mendelian randomization analyses. 23](#_Toc132884211)

[Supplementary Figure 3: Traits associated with each BMI SNP used in the Mendelian randomization analyses. 24](#_Toc132884212)

[Supplementary Figure 4: Scatterplot of genetic associations with metabolically unfavourable adiposity against genetic associations with: A) Overall prostate cancer, B) Aggressive prostate cancer, C) Early-onset prostate cancer. 25](#_Toc132884213)

[Supplementary Figure 5: Scatterplot of genetic associations with metabolically favourable adiposity against genetic associations with: A) Overall prostate cancer, B) Aggressive prostate cancer, C) Early-onset prostate cancer. 26](#_Toc132884214)

[Supplementary Figure 6: Scatterplot of genetic associations with BMI against genetic associations with: A) Overall prostate cancer, B) Aggressive prostate cancer, C) Early-onset prostate cancer. 27](#_Toc132884215)

[Supplementary Figure 7: Leave-one-out analysis for MR examining the effect of unfavourable adiposity on overall prostate cancer. 28](#_Toc132884216)

[Supplementary Figure 8: Leave-one-out analysis for MR examining the effect of favourable adiposity on overall prostate cancer. 29](#_Toc132884217)

[Supplementary Figure 9: Leave-one-out analysis for MR examining the effect of BMI on overall prostate cancer. 30](#_Toc132884218)

[Supplementary Figure 10: Leave-one-out analysis for MR examining the effect of unfavourable adiposity on aggressive prostate cancer. 31](#_Toc132884219)

[Supplementary Figure 11: Leave-one-out analysis for MR examining the effect of favourable adiposity on aggressive prostate cancer. 32](#_Toc132884220)

[Supplementary Figure 12: Leave-one-out analysis for MR examining the effect of BMI on aggressive prostate cancer. 33](#_Toc132884221)

[Supplementary Figure 13: Leave-one-out analysis for MR examining the effect of unfavourable adiposity on early-onset prostate cancer. 34](#_Toc132884222)

[Supplementary Figure 14: Leave-one-out analysis for MR examining the effect of favourable adiposity on early-onset prostate cancer. 35](#_Toc132884223)

[Supplementary Figure 15: Leave-one-out analysis for MR examining the effect of BMI on early-onset prostate cancer. 36](#_Toc132884224)

[References 37](#_Toc132884225)

| **Supplementary Table 1:** Association of **unfavourable adiposity** SNPs used in two-sample Mendelian randomization analyses with prostate cancer (- represents if SNPs not present in outcome dataset) | | | | | | | | | | | | | | | | |
| --- | --- | --- | --- | --- | --- | --- | --- | --- | --- | --- | --- | --- | --- | --- | --- | --- |
|  |  |  |  |  | Association parameters with unfavourable adiposity | | | Association parameters with overall prostate cancer | | | Association parameters with aggressive prostate cancer | | | Association parameters with early onset prostate cancer | | |
| SNP | Chr | Position | Effect allele | Other allele | Effect | SE | P-value | Effect | SE | P-value | Effect | SE | P-value | Effect | SE | P-value |
| rs71658797 | 1 | 77967507 | T | A | -0.024 | 0.002 | 5.9E-26 | -0.005 | 0.012 | 0.356 | 0.001 | 0.022 | 0.963 | 0.023 | 0.032 | 0.480 |
| rs539515 | 1 | 177889025 | A | C | -0.028 | 0.002 | 9.2E-54 | -0.009 | 0.010 | 0.161 | -0.007 | 0.018 | 0.681 | -0.022 | 0.025 | 0.376 |
| rs11122450 | 1 | 230301811 | T | G | 0.010 | 0.002 | 6.4E-11 | 0.007 | 0.008 | 0.217 | -0.002 | 0.015 | 0.901 | 0.055 | 0.022 | 0.013 |
| rs6752378 | 2 | 25150116 | C | A | -0.023 | 0.001 | 7E-55 | -0.006 | 0.008 | 0.232 | - | - | - | - | - | - |
| rs1471740 | 3 | 136328270 | T | C | -0.010 | 0.002 | 4.4E-09 | -0.014 | 0.009 | 0.058 | -0.005 | 0.016 | 0.741 | -0.028 | 0.023 | 0.219 |
| rs10938397 | 4 | 45182527 | A | G | -0.020 | 0.001 | 7.1E-41 | -0.001 | 0.008 | 0.442 | -0.007 | 0.014 | 0.622 | -0.016 | 0.020 | 0.425 |
| rs13107325 | 4 | 103188709 | C | T | -0.031 | 0.003 | 2.9E-28 | 0.020 | 0.015 | 0.100 | - | - | - | - | - | - |
| rs2112347 | 5 | 75015242 | T | G | 0.018 | 0.002 | 2.2E-31 | -0.008 | 0.008 | 0.141 | -0.019 | 0.014 | 0.187 | 0.007 | 0.021 | 0.751 |
| rs17764730 | 5 | 127357526 | C | T | 0.012 | 0.002 | 7.5E-12 | 0.007 | 0.009 | 0.216 | -0.004 | 0.017 | 0.802 | 0.016 | 0.024 | 0.507 |
| rs9358912 | 6 | 26211146 | G | T | 0.022 | 0.002 | 4.6E-39 | -0.017 | 0.008 | 0.017 | -0.019 | 0.015 | 0.211 | -0.007 | 0.022 | 0.741 |
| rs72892910 | 6 | 50816887 | G | T | -0.023 | 0.002 | 1.8E-32 | 0.007 | 0.010 | 0.226 | 0.003 | 0.018 | 0.882 | 0.011 | 0.026 | 0.683 |
| rs236660 | 7 | 75050086 | T | C | -0.014 | 0.002 | 1.9E-19 | 0.016 | 0.008 | 0.020 | 0.014 | 0.015 | 0.337 | 0.024 | 0.021 | 0.238 |
| rs4876611 | 8 | 116671848 | A | G | -0.018 | 0.002 | 7E-28 | -0.021 | 0.008 | 0.006 | -0.015 | 0.015 | 0.314 | -0.014 | 0.022 | 0.532 |
| rs10756713 | 9 | 15880555 | A | G | 0.019 | 0.002 | 1.4E-37 | -0.005 | 0.008 | 0.257 | 0.003 | 0.014 | 0.839 | -0.009 | 0.020 | 0.657 |
| rs61888762 | 11 | 27709630 | C | G | -0.018 | 0.002 | 2.5E-29 | 0.014 | 0.008 | 0.040 | 0.014 | 0.015 | 0.338 | 0.005 | 0.022 | 0.835 |
| rs4755725 | 11 | 43637975 | C | A | 0.017 | 0.002 | 2.8E-26 | -0.002 | 0.008 | 0.406 | -0.003 | 0.015 | 0.841 | -0.005 | 0.021 | 0.811 |
| rs7124681 | 11 | 47529947 | C | A | -0.022 | 0.002 | 6.6E-50 | 0.020 | 0.008 | 0.004 | 0.002 | 0.014 | 0.878 | 0.021 | 0.021 | 0.323 |
| rs7132908 | 12 | 50263148 | G | A | -0.019 | 0.002 | 1.4E-36 | -0.012 | 0.008 | 0.070 | - | - | - | - | - | - |
| rs3764002 | 12 | 108618630 | C | T | 0.019 | 0.002 | 1.8E-29 | 0.008 | 0.010 | 0.199 | - | - | - | - | - | - |
| rs4776985 | 15 | 68123021 | T | G | 0.020 | 0.002 | 9.2E-31 | -0.014 | 0.009 | 0.064 | -0.009 | 0.017 | 0.600 | -0.015 | 0.024 | 0.538 |
| rs6602997 | 15 | 84521398 | C | T | -0.022 | 0.002 | 5.6E-43 | 0.009 | 0.008 | 0.153 | 0.009 | 0.015 | 0.573 | 0.001 | 0.022 | 0.974 |
| rs56186137 | 16 | 28825953 | A | G | -0.023 | 0.002 | 3.2E-50 | -0.002 | 0.008 | 0.384 | 0.006 | 0.014 | 0.653 | 0.015 | 0.021 | 0.475 |
| rs11642015 | 16 | 53802494 | C | T | -0.041 | 0.002 | 6.8E-165 | 0.013 | 0.008 | 0.044 | - | - | - | - | - | - |
| rs4790292 | 17 | 1824305 | C | A | 0.021 | 0.002 | 6E-24 | 0.000 | 0.011 | 0.486 | 0.024 | 0.021 | 0.242 | -0.004 | 0.030 | 0.892 |
| rs55931203 | 17 | 65854602 | C | T | -0.021 | 0.002 | 5.5E-28 | 0.027 | 0.010 | 0.003 | 0.008 | 0.018 | 0.666 | 0.077 | 0.026 | 0.003 |
| rs6567160 | 18 | 57829135 | T | C | -0.026 | 0.002 | 4.7E-48 | 0.016 | 0.009 | 0.034 | 0.014 | 0.017 | 0.393 | 0.036 | 0.024 | 0.131 |
| rs11666808 | 19 | 18383506 | T | C | 0.017 | 0.002 | 5.5E-27 | 0.005 | 0.008 | 0.267 | 0.010 | 0.015 | 0.494 | -0.032 | 0.021 | 0.128 |

| **Supplementary Table 2:** Association of **favourable adiposity** SNPs used in two-sample Mendelian randomization analyses with prostate cancer (- represents if SNPs not present in outcome dataset) | | | | | | | | | | | | | | | | |
| --- | --- | --- | --- | --- | --- | --- | --- | --- | --- | --- | --- | --- | --- | --- | --- | --- |
|  |  |  |  |  | Association parameters with favourable adiposity | | | Association parameters with overall prostate cancer | | | Association parameters with aggressive prostate cancer | | | Association parameters with early onset prostate cancer | | |
| SNP | Chr | Position | Effect allele | Other allele | Effect | SE | P-value | Effect | SE | P-value | Effect | SE | P-value | Effect | SE | P-value |
| rs2802774 | 1 | 203527812 | C | A | -0.011 | 0.002 | 3.6E-13 | -0.003 | 0.008 | 0.352 | 0.006 | 0.016 | 0.709 | -0.016 | 0.023 | 0.495 |
| rs12130231 | 1 | 219631304 | A | G | 0.020 | 0.002 | 4.2E-41 | -0.006 | 0.008 | 0.206 | 0.000 | 0.014 | 0.990 | -0.022 | 0.021 | 0.289 |
| rs13389219 | 2 | 165528876 | C | T | -0.017 | 0.002 | 2.2E-29 | 0.004 | 0.008 | 0.286 | -0.003 | 0.014 | 0.837 | 0.048 | 0.020 | 0.016 |
| rs2943653 | 2 | 227047771 | C | T | 0.016 | 0.002 | 1.3E-23 | -0.002 | 0.008 | 0.413 | 0.012 | 0.015 | 0.440 | -0.008 | 0.022 | 0.726 |
| rs4684847 | 3 | 12386337 | C | T | -0.029 | 0.002 | 2.7E-37 | -0.014 | 0.011 | 0.113 | 0.012 | 0.021 | 0.549 | -0.003 | 0.030 | 0.934 |
| rs9851766 | 3 | 138121509 | A | G | 0.014 | 0.002 | 1.3E-11 | -0.003 | 0.011 | 0.379 | 0.001 | 0.020 | 0.972 | 0.000 | 0.028 | 0.988 |
| rs62271373 | 3 | 150066540 | T | A | 0.024 | 0.003 | 2E-14 | -0.005 | 0.019 | 0.395 | 0.026 | 0.036 | 0.466 | -0.016 | 0.052 | 0.766 |
| rs4450871 | 4 | 4990298 | A | G | -0.009 | 0.001 | 1.1E-08 | 0.003 | 0.009 | 0.358 | 0.008 | 0.016 | 0.647 | -0.006 | 0.024 | 0.800 |
| rs13132853 | 4 | 38680015 | A | G | 0.009 | 0.002 | 9.6E-09 | -0.013 | 0.008 | 0.049 | -0.029 | 0.015 | 0.054 | -0.066 | 0.022 | 0.002 |
| rs30351 | 5 | 55794632 | G | A | 0.012 | 0.002 | 2.2E-12 | -0.011 | 0.009 | 0.115 | -0.021 | 0.016 | 0.196 | -0.016 | 0.024 | 0.509 |
| rs4976033 | 5 | 67714246 | A | G | 0.012 | 0.002 | 5E-14 | 0.005 | 0.008 | 0.290 | 0.008 | 0.015 | 0.616 | -0.004 | 0.023 | 0.865 |
| rs9764678 | 5 | 118726662 | T | C | -0.010 | 0.002 | 2.5E-10 | -0.002 | 0.008 | 0.411 | 0.013 | 0.015 | 0.394 | 0.039 | 0.023 | 0.088 |
| rs11135038 | 5 | 157930133 | T | G | -0.014 | 0.002 | 3.2E-16 | -0.009 | 0.009 | 0.161 | -0.017 | 0.016 | 0.284 | -0.021 | 0.023 | 0.354 |
| rs998584 | 6 | 43757896 | C | A | 0.009 | 0.001 | 1.4E-09 | 0.006 | 0.008 | 0.224 | - | - | - | - | - | - |
| rs72959041 | 6 | 127454893 | G | A | 0.023 | 0.003 | 1.8E-12 | -0.010 | 0.018 | 0.292 | -0.035 | 0.033 | 0.292 | -0.015 | 0.050 | 0.767 |
| rs573454216 | 6 | 139837429 | G | A | -0.009 | 0.002 | 1.2E-08 | -0.017 | 0.008 | 0.013 | - | - | - | - | - | - |
| rs972283 | 7 | 130466854 | A | G | 0.014 | 0.001 | 2.2E-22 | -0.004 | 0.008 | 0.300 | 0.002 | 0.015 | 0.869 | -0.020 | 0.022 | 0.357 |
| rs6977416 | 7 | 150542711 | G | A | 0.012 | 0.002 | 3.1E-14 | -0.002 | 0.008 | 0.420 | -0.004 | 0.014 | 0.764 | -0.023 | 0.021 | 0.274 |
| rs12681990 | 8 | 36859186 | T | C | 0.012 | 0.002 | 5.1E-09 | -0.002 | 0.010 | 0.425 | - | - | - | - | - | - |
| rs2980888 | 8 | 126507308 | T | C | -0.012 | 0.002 | 4.2E-14 | -0.013 | 0.009 | 0.066 | -0.004 | 0.016 | 0.818 | -0.008 | 0.023 | 0.721 |
| rs113222038 | 11 | 62380027 | C | T | 0.011 | 0.002 | 2.7E-12 | -0.004 | 0.009 | 0.331 | -0.012 | 0.016 | 0.452 | 0.013 | 0.024 | 0.572 |
| rs11045172 | 12 | 20470221 | A | C | -0.011 | 0.002 | 4.5E-09 | -0.010 | 0.010 | 0.156 | - | - | - | - | - | - |
| rs10876529 | 12 | 54421810 | T | C | -0.010 | 0.002 | 5.2E-12 | -0.008 | 0.008 | 0.171 | - | - | - | - | - | - |
| rs12369179 | 12 | 122963550 | C | T | 0.029 | 0.003 | 2.3E-29 | -0.014 | 0.014 | 0.153 | - | - | - | - | - | - |
| rs7133378 | 12 | 124409502 | G | A | -0.019 | 0.002 | 3.6E-33 | 0.017 | 0.008 | 0.020 | 0.024 | 0.015 | 0.114 | 0.047 | 0.022 | 0.034 |
| rs72697297 | 14 | 93069989 | T | C | 0.015 | 0.002 | 8.2E-15 | 0.016 | 0.010 | 0.059 | - | - | - | - | - | - |
| rs12441543 | 15 | 31689543 | G | A | -0.012 | 0.002 | 1.2E-12 | -0.011 | 0.009 | 0.108 | -0.046 | 0.015 | 0.003 | -0.018 | 0.022 | 0.412 |
| rs12940684 | 17 | 7453919 | C | T | 0.011 | 0.002 | 1.1E-11 | 0.007 | 0.008 | 0.200 | 0.001 | 0.015 | 0.957 | 0.009 | 0.022 | 0.676 |
| rs142186653 | 17 | 73879851 | A | C | -0.012 | 0.002 | 1.2E-11 | -0.004 | 0.010 | 0.337 | -0.011 | 0.018 | 0.567 | -0.023 | 0.027 | 0.381 |
| rs11664106 | 18 | 2846812 | A | T | -0.009 | 0.002 | 5.1E-09 | -0.006 | 0.009 | 0.257 | 0.006 | 0.016 | 0.738 | -0.015 | 0.024 | 0.531 |
| rs7233512 | 18 | 42595076 | G | A | 0.011 | 0.002 | 8.3E-11 | 0.006 | 0.008 | 0.243 | 0.002 | 0.015 | 0.897 | 0.008 | 0.022 | 0.732 |
| rs7258937 | 19 | 33938800 | C | T | -0.016 | 0.001 | 1.9E-26 | 0.005 | 0.008 | 0.239 | 0.014 | 0.014 | 0.336 | 0.007 | 0.020 | 0.724 |

| **Supplementary Table 3**: Association of **BMI SNPs** used in two-sample Mendelian randomization analyses with prostate cancer (- represents SNPs not present in outcome dataset) | | | | | | | | | | | | | | | | |
| --- | --- | --- | --- | --- | --- | --- | --- | --- | --- | --- | --- | --- | --- | --- | --- | --- |
|  |  |  |  |  | Association parameters with BMI | | | Association parameters with overall prostate cancer | | | Association parameters with aggressive prostate cancer | | | Association parameters with early onset prostate cancer | | |
| SNP | Chr | Position | Effect allele | Other allele | Effect | SE | P-value | Effect | SE | P-value | Effect | SE | P-value | Effect | SE | P-value |
| rs79113395 | 1 | 1590521 | G | A | 0.020 | 0.0022 | 2.03E-20 | 0.017 | 0.012 | 0.080 | 0.041 | 0.0220 | 0.063 | 0.036 | 0.0322 | 0.263 |
| rs7537581 | 1 | 2725475 | A | C | 0.014 | 0.0017 | 6.28E-16 | 0.012 | 0.008 | 0.068 | 0.007 | 0.0141 | 0.602 | -0.007 | 0.0204 | 0.749 |
| rs6577584 | 1 | 6715390 | G | T | 0.012 | 0.0018 | 3.37E-11 | -0.009 | 0.008 | 0.136 | -0.024 | 0.0149 | 0.103 | -0.024 | 0.0217 | 0.272 |
| rs1891215 | 1 | 7727854 | C | T | 0.011 | 0.0017 | 7.37E-11 | -0.009 | 0.008 | 0.127 | -0.002 | 0.0139 | 0.882 | -0.035 | 0.0202 | 0.087 |
| rs1884429 | 1 | 11112836 | T | C | 0.013 | 0.0019 | 2.3E-12 | -0.020 | 0.009 | 0.015 | -0.029 | 0.0169 | 0.090 | 0.006 | 0.0243 | 0.792 |
| rs3766160 | 1 | 15808872 | G | A | 0.011 | 0.0018 | 4.63E-09 | 0.004 | 0.009 | 0.321 | 0.025 | 0.0159 | 0.114 | -0.011 | 0.0230 | 0.638 |
| rs761423 | 1 | 17301672 | T | C | 0.011 | 0.0017 | 6.51E-10 | -0.002 | 0.008 | 0.395 | -0.006 | 0.0143 | 0.691 | -0.005 | 0.0206 | 0.809 |
| rs61740466 | 1 | 19934900 | G | A | 0.014 | 0.0022 | 2.24E-10 | 0.006 | 0.009 | 0.274 | 0.005 | 0.0171 | 0.787 | -0.022 | 0.0252 | 0.393 |
| rs561136 | 1 | 23345051 | C | T | 0.019 | 0.0024 | 1.43E-14 | 0.013 | 0.011 | 0.134 | 0.007 | 0.0209 | 0.748 | -0.001 | 0.0302 | 0.968 |
| rs12022461 | 1 | 33232525 | G | A | 0.017 | 0.0022 | 9.78E-14 | -0.013 | 0.010 | 0.098 | 0.004 | 0.0182 | 0.842 | -0.038 | 0.0264 | 0.156 |
| rs11577094 | 1 | 38026600 | T | C | 0.019 | 0.0030 | 3.28E-10 | 0.033 | 0.014 | 0.008 | 0.045 | 0.0251 | 0.075 | 0.095 | 0.0357 | 0.008 |
| rs112566467 | 1 | 39562627 | T | C | 0.018 | 0.0024 | 5.86E-15 | 0.024 | 0.009 | 0.006 | 0.009 | 0.0172 | 0.594 | 0.048 | 0.0248 | 0.054 |
| rs7523668 | 1 | 42408159 | G | A | 0.011 | 0.0017 | 7.24E-10 | -0.008 | 0.008 | 0.152 | 0.003 | 0.0139 | 0.850 | -0.010 | 0.0203 | 0.611 |
| rs1707322 | 1 | 46505147 | G | A | 0.014 | 0.0018 | 1.59E-14 | 0.031 | 0.008 | <0.001 | 0.041 | 0.0151 | 0.007 | 0.023 | 0.0216 | 0.289 |
| rs2070929 | 1 | 47683607 | C | G | 0.014 | 0.0019 | 3.32E-13 | -0.008 | 0.009 | 0.170 | -0.007 | 0.0157 | 0.680 | -0.014 | 0.0225 | 0.534 |
| rs12121950 | 1 | 49710264 | T | G | 0.019 | 0.0018 | 7.09E-24 | 0.002 | 0.009 | 0.407 | -0.013 | 0.0157 | 0.405 | -0.038 | 0.0228 | 0.095 |
| rs630602 | 1 | 54728864 | C | G | 0.013 | 0.0018 | 1.06E-12 | 0.012 | 0.008 | 0.060 | 0.020 | 0.0145 | 0.179 | 0.011 | 0.0210 | 0.613 |
| rs17425707 | 1 | 57874879 | C | T | 0.017 | 0.0028 | 6.16E-10 | 0.005 | 0.013 | 0.361 | 0.021 | 0.0237 | 0.381 | -0.023 | 0.0347 | 0.499 |
| rs12140153 | 1 | 62579891 | G | T | 0.035 | 0.0034 | 1.44E-25 | -0.020 | 0.014 | 0.076 | -0.057 | 0.0255 | 0.025 | -0.006 | 0.0379 | 0.880 |
| rs6690398 | 1 | 66447394 | A | G | 0.013 | 0.0017 | 1.92E-13 | 0.002 | 0.008 | 0.384 | 0.001 | 0.0143 | 0.919 | 0.001 | 0.0208 | 0.957 |
| rs3101336 | 1 | 72751185 | C | T | 0.025 | 0.0016 | 4.8E-54 | -0.002 | 0.008 | 0.409 | -0.031 | 0.0143 | 0.031 | -0.035 | 0.0206 | 0.088 |
| rs12049202 | 1 | 77967523 | T | C | 0.024 | 0.0021 | 3.35E-29 | 0.018 | 0.010 | 0.030 | 0.019 | 0.0174 | 0.268 | -0.012 | 0.0256 | 0.629 |
| rs6696828 | 1 | 80812020 | C | G | 0.012 | 0.0018 | 6.42E-11 | -0.001 | 0.009 | 0.451 | -0.005 | 0.0162 | 0.762 | 0.009 | 0.0234 | 0.704 |
| rs284227 | 1 | 82379446 | C | T | 0.015 | 0.0019 | 6.08E-15 | 0.013 | 0.009 | 0.067 | 0.012 | 0.0155 | 0.459 | 0.016 | 0.0227 | 0.491 |
| rs6604015 | 1 | 93219907 | C | T | 0.018 | 0.0027 | 1.18E-11 | -0.016 | 0.012 | 0.094 | -0.001 | 0.0223 | 0.961 | -0.012 | 0.0326 | 0.707 |
| rs2968487 | 1 | 96887370 | T | C | 0.018 | 0.0018 | 7.55E-22 | -0.001 | 0.009 | 0.445 | 0.024 | 0.0160 | 0.131 | 0.005 | 0.0233 | 0.831 |
| rs12072739 | 1 | 98315893 | G | A | 0.017 | 0.0023 | 1.5E-13 | -0.009 | 0.009 | 0.167 | -0.016 | 0.0164 | 0.321 | 0.028 | 0.0239 | 0.250 |
| rs1730859 | 1 | 107617707 | G | A | 0.012 | 0.0017 | 2.96E-12 | 0.004 | 0.008 | 0.315 | -0.002 | 0.0150 | 0.905 | 0.003 | 0.0213 | 0.905 |
| rs17024393 | 1 | 110154688 | C | T | 0.064 | 0.0049 | 7.11E-39 | 0.035 | 0.022 | 0.056 | - | - | - | - | - | - |
| rs1546924 | 1 | 112273485 | T | C | 0.014 | 0.0016 | 2.34E-17 | -0.004 | 0.008 | 0.325 | - | - | - | - | - | - |
| rs10923724 | 1 | 119546842 | C | T | 0.013 | 0.0016 | 1.14E-14 | -0.004 | 0.008 | 0.281 | 0.006 | 0.0139 | 0.684 | 0.009 | 0.0200 | 0.656 |
| rs3738476 | 1 | 151006539 | C | A | 0.020 | 0.0026 | 3.35E-14 | -0.024 | 0.009 | 0.006 | -0.014 | 0.0174 | 0.417 | -0.056 | 0.0251 | 0.026 |
| rs61813324 | 1 | 156049877 | T | C | 0.029 | 0.0028 | 3.2E-24 | -0.042 | 0.013 | 0.000 | 0.005 | 0.0227 | 0.830 | -0.002 | 0.0340 | 0.949 |
| rs34720381 | 1 | 171455322 | T | C | 0.023 | 0.0033 | 7.89E-12 | -0.007 | 0.014 | 0.297 | -0.015 | 0.0250 | 0.544 | 0.050 | 0.0350 | 0.155 |
| rs61828641 | 1 | 174321997 | A | G | 0.022 | 0.0030 | 2.49E-13 | 0.000 | 0.012 | 0.490 | 0.040 | 0.0220 | 0.067 | 0.040 | 0.0313 | 0.199 |
| rs543874 | 1 | 177889480 | G | A | 0.048 | 0.0020 | 3.06E-125 | 0.010 | 0.010 | 0.149 | 0.010 | 0.0175 | 0.567 | 0.020 | 0.0250 | 0.434 |
| rs10797987 | 1 | 184651822 | C | T | 0.012 | 0.0019 | 7.69E-10 | 0.007 | 0.008 | 0.172 | -0.011 | 0.0140 | 0.448 | 0.043 | 0.0202 | 0.035 |
| rs10920678 | 1 | 190239907 | A | G | 0.015 | 0.0016 | 7.15E-20 | -0.008 | 0.008 | 0.141 | -0.023 | 0.0143 | 0.109 | -0.007 | 0.0208 | 0.733 |
| rs2400414 | 1 | 194965200 | C | T | 0.013 | 0.0018 | 5.52E-13 | -0.005 | 0.008 | 0.247 | -0.002 | 0.0144 | 0.873 | -0.028 | 0.0208 | 0.180 |
| rs1009188 | 1 | 197257090 | T | C | 0.012 | 0.0019 | 7.15E-11 | -0.013 | 0.008 | 0.059 | -0.008 | 0.0150 | 0.617 | -0.066 | 0.0217 | 0.002 |
| rs2820295 | 1 | 201800868 | A | G | 0.024 | 0.0018 | 5.56E-39 | -0.006 | 0.008 | 0.245 | -0.014 | 0.0149 | 0.349 | 0.020 | 0.0214 | 0.353 |
| rs13303252 | 1 | 203485142 | T | C | 0.015 | 0.0025 | 8.56E-10 | 0.000 | 0.012 | 0.497 | -0.005 | 0.0230 | 0.842 | -0.018 | 0.0338 | 0.600 |
| rs6661316 | 1 | 210095527 | T | C | 0.012 | 0.0016 | 1.72E-13 | 0.003 | 0.008 | 0.329 | 0.016 | 0.0142 | 0.254 | 0.002 | 0.0206 | 0.916 |
| rs946824 | 1 | 243684019 | T | C | 0.020 | 0.0025 | 4.82E-15 | 0.001 | 0.012 | 0.462 | 0.020 | 0.0209 | 0.337 | -0.022 | 0.0306 | 0.479 |
| rs13021737 | 2 | 632348 | G | A | 0.058 | 0.0021 | 2.89E-161 | -0.027 | 0.010 | 0.004 | -0.036 | 0.0183 | 0.048 | 0.035 | 0.0269 | 0.196 |
| rs10929925 | 2 | 6155557 | C | A | 0.014 | 0.0016 | 3.05E-18 | -0.013 | 0.008 | 0.047 | -0.030 | 0.0141 | 0.033 | 0.010 | 0.0206 | 0.633 |
| rs7607490 | 2 | 12851120 | A | G | 0.016 | 0.0027 | 1.21E-09 | -0.002 | 0.013 | 0.448 | -0.020 | 0.0241 | 0.401 | -0.002 | 0.0334 | 0.964 |
| rs12470698 | 2 | 27063261 | A | G | 0.010 | 0.0017 | 1.99E-09 | 0.001 | 0.008 | 0.458 | -0.008 | 0.0139 | 0.568 | -0.010 | 0.0201 | 0.638 |
| rs4372836 | 2 | 28973883 | T | C | 0.013 | 0.0017 | 7.33E-13 | -0.018 | 0.008 | 0.016 | -0.021 | 0.0150 | 0.155 | 0.001 | 0.0216 | 0.958 |
| rs1561554 | 2 | 35409697 | G | C | 0.012 | 0.0017 | 4.89E-11 | 0.010 | 0.008 | 0.093 | 0.016 | 0.0143 | 0.272 | -0.003 | 0.0207 | 0.891 |
| rs3770799 | 2 | 36788616 | G | A | 0.011 | 0.0018 | 1.21E-10 | 0.008 | 0.008 | 0.153 | 0.004 | 0.0146 | 0.801 | 0.010 | 0.0210 | 0.652 |
| rs985060 | 2 | 40284930 | G | A | 0.012 | 0.0019 | 8.56E-10 | 0.008 | 0.008 | 0.159 | 0.005 | 0.0156 | 0.770 | 0.025 | 0.0223 | 0.258 |
| rs2063177 | 2 | 41712849 | A | G | 0.012 | 0.0018 | 1.87E-11 | 0.004 | 0.008 | 0.295 | -0.013 | 0.0148 | 0.387 | -0.001 | 0.0216 | 0.956 |
| rs786420 | 2 | 44719893 | T | C | 0.014 | 0.0020 | 2.27E-12 | -0.010 | 0.009 | 0.125 | -0.031 | 0.0159 | 0.051 | 0.013 | 0.0232 | 0.586 |
| rs35809007 | 2 | 47019521 | G | A | 0.016 | 0.0020 | 9E-16 | -0.002 | 0.008 | 0.400 | 0.000 | 0.0145 | 0.987 | 0.041 | 0.0211 | 0.050 |
| rs7561278 | 2 | 48954905 | T | C | 0.017 | 0.0021 | 4.89E-16 | 0.023 | 0.009 | 0.007 | - | - | - | - | - | - |
| rs930295 | 2 | 50233352 | A | C | 0.021 | 0.0023 | 2.03E-19 | -0.008 | 0.010 | 0.224 | 0.005 | 0.0186 | 0.774 | -0.063 | 0.0270 | 0.019 |
| rs7601895 | 2 | 55281901 | C | G | 0.015 | 0.0018 | 1.89E-16 | 0.005 | 0.008 | 0.269 | 0.017 | 0.0152 | 0.253 | -0.007 | 0.0220 | 0.742 |
| rs13432055 | 2 | 56603985 | C | T | 0.012 | 0.0018 | 5.72E-11 | -0.001 | 0.008 | 0.438 | - | - | - | - | - | - |
| rs4671328 | 2 | 58935282 | T | G | 0.021 | 0.0017 | 3.42E-36 | 0.009 | 0.008 | 0.132 | - | - | - | - | - | - |
| rs1011407 | 2 | 60665768 | A | G | 0.017 | 0.0026 | 6.81E-11 | 0.001 | 0.012 | 0.459 | 0.043 | 0.0220 | 0.049 | 0.013 | 0.0315 | 0.671 |
| rs13417156 | 2 | 62848319 | C | T | 0.014 | 0.0017 | 6.9E-16 | 0.068 | 0.008 | 0.000 | 0.072 | 0.0137 | 0.000 | 0.095 | 0.0198 | 0.000 |
| rs2861685 | 2 | 67837553 | T | C | 0.017 | 0.0019 | 7.82E-18 | 0.019 | 0.008 | 0.006 | 0.031 | 0.0141 | 0.026 | 0.008 | 0.0204 | 0.684 |
| rs4581940 | 2 | 69655790 | T | C | 0.010 | 0.0017 | 3.43E-09 | -0.005 | 0.008 | 0.239 | -0.001 | 0.0140 | 0.920 | 0.041 | 0.0203 | 0.041 |
| rs934515 | 2 | 79482643 | A | G | 0.018 | 0.0026 | 6.64E-12 | 0.006 | 0.012 | 0.315 | -0.006 | 0.0212 | 0.779 | 0.042 | 0.0307 | 0.167 |
| rs11126822 | 2 | 81792736 | A | G | 0.011 | 0.0018 | 1.88E-09 | 0.005 | 0.008 | 0.256 | 0.024 | 0.0148 | 0.098 | 0.010 | 0.0215 | 0.643 |
| rs12714199 | 2 | 86812549 | C | T | 0.014 | 0.0017 | 3.22E-16 | -0.001 | 0.008 | 0.450 | -0.004 | 0.0148 | 0.768 | 0.017 | 0.0212 | 0.433 |
| rs13002946 | 2 | 100801959 | T | A | 0.018 | 0.0019 | 3.9E-20 | 0.007 | 0.009 | 0.226 | 0.006 | 0.0164 | 0.735 | 0.008 | 0.0233 | 0.721 |
| rs10197031 | 2 | 105454590 | C | T | 0.016 | 0.0019 | 5.05E-18 | -0.005 | 0.009 | 0.279 | 0.002 | 0.0157 | 0.893 | -0.037 | 0.0232 | 0.107 |
| rs13033310 | 2 | 133523605 | A | G | 0.015 | 0.0022 | 3.4E-11 | -0.005 | 0.009 | 0.275 | -0.012 | 0.0161 | 0.460 | -0.016 | 0.0234 | 0.503 |
| rs13396415 | 2 | 142831758 | C | A | 0.015 | 0.0023 | 4.86E-11 | -0.003 | 0.010 | 0.376 | 0.005 | 0.0178 | 0.788 | 0.007 | 0.0263 | 0.799 |
| rs6710871 | 2 | 143960593 | A | G | 0.019 | 0.0024 | 3.07E-16 | 0.009 | 0.011 | 0.214 | -0.001 | 0.0204 | 0.958 | 0.015 | 0.0289 | 0.607 |
| rs7560871 | 2 | 145616899 | A | G | 0.022 | 0.0034 | 6.12E-11 | -0.019 | 0.015 | 0.094 | - | - | - | - | - | - |
| rs1451077 | 2 | 147901207 | G | A | 0.017 | 0.0019 | 1.43E-18 | 0.010 | 0.008 | 0.108 | 0.007 | 0.0149 | 0.630 | -0.007 | 0.0219 | 0.750 |
| rs10198345 | 2 | 157109395 | C | T | 0.011 | 0.0018 | 1.27E-09 | 0.005 | 0.008 | 0.264 | 0.014 | 0.0148 | 0.348 | -0.017 | 0.0215 | 0.440 |
| rs12692596 | 2 | 161265910 | T | C | 0.012 | 0.0017 | 1.03E-12 | -0.005 | 0.008 | 0.268 | -0.031 | 0.0145 | 0.031 | -0.008 | 0.0209 | 0.712 |
| rs61051952 | 2 | 166148324 | A | G | 0.015 | 0.0022 | 6.81E-12 | 0.000 | 0.009 | 0.482 | -0.005 | 0.0160 | 0.776 | 0.049 | 0.0235 | 0.038 |
| rs10930502 | 2 | 172890588 | A | G | 0.013 | 0.0018 | 2.74E-13 | -0.005 | 0.008 | 0.275 | 0.003 | 0.0151 | 0.869 | -0.009 | 0.0217 | 0.696 |
| rs2044469 | 2 | 174961488 | G | A | 0.013 | 0.0018 | 1.19E-13 | 0.017 | 0.008 | 0.021 | 0.021 | 0.0150 | 0.160 | 0.013 | 0.0217 | 0.552 |
| rs7588437 | 2 | 181575281 | G | A | 0.017 | 0.0017 | 2.32E-22 | -0.010 | 0.008 | 0.110 | 0.013 | 0.0145 | 0.375 | -0.016 | 0.0208 | 0.445 |
| rs7575118 | 2 | 182653725 | T | C | 0.014 | 0.0024 | 3.33E-09 | -0.004 | 0.012 | 0.384 | -0.057 | 0.0221 | 0.010 | -0.009 | 0.0316 | 0.785 |
| rs7570446 | 2 | 193801010 | A | C | 0.011 | 0.0019 | 4.74E-09 | -0.002 | 0.008 | 0.374 | 0.016 | 0.0137 | 0.237 | -0.008 | 0.0198 | 0.704 |
| rs6733834 | 2 | 198518709 | C | G | 0.015 | 0.0021 | 1.54E-12 | 0.005 | 0.010 | 0.301 | 0.024 | 0.0179 | 0.190 | -0.012 | 0.0255 | 0.641 |
| rs10497870 | 2 | 203970283 | A | G | 0.012 | 0.0016 | 1.97E-13 | -0.002 | 0.008 | 0.381 | 0.014 | 0.0138 | 0.314 | 0.010 | 0.0202 | 0.636 |
| rs1470545 | 2 | 205365851 | T | C | 0.037 | 0.0043 | 5.6E-18 | -0.013 | 0.020 | 0.260 | 0.009 | 0.0365 | 0.811 | 0.043 | 0.0517 | 0.409 |
| rs11692326 | 2 | 208263279 | T | C | 0.015 | 0.0019 | 1.69E-14 | 0.029 | 0.009 | 0.001 | 0.040 | 0.0164 | 0.016 | 0.029 | 0.0238 | 0.229 |
| rs715 | 2 | 211543055 | C | T | 0.016 | 0.0019 | 1.34E-16 | 0.013 | 0.008 | 0.067 | - | - | - | - | - | - |
| rs7599312 | 2 | 213413231 | G | A | 0.018 | 0.0018 | 1.52E-23 | 0.005 | 0.009 | 0.280 | 0.004 | 0.0156 | 0.797 | 0.027 | 0.0228 | 0.233 |
| rs7589023 | 2 | 220138586 | T | C | 0.017 | 0.0023 | 2.07E-14 | 0.009 | 0.011 | 0.205 | -0.016 | 0.0197 | 0.424 | 0.011 | 0.0277 | 0.684 |
| rs4973618 | 2 | 229002620 | G | A | 0.015 | 0.0018 | 1.25E-16 | 0.004 | 0.008 | 0.334 | -0.004 | 0.0156 | 0.780 | 0.029 | 0.0227 | 0.209 |
| rs6720868 | 2 | 230663576 | T | C | 0.015 | 0.0018 | 1.82E-17 | -0.004 | 0.008 | 0.293 | -0.012 | 0.0148 | 0.412 | -0.027 | 0.0214 | 0.202 |
| rs10179086 | 2 | 232744209 | C | T | 0.011 | 0.0020 | 2.48E-08 | 0.002 | 0.009 | 0.421 | 0.001 | 0.0166 | 0.953 | 0.016 | 0.0239 | 0.517 |
| rs987071 | 2 | 236786086 | C | T | 0.013 | 0.0020 | 7.91E-10 | 0.005 | 0.009 | 0.287 | 0.013 | 0.0163 | 0.429 | -0.034 | 0.0238 | 0.158 |
| rs59302296 | 3 | 9507314 | A | T | 0.022 | 0.0032 | 9.12E-12 | -0.026 | 0.013 | 0.021 | -0.024 | 0.0236 | 0.302 | -0.030 | 0.0340 | 0.379 |
| rs10510419 | 3 | 12426936 | G | T | 0.017 | 0.0023 | 2.23E-13 | -0.020 | 0.011 | 0.034 | -0.009 | 0.0200 | 0.658 | 0.009 | 0.0290 | 0.759 |
| rs4857968 | 3 | 20714580 | G | A | 0.013 | 0.0018 | 6.5E-12 | -0.012 | 0.008 | 0.080 | -0.023 | 0.0152 | 0.133 | 0.002 | 0.0220 | 0.919 |
| rs6804842 | 3 | 25106437 | G | A | 0.014 | 0.0016 | 7.57E-18 | -0.003 | 0.008 | 0.345 | -0.008 | 0.0148 | 0.595 | 0.011 | 0.0216 | 0.598 |
| rs13062093 | 3 | 35667057 | G | T | 0.012 | 0.0017 | 2.78E-12 | -0.021 | 0.008 | 0.005 | -0.025 | 0.0145 | 0.081 | 0.010 | 0.0209 | 0.619 |
| rs9816029 | 3 | 41311362 | C | G | 0.011 | 0.0018 | 2.63E-09 | 0.014 | 0.008 | 0.049 | 0.004 | 0.0152 | 0.772 | 0.004 | 0.0221 | 0.858 |
| rs9839267 | 3 | 42332624 | T | G | 0.023 | 0.0027 | 1.79E-17 | -0.018 | 0.013 | 0.079 | -0.032 | 0.0237 | 0.176 | -0.041 | 0.0348 | 0.242 |
| rs55676934 | 3 | 45222958 | G | A | 0.014 | 0.0020 | 5.19E-12 | 0.007 | 0.009 | 0.208 | 0.009 | 0.0155 | 0.551 | 0.027 | 0.0226 | 0.236 |
| rs1916801 | 3 | 61187046 | A | T | 0.017 | 0.0017 | 1.93E-24 | 0.007 | 0.008 | 0.202 | 0.013 | 0.0142 | 0.355 | 0.007 | 0.0206 | 0.730 |
| rs11915371 | 3 | 70539559 | C | A | 0.015 | 0.0021 | 2.29E-13 | 0.001 | 0.009 | 0.461 | 0.016 | 0.0167 | 0.350 | -0.008 | 0.0244 | 0.752 |
| rs1523768 | 3 | 77667044 | G | A | 0.013 | 0.0017 | 4.35E-13 | -0.019 | 0.009 | 0.016 | -0.022 | 0.0157 | 0.156 | -0.042 | 0.0228 | 0.068 |
| rs6781254 | 3 | 80649139 | T | C | 0.011 | 0.0018 | 4.56E-09 | -0.003 | 0.008 | 0.348 | 0.003 | 0.0148 | 0.822 | 0.022 | 0.0216 | 0.310 |
| rs3849570 | 3 | 81792112 | A | C | 0.013 | 0.0017 | 7.35E-15 | -0.005 | 0.008 | 0.254 | 0.011 | 0.0145 | 0.461 | -0.019 | 0.0210 | 0.374 |
| rs9818122 | 3 | 85861064 | C | T | 0.023 | 0.0020 | 3.97E-30 | -0.006 | 0.009 | 0.248 | -0.023 | 0.0173 | 0.183 | 0.014 | 0.0247 | 0.563 |
| rs1580099 | 3 | 94003603 | C | A | 0.018 | 0.0017 | 6.77E-27 | -0.007 | 0.008 | 0.179 | -0.006 | 0.0137 | 0.656 | 0.013 | 0.0199 | 0.509 |
| rs4273371 | 3 | 108119071 | C | T | 0.011 | 0.0016 | 3.8E-12 | -0.012 | 0.008 | 0.054 | -0.012 | 0.0139 | 0.377 | -0.013 | 0.0201 | 0.524 |
| rs17681451 | 3 | 114399296 | G | A | 0.023 | 0.0031 | 7.37E-13 | 0.026 | 0.014 | 0.033 | -0.016 | 0.0260 | 0.542 | 0.001 | 0.0373 | 0.978 |
| rs6804181 | 3 | 116937546 | A | T | 0.014 | 0.0023 | 6.47E-10 | 0.001 | 0.010 | 0.452 | -0.023 | 0.0182 | 0.206 | 0.019 | 0.0268 | 0.473 |
| rs779206 | 3 | 118023515 | G | A | 0.012 | 0.0019 | 3.28E-10 | -0.028 | 0.009 | 0.001 | -0.014 | 0.0164 | 0.407 | -0.003 | 0.0235 | 0.893 |
| rs16834431 | 3 | 123285856 | T | C | 0.014 | 0.0021 | 2.7E-11 | 0.009 | 0.010 | 0.188 | -0.008 | 0.0175 | 0.660 | 0.003 | 0.0248 | 0.913 |
| rs76594121 | 3 | 128189391 | T | G | 0.028 | 0.0047 | 1.64E-09 | -0.103 | 0.017 | 0.000 | - | - | - | - | - | - |
| rs7631156 | 3 | 131751628 | A | G | 0.022 | 0.0018 | 3.33E-32 | -0.010 | 0.008 | 0.115 | 0.007 | 0.0155 | 0.656 | -0.035 | 0.0226 | 0.121 |
| rs7621025 | 3 | 136272246 | C | T | 0.019 | 0.0019 | 7.54E-24 | 0.014 | 0.009 | 0.055 | 0.007 | 0.0160 | 0.677 | 0.029 | 0.0227 | 0.202 |
| rs1199334 | 3 | 138091140 | A | G | 0.015 | 0.0021 | 1.5E-12 | 0.003 | 0.010 | 0.369 | 0.007 | 0.0187 | 0.702 | -0.013 | 0.0279 | 0.655 |
| rs16851483 | 3 | 141275436 | T | G | 0.035 | 0.0034 | 4.87E-25 | 0.006 | 0.015 | 0.360 | 0.040 | 0.0280 | 0.151 | -0.032 | 0.0404 | 0.428 |
| rs355777 | 3 | 154034950 | C | G | 0.015 | 0.0017 | 2.13E-18 | 0.011 | 0.008 | 0.079 | - | - | - | - | - | - |
| rs6809307 | 3 | 156862041 | T | C | 0.014 | 0.0019 | 8.15E-13 | 0.000 | 0.009 | 0.486 | 0.000 | 0.0161 | 0.985 | 0.067 | 0.0228 | 0.003 |
| rs2682406 | 3 | 158026489 | T | A | 0.013 | 0.0017 | 4.76E-13 | -0.001 | 0.008 | 0.448 | - | - | - | - | - | - |
| rs507856 | 3 | 161453946 | T | C | 0.011 | 0.0017 | 2.61E-10 | 0.000 | 0.008 | 0.500 | 0.006 | 0.0142 | 0.654 | 0.006 | 0.0207 | 0.778 |
| rs39654 | 3 | 173095123 | G | A | 0.016 | 0.0017 | 1.75E-21 | 0.007 | 0.008 | 0.214 | 0.014 | 0.0148 | 0.331 | 0.018 | 0.0217 | 0.399 |
| rs6443750 | 3 | 181329682 | C | T | 0.015 | 0.0021 | 7.25E-13 | 0.001 | 0.011 | 0.461 | -0.004 | 0.0206 | 0.855 | -0.028 | 0.0303 | 0.356 |
| rs3752904 | 3 | 183996068 | C | T | 0.012 | 0.0016 | 8.9E-13 | -0.025 | 0.008 | 0.001 | -0.038 | 0.0139 | 0.006 | -0.051 | 0.0202 | 0.011 |
| rs9816226 | 3 | 185834499 | T | A | 0.032 | 0.0021 | 1.45E-50 | -0.013 | 0.010 | 0.095 | -0.033 | 0.0182 | 0.070 | 0.009 | 0.0262 | 0.741 |
| rs7616009 | 3 | 194881756 | G | A | 0.016 | 0.0024 | 4.33E-11 | -0.010 | 0.011 | 0.186 | 0.016 | 0.0207 | 0.445 | 0.020 | 0.0296 | 0.498 |
| rs34801745 | 3 | 196115497 | C | G | 0.013 | 0.0020 | 7E-11 | -0.014 | 0.008 | 0.039 | -0.014 | 0.0147 | 0.333 | -0.004 | 0.0213 | 0.843 |
| rs2051559 | 4 | 3298800 | C | T | 0.017 | 0.0025 | 3.78E-11 | 0.005 | 0.011 | 0.347 | 0.024 | 0.0207 | 0.256 | 0.018 | 0.0297 | 0.554 |
| rs6818414 | 4 | 16600664 | C | T | 0.010 | 0.0017 | 4.57E-09 | -0.005 | 0.008 | 0.244 | 0.006 | 0.0144 | 0.698 | -0.002 | 0.0208 | 0.926 |
| rs1477890 | 4 | 18511738 | G | A | 0.013 | 0.0017 | 6.12E-14 | -0.004 | 0.008 | 0.286 | 0.009 | 0.0139 | 0.534 | -0.008 | 0.0201 | 0.707 |
| rs73249175 | 4 | 20219595 | G | T | 0.022 | 0.0029 | 1.62E-14 | 0.022 | 0.011 | 0.026 | 0.032 | 0.0202 | 0.113 | -0.007 | 0.0296 | 0.818 |
| rs34811474 | 4 | 25408838 | G | A | 0.029 | 0.0023 | 8.5E-38 | 0.025 | 0.010 | 0.008 | 0.005 | 0.0189 | 0.791 | 0.043 | 0.0279 | 0.128 |
| rs73213501 | 4 | 28514830 | A | C | 0.019 | 0.0025 | 1.08E-14 | 0.001 | 0.010 | 0.471 | -0.003 | 0.0175 | 0.864 | 0.016 | 0.0260 | 0.533 |
| rs4270551 | 4 | 30703243 | A | C | 0.017 | 0.0026 | 8.27E-12 | -0.001 | 0.012 | 0.466 | 0.014 | 0.0220 | 0.530 | -0.013 | 0.0311 | 0.681 |
| rs1000096 | 4 | 38692835 | C | T | 0.015 | 0.0018 | 2.34E-16 | -0.019 | 0.008 | 0.008 | -0.030 | 0.0145 | 0.041 | -0.057 | 0.0210 | 0.006 |
| rs10938397 | 4 | 45182527 | G | A | 0.032 | 0.0016 | 2.42E-86 | 0.001 | 0.008 | 0.442 | 0.007 | 0.0138 | 0.622 | 0.016 | 0.0200 | 0.425 |
| rs2192158 | 4 | 55505360 | A | G | 0.014 | 0.0017 | 7.42E-16 | -0.004 | 0.008 | 0.313 | 0.005 | 0.0144 | 0.711 | -0.004 | 0.0210 | 0.857 |
| rs1119950 | 4 | 60228517 | G | A | 0.011 | 0.0019 | 1.27E-09 | -0.002 | 0.008 | 0.399 | -0.001 | 0.0151 | 0.970 | 0.019 | 0.0219 | 0.379 |
| rs1346841 | 4 | 65651730 | G | A | 0.013 | 0.0017 | 3.18E-13 | -0.010 | 0.008 | 0.106 | -0.004 | 0.0147 | 0.801 | -0.006 | 0.0214 | 0.768 |
| rs10002111 | 4 | 67815504 | A | G | 0.013 | 0.0021 | 1.26E-09 | 0.003 | 0.009 | 0.354 | 0.006 | 0.0168 | 0.712 | 0.033 | 0.0242 | 0.179 |
| rs17001561 | 4 | 77096118 | A | G | 0.015 | 0.0023 | 1.81E-10 | -0.001 | 0.011 | 0.478 | -0.002 | 0.0198 | 0.931 | -0.013 | 0.0285 | 0.637 |
| rs35851183 | 4 | 80717182 | G | A | 0.012 | 0.0020 | 1.07E-09 | 0.004 | 0.008 | 0.329 | 0.006 | 0.0143 | 0.660 | 0.040 | 0.0206 | 0.053 |
| rs1481012 | 4 | 89039082 | A | G | 0.019 | 0.0026 | 5.11E-13 | 0.024 | 0.012 | 0.028 | 0.026 | 0.0225 | 0.243 | 0.001 | 0.0318 | 0.981 |
| rs2870710 | 4 | 94411606 | C | G | 0.016 | 0.0022 | 9.41E-13 | 0.006 | 0.010 | 0.268 | 0.021 | 0.0182 | 0.252 | -0.012 | 0.0266 | 0.649 |
| rs2241743 | 4 | 96091524 | G | A | 0.011 | 0.0016 | 8.57E-11 | -0.002 | 0.008 | 0.413 | -0.004 | 0.0141 | 0.774 | -0.007 | 0.0204 | 0.733 |
| rs13107325 | 4 | 103188709 | T | C | 0.047 | 0.0032 | 3.81E-47 | -0.020 | 0.015 | 0.100 | - | - | - | - | - | - |
| rs326845 | 4 | 112747919 | G | A | 0.012 | 0.0018 | 3.23E-11 | 0.002 | 0.008 | 0.395 | 0.004 | 0.0144 | 0.775 | 0.008 | 0.0208 | 0.692 |
| rs12509234 | 4 | 120319434 | C | T | 0.012 | 0.0019 | 3.89E-10 | -0.008 | 0.009 | 0.173 | -0.014 | 0.0158 | 0.386 | -0.045 | 0.0228 | 0.049 |
| rs2391540 | 4 | 130726833 | T | A | 0.014 | 0.0018 | 1.36E-14 | -0.006 | 0.008 | 0.238 | -0.017 | 0.0147 | 0.262 | -0.011 | 0.0211 | 0.594 |
| rs1451109 | 4 | 137071335 | G | A | 0.016 | 0.0017 | 1.36E-19 | 0.002 | 0.008 | 0.405 | -0.012 | 0.0150 | 0.429 | 0.008 | 0.0218 | 0.731 |
| rs57800857 | 4 | 140863365 | A | C | 0.016 | 0.0020 | 3.49E-15 | -0.030 | 0.008 | 0.000 | - | - | - | - | - | - |
| rs17019336 | 4 | 145333609 | T | A | 0.013 | 0.0019 | 2.45E-11 | 0.014 | 0.009 | 0.067 | 0.009 | 0.0166 | 0.593 | 0.011 | 0.0240 | 0.656 |
| rs3914628 | 4 | 147438019 | T | C | 0.017 | 0.0023 | 6.91E-13 | -0.014 | 0.010 | 0.095 | 0.009 | 0.0192 | 0.633 | -0.003 | 0.0280 | 0.909 |
| rs17276464 | 4 | 153028860 | T | C | 0.010 | 0.0016 | 4.81E-09 | -0.008 | 0.008 | 0.162 | 0.013 | 0.0145 | 0.358 | -0.015 | 0.0211 | 0.464 |
| rs13110266 | 4 | 162129844 | G | A | 0.012 | 0.0016 | 3.96E-14 | -0.006 | 0.008 | 0.207 | -0.013 | 0.0139 | 0.352 | -0.002 | 0.0200 | 0.918 |
| rs148636479 | 4 | 164359678 | A | T | 0.039 | 0.0062 | 4.81E-10 | 0.026 | 0.029 | 0.193 | 0.054 | 0.0548 | 0.328 | 0.071 | 0.0779 | 0.362 |
| rs1522569 | 4 | 171632637 | T | G | 0.014 | 0.0022 | 1.6E-10 | 0.006 | 0.011 | 0.271 | 0.008 | 0.0195 | 0.681 | -0.002 | 0.0278 | 0.955 |
| rs1437842 | 4 | 173597016 | G | A | 0.011 | 0.0017 | 8.49E-10 | 0.009 | 0.008 | 0.127 | 0.001 | 0.0140 | 0.949 | 0.013 | 0.0203 | 0.526 |
| rs6850421 | 4 | 180187034 | A | G | 0.011 | 0.0019 | 3.66E-09 | -0.019 | 0.008 | 0.010 | -0.014 | 0.0143 | 0.330 | 0.001 | 0.0210 | 0.964 |
| rs698147 | 5 | 3513485 | A | G | 0.012 | 0.0017 | 9.67E-12 | -0.005 | 0.008 | 0.258 | 0.010 | 0.0149 | 0.483 | 0.055 | 0.0223 | 0.013 |
| rs6890310 | 5 | 27193573 | G | A | 0.012 | 0.0019 | 3.29E-10 | -0.007 | 0.009 | 0.224 | 0.005 | 0.0160 | 0.754 | 0.001 | 0.0233 | 0.956 |
| rs6451675 | 5 | 43110855 | G | C | 0.014 | 0.0018 | 2.54E-14 | -0.031 | 0.008 | <0.001 | -0.033 | 0.0148 | 0.025 | 0.014 | 0.0218 | 0.535 |
| rs12189178 | 5 | 50914726 | T | C | 0.035 | 0.0046 | 4.3E-14 | -0.025 | 0.020 | 0.106 | 0.014 | 0.0369 | 0.715 | -0.107 | 0.0550 | 0.052 |
| rs7714712 | 5 | 60732552 | C | A | 0.011 | 0.0018 | 2.31E-10 | 0.005 | 0.008 | 0.274 | 0.018 | 0.0147 | 0.212 | 0.034 | 0.0210 | 0.106 |
| rs4700608 | 5 | 63026280 | C | T | 0.016 | 0.0017 | 4.32E-20 | -0.007 | 0.007 | 0.162 | -0.018 | 0.0135 | 0.186 | -0.002 | 0.0196 | 0.901 |
| rs12522567 | 5 | 64087023 | T | G | 0.012 | 0.0016 | 2.31E-13 | -0.002 | 0.008 | 0.423 | 0.005 | 0.0141 | 0.712 | -0.015 | 0.0204 | 0.473 |
| rs249612 | 5 | 66200783 | T | C | 0.015 | 0.0021 | 4.91E-13 | 0.014 | 0.009 | 0.054 | 0.012 | 0.0161 | 0.447 | -0.011 | 0.0230 | 0.622 |
| rs2112347 | 5 | 75015242 | T | G | 0.028 | 0.0017 | 1.17E-61 | -0.008 | 0.008 | 0.141 | -0.019 | 0.0143 | 0.187 | 0.007 | 0.0208 | 0.751 |
| rs10942267 | 5 | 80841914 | A | G | 0.015 | 0.0018 | 6.53E-16 | -0.001 | 0.008 | 0.451 | -0.008 | 0.0152 | 0.600 | -0.020 | 0.0216 | 0.363 |
| rs1501673 | 5 | 87963600 | A | G | 0.029 | 0.0025 | 2.73E-31 | -0.002 | 0.011 | 0.443 | -0.015 | 0.0207 | 0.464 | 0.040 | 0.0297 | 0.180 |
| rs4242244 | 5 | 92546237 | T | G | 0.011 | 0.0017 | 2E-10 | -0.015 | 0.008 | 0.026 | -0.015 | 0.0142 | 0.282 | -0.026 | 0.0205 | 0.210 |
| rs159032 | 5 | 94206202 | T | C | 0.012 | 0.0020 | 6.24E-10 | 0.013 | 0.009 | 0.078 | 0.006 | 0.0163 | 0.721 | -0.017 | 0.0234 | 0.475 |
| rs7713317 | 5 | 95716722 | G | A | 0.017 | 0.0018 | 1.96E-20 | 0.008 | 0.009 | 0.167 | 0.034 | 0.0156 | 0.030 | 0.043 | 0.0227 | 0.058 |
| rs2447832 | 5 | 103933473 | T | C | 0.011 | 0.0017 | 1.88E-10 | 0.011 | 0.008 | 0.067 | 0.012 | 0.0140 | 0.403 | 0.009 | 0.0202 | 0.652 |
| rs11739877 | 5 | 105876806 | T | C | 0.012 | 0.0018 | 4.01E-11 | 0.008 | 0.008 | 0.159 | 0.015 | 0.0149 | 0.316 | 0.024 | 0.0219 | 0.271 |
| rs288230 | 5 | 107422067 | T | C | 0.024 | 0.0023 | 4.67E-26 | -0.005 | 0.010 | 0.314 | -0.001 | 0.0183 | 0.946 | -0.001 | 0.0266 | 0.965 |
| rs459552 | 5 | 112176756 | T | A | 0.013 | 0.0019 | 8.36E-12 | 0.004 | 0.009 | 0.313 | - | - | - | - | - | - |
| rs1582931 | 5 | 122657199 | G | A | 0.013 | 0.0017 | 1.91E-13 | 0.011 | 0.008 | 0.070 | 0.004 | 0.0140 | 0.794 | 0.036 | 0.0202 | 0.074 |
| rs6864049 | 5 | 124330522 | G | A | 0.012 | 0.0016 | 1.48E-13 | -0.014 | 0.008 | 0.030 | - | - | - | - | - | - |
| rs1363695 | 5 | 130378027 | C | T | 0.013 | 0.0020 | 8.06E-10 | 0.004 | 0.009 | 0.349 | -0.015 | 0.0164 | 0.373 | -0.020 | 0.0240 | 0.403 |
| rs329124 | 5 | 133865452 | A | G | 0.014 | 0.0017 | 4.26E-15 | -0.033 | 0.008 | <0.001 | -0.036 | 0.0137 | 0.008 | -0.037 | 0.0199 | 0.060 |
| rs13174863 | 5 | 139080745 | G | A | 0.020 | 0.0023 | 1.94E-17 | -0.005 | 0.012 | 0.338 | 0.003 | 0.0211 | 0.875 | -0.039 | 0.0314 | 0.219 |
| rs13155259 | 5 | 144623437 | G | A | 0.011 | 0.0018 | 2.33E-10 | 0.003 | 0.008 | 0.345 | -0.004 | 0.0147 | 0.795 | -0.014 | 0.0213 | 0.523 |
| rs7734385 | 5 | 158460212 | G | A | 0.010 | 0.0016 | 6.08E-10 | -0.004 | 0.008 | 0.318 | -0.005 | 0.0140 | 0.720 | 0.006 | 0.0202 | 0.762 |
| rs2861089 | 5 | 164557954 | A | T | 0.011 | 0.0017 | 1.55E-09 | -0.003 | 0.008 | 0.357 | 0.013 | 0.0146 | 0.378 | -0.002 | 0.0212 | 0.909 |
| rs2053682 | 5 | 170599327 | A | C | 0.017 | 0.0018 | 2.59E-20 | 0.022 | 0.008 | 0.004 | 0.008 | 0.0151 | 0.603 | 0.027 | 0.0219 | 0.228 |
| rs6556301 | 5 | 176527577 | G | T | 0.011 | 0.0017 | 8.14E-11 | -0.006 | 0.008 | 0.215 | 0.000 | 0.0146 | 0.983 | 0.015 | 0.0211 | 0.483 |
| rs2228213 | 6 | 12124855 | G | A | 0.014 | 0.0017 | 5.5E-17 | 0.002 | 0.008 | 0.415 | 0.014 | 0.0144 | 0.326 | 0.006 | 0.0209 | 0.786 |
| rs11757278 | 6 | 13180454 | T | C | 0.013 | 0.0019 | 6.92E-13 | -0.006 | 0.008 | 0.218 | 0.003 | 0.0148 | 0.830 | -0.001 | 0.0216 | 0.973 |
| rs3806114 | 6 | 20482335 | G | A | 0.012 | 0.0018 | 1.46E-11 | -0.017 | 0.008 | 0.018 | -0.014 | 0.0151 | 0.360 | 0.011 | 0.0215 | 0.595 |
| rs6934973 | 6 | 21921026 | G | A | 0.012 | 0.0019 | 3.54E-10 | 0.002 | 0.008 | 0.419 | 0.013 | 0.0153 | 0.384 | -0.023 | 0.0221 | 0.306 |
| rs1355459 | 6 | 23871700 | A | G | 0.011 | 0.0018 | 9.56E-10 | 0.010 | 0.008 | 0.116 | 0.022 | 0.0148 | 0.140 | -0.001 | 0.0216 | 0.950 |
| rs2178899 | 6 | 31606756 | A | T | 0.024 | 0.0025 | 5.44E-23 | -0.005 | 0.012 | 0.350 | 0.008 | 0.0214 | 0.705 | -0.030 | 0.0298 | 0.315 |
| rs9277972 | 6 | 33276770 | T | A | 0.017 | 0.0022 | 5.46E-15 | -0.017 | 0.010 | 0.043 | 0.018 | 0.0177 | 0.315 | -0.016 | 0.0254 | 0.542 |
| rs6932930 | 6 | 34677103 | G | A | 0.026 | 0.0019 | 1.18E-40 | 0.034 | 0.009 | <0.001 | 0.053 | 0.0159 | 0.001 | 0.049 | 0.0231 | 0.034 |
| rs35679149 | 6 | 43604167 | A | G | 0.041 | 0.0060 | 6.89E-12 | -0.060 | 0.027 | 0.015 | -0.075 | 0.0515 | 0.143 | -0.041 | 0.0732 | 0.573 |
| rs857601 | 6 | 44805214 | T | G | 0.011 | 0.0019 | 3.77E-09 | 0.003 | 0.008 | 0.382 | -0.002 | 0.0152 | 0.897 | 0.024 | 0.0220 | 0.273 |
| rs2206277 | 6 | 50798526 | T | C | 0.041 | 0.0021 | 1.82E-83 | -0.006 | 0.010 | 0.276 | -0.003 | 0.0175 | 0.887 | -0.011 | 0.0255 | 0.660 |
| rs816367 | 6 | 53995542 | G | C | 0.011 | 0.0017 | 1.02E-09 | -0.003 | 0.008 | 0.373 | -0.010 | 0.0145 | 0.477 | -0.031 | 0.0210 | 0.137 |
| rs2622274 | 6 | 64240516 | G | T | 0.011 | 0.0017 | 3.23E-10 | 0.002 | 0.008 | 0.381 | 0.014 | 0.0149 | 0.367 | 0.002 | 0.0222 | 0.942 |
| rs6916553 | 6 | 70146918 | G | A | 0.012 | 0.0020 | 2.64E-09 | 0.010 | 0.009 | 0.131 | 0.022 | 0.0164 | 0.173 | 0.011 | 0.0236 | 0.635 |
| rs7769594 | 6 | 83447813 | T | C | 0.016 | 0.0023 | 2.13E-12 | 0.001 | 0.011 | 0.459 | -0.022 | 0.0194 | 0.264 | -0.009 | 0.0279 | 0.740 |
| rs16882001 | 6 | 90322237 | G | A | 0.023 | 0.0038 | 3.26E-09 | -0.011 | 0.018 | 0.267 | -0.020 | 0.0341 | 0.555 | 0.017 | 0.0471 | 0.726 |
| rs9320823 | 6 | 98429337 | C | T | 0.017 | 0.0017 | 2.07E-21 | -0.002 | 0.008 | 0.424 | 0.011 | 0.0143 | 0.428 | -0.012 | 0.0205 | 0.575 |
| rs12209887 | 6 | 101156806 | A | G | 0.011 | 0.0017 | 1.78E-11 | 0.009 | 0.008 | 0.126 | 0.001 | 0.0137 | 0.936 | -0.001 | 0.0199 | 0.972 |
| rs1417665 | 6 | 104816944 | T | C | 0.019 | 0.0022 | 5.49E-18 | 0.000 | 0.010 | 0.492 | 0.002 | 0.0181 | 0.922 | 0.003 | 0.0267 | 0.914 |
| rs12206094 | 6 | 108906200 | C | T | 0.014 | 0.0018 | 1.93E-15 | 0.015 | 0.009 | 0.041 | 0.036 | 0.0160 | 0.025 | 0.029 | 0.0230 | 0.208 |
| rs2357760 | 6 | 120213880 | A | G | 0.014 | 0.0017 | 2.11E-16 | -0.001 | 0.008 | 0.440 | -0.018 | 0.0145 | 0.206 | -0.021 | 0.0211 | 0.320 |
| rs1871329 | 6 | 124911534 | G | A | 0.013 | 0.0020 | 1.03E-10 | 0.010 | 0.009 | 0.129 | 0.024 | 0.0168 | 0.156 | 0.003 | 0.0243 | 0.906 |
| rs1269175 | 6 | 126040435 | A | G | 0.011 | 0.0017 | 8.09E-10 | -0.018 | 0.008 | 0.010 | -0.018 | 0.0140 | 0.190 | 0.009 | 0.0203 | 0.655 |
| rs7740107 | 6 | 130374461 | T | A | 0.012 | 0.0019 | 2.58E-09 | -0.038 | 0.009 | 0.000 | -0.030 | 0.0158 | 0.056 | -0.094 | 0.0230 | 0.000 |
| rs2246012 | 6 | 131898208 | C | T | 0.016 | 0.0022 | 1.15E-13 | -0.023 | 0.010 | 0.012 | - | - | - | - | - | - |
| rs9376609 | 6 | 141671732 | A | G | 0.011 | 0.0018 | 1.66E-10 | -0.011 | 0.008 | 0.076 | -0.023 | 0.0144 | 0.104 | -0.004 | 0.0206 | 0.850 |
| rs765875 | 6 | 143185683 | C | T | 0.013 | 0.0017 | 1.08E-14 | -0.011 | 0.008 | 0.085 | -0.015 | 0.0144 | 0.303 | 0.006 | 0.0207 | 0.781 |
| rs487152 | 6 | 160774486 | A | C | 0.011 | 0.0016 | 2.98E-11 | -0.079 | 0.007 | <0.001 | -0.067 | 0.0135 | 0.000 | -0.125 | 0.0195 | 0.000 |
| rs13191362 | 6 | 163033350 | A | G | 0.024 | 0.0025 | 4.08E-21 | 0.009 | 0.012 | 0.220 | - | - | - | - | - | - |
| rs4721089 | 7 | 1872921 | T | C | 0.017 | 0.0023 | 5.6E-13 | 0.011 | 0.009 | 0.125 | 0.012 | 0.0167 | 0.465 | 0.050 | 0.0247 | 0.041 |
| rs6463489 | 7 | 5542513 | T | C | 0.017 | 0.0026 | 2.5E-10 | 0.023 | 0.013 | 0.042 | -0.001 | 0.0247 | 0.964 | 0.068 | 0.0351 | 0.053 |
| rs6968554 | 7 | 17287106 | G | A | 0.011 | 0.0017 | 2.34E-10 | -0.006 | 0.008 | 0.217 | -0.004 | 0.0144 | 0.758 | -0.014 | 0.0207 | 0.512 |
| rs4721823 | 7 | 19785888 | G | C | 0.014 | 0.0024 | 4.52E-09 | -0.012 | 0.011 | 0.130 | 0.021 | 0.0202 | 0.311 | 0.002 | 0.0289 | 0.940 |
| rs4307239 | 7 | 24354300 | G | A | 0.012 | 0.0017 | 1.47E-11 | 0.011 | 0.008 | 0.071 | 0.000 | 0.0141 | 0.989 | 0.005 | 0.0205 | 0.815 |
| rs11971098 | 7 | 26699061 | G | A | 0.020 | 0.0030 | 8.08E-11 | 0.045 | 0.014 | <0.001 | 0.027 | 0.0249 | 0.271 | 0.076 | 0.0348 | 0.029 |
| rs1635853 | 7 | 28189549 | G | T | 0.010 | 0.0016 | 2.99E-09 | 0.005 | 0.008 | 0.268 | - | - | - | - | - | - |
| rs215614 | 7 | 32347335 | G | A | 0.014 | 0.0018 | 2.77E-16 | -0.001 | 0.008 | 0.465 | 0.016 | 0.0147 | 0.289 | 0.010 | 0.0213 | 0.649 |
| rs2237403 | 7 | 39448936 | C | T | 0.013 | 0.0018 | 1.22E-12 | -0.009 | 0.008 | 0.133 | -0.025 | 0.0146 | 0.092 | -0.008 | 0.0212 | 0.720 |
| rs6962280 | 7 | 44788657 | G | A | 0.014 | 0.0019 | 3.37E-13 | -0.020 | 0.008 | 0.005 | -0.020 | 0.0143 | 0.154 | -0.037 | 0.0208 | 0.073 |
| rs10499694 | 7 | 50614173 | A | G | 0.013 | 0.0016 | 1.29E-15 | -0.006 | 0.008 | 0.234 | -0.003 | 0.0142 | 0.821 | 0.003 | 0.0205 | 0.875 |
| rs1035010 | 7 | 69598328 | T | C | 0.014 | 0.0020 | 2.01E-12 | 0.017 | 0.009 | 0.022 | 0.021 | 0.0158 | 0.185 | 0.024 | 0.0230 | 0.289 |
| rs10950289 | 7 | 71429308 | A | G | 0.018 | 0.0026 | 1.44E-11 | 0.004 | 0.011 | 0.369 | 0.005 | 0.0191 | 0.801 | -0.011 | 0.0284 | 0.698 |
| rs6954694 | 7 | 76632098 | G | A | 0.024 | 0.0026 | 1.09E-20 | 0.003 | 0.011 | 0.378 | -0.010 | 0.0199 | 0.603 | -0.010 | 0.0295 | 0.746 |
| rs1852006 | 7 | 77829768 | G | A | 0.015 | 0.0018 | 6.71E-18 | 0.009 | 0.008 | 0.128 | 0.006 | 0.0146 | 0.665 | 0.018 | 0.0211 | 0.386 |
| rs7777084 | 7 | 93089243 | A | G | 0.013 | 0.0017 | 1.58E-14 | -0.005 | 0.008 | 0.274 | -0.022 | 0.0137 | 0.110 | -0.004 | 0.0197 | 0.858 |
| rs11761528 | 7 | 99118801 | C | T | 0.023 | 0.0030 | 6.08E-14 | -0.010 | 0.013 | 0.219 | -0.005 | 0.0236 | 0.835 | -0.054 | 0.0344 | 0.118 |
| rs2299383 | 7 | 103418846 | T | C | 0.016 | 0.0016 | 6.89E-23 | -0.007 | 0.008 | 0.171 | -0.016 | 0.0143 | 0.275 | 0.003 | 0.0206 | 0.904 |
| rs1721447 | 7 | 109214139 | G | T | 0.010 | 0.0017 | 3.79E-09 | -0.003 | 0.008 | 0.344 | -0.010 | 0.0140 | 0.498 | 0.020 | 0.0203 | 0.320 |
| rs2396625 | 7 | 113028634 | T | A | 0.018 | 0.0017 | 2.81E-24 | -0.008 | 0.008 | 0.146 | - | - | - | -0.008 | 0.0203 | 0.702 |
| rs1899689 | 7 | 121964349 | T | C | 0.012 | 0.0017 | 4.2E-13 | -0.011 | 0.008 | 0.093 | -0.021 | 0.0148 | 0.166 | 0.030 | 0.0216 | 0.160 |
| rs7802342 | 7 | 137435925 | G | T | 0.012 | 0.0019 | 6.23E-11 | -0.002 | 0.009 | 0.421 | 0.004 | 0.0166 | 0.807 | 0.044 | 0.0245 | 0.069 |
| rs11525873 | 7 | 138817193 | T | C | 0.023 | 0.0032 | 2.98E-13 | -0.025 | 0.014 | 0.033 | -0.004 | 0.0248 | 0.870 | -0.064 | 0.0361 | 0.075 |
| rs62491456 | 7 | 139885277 | T | G | 0.020 | 0.0033 | 1.29E-09 | 0.021 | 0.014 | 0.066 | -0.011 | 0.0260 | 0.675 | 0.005 | 0.0368 | 0.897 |
| rs2907948 | 7 | 150638484 | G | A | 0.015 | 0.0019 | 1.95E-14 | 0.009 | 0.009 | 0.161 | 0.010 | 0.0165 | 0.561 | 0.042 | 0.0235 | 0.071 |
| rs1658820 | 8 | 4288577 | T | G | 0.013 | 0.0020 | 3.77E-11 | 0.001 | 0.009 | 0.478 | -0.009 | 0.0165 | 0.602 | 0.005 | 0.0239 | 0.852 |
| rs4841504 | 8 | 11024663 | C | A | 0.017 | 0.0016 | 5.41E-24 | 0.017 | 0.008 | 0.012 | 0.036 | 0.0141 | 0.011 | 0.031 | 0.0205 | 0.132 |
| rs4123853 | 8 | 14091025 | T | C | 0.014 | 0.0017 | 1.06E-15 | -0.005 | 0.008 | 0.267 | -0.026 | 0.0141 | 0.070 | -0.021 | 0.0203 | 0.293 |
| rs354508 | 8 | 15535226 | C | T | 0.015 | 0.0023 | 2.69E-10 | -0.017 | 0.011 | 0.058 | -0.023 | 0.0193 | 0.231 | -0.029 | 0.0274 | 0.290 |
| rs10101364 | 8 | 20634888 | T | C | 0.012 | 0.0018 | 5.61E-11 | 0.002 | 0.008 | 0.402 | -0.004 | 0.0148 | 0.781 | 0.021 | 0.0215 | 0.324 |
| rs11781222 | 8 | 23389571 | T | C | 0.017 | 0.0024 | 3.25E-12 | -0.010 | 0.011 | 0.179 | - | - | - | - | - | - |
| rs1982441 | 8 | 28021769 | T | G | 0.017 | 0.0025 | 2.17E-11 | 0.010 | 0.011 | 0.182 | 0.018 | 0.0198 | 0.358 | -0.021 | 0.0296 | 0.482 |
| rs10954772 | 8 | 30863938 | T | C | 0.016 | 0.0021 | 5.33E-14 | -0.003 | 0.008 | 0.350 | 0.008 | 0.0154 | 0.611 | -0.005 | 0.0221 | 0.829 |
| rs7826312 | 8 | 32400115 | C | T | 0.011 | 0.0016 | 3.23E-11 | -0.010 | 0.008 | 0.095 | - | - | - | - | - | - |
| rs4739570 | 8 | 34324581 | A | G | 0.011 | 0.0018 | 1.05E-09 | 0.006 | 0.008 | 0.229 | 0.005 | 0.0146 | 0.741 | 0.011 | 0.0211 | 0.606 |
| rs36061954 | 8 | 38329650 | T | C | 0.012 | 0.0019 | 1.05E-09 | -0.021 | 0.008 | 0.003 | -0.019 | 0.0145 | 0.198 | -0.031 | 0.0209 | 0.133 |
| rs12681792 | 8 | 62054463 | A | C | 0.015 | 0.0021 | 2.88E-12 | -0.005 | 0.010 | 0.298 | 0.012 | 0.0180 | 0.510 | -0.004 | 0.0266 | 0.888 |
| rs10092723 | 8 | 67194710 | C | A | 0.013 | 0.0020 | 1.17E-11 | -0.005 | 0.009 | 0.277 | 0.004 | 0.0162 | 0.820 | -0.010 | 0.0236 | 0.684 |
| rs1431659 | 8 | 73439070 | A | G | 0.019 | 0.0019 | 2.33E-23 | -0.005 | 0.009 | 0.272 | 0.006 | 0.0164 | 0.698 | 0.005 | 0.0235 | 0.848 |
| rs2170382 | 8 | 74689288 | T | C | 0.018 | 0.0027 | 4.26E-11 | -0.007 | 0.012 | 0.276 | -0.008 | 0.0226 | 0.740 | -0.012 | 0.0326 | 0.715 |
| rs17405819 | 8 | 76806584 | T | C | 0.021 | 0.0018 | 6.04E-33 | 0.000 | 0.008 | 0.485 | -0.005 | 0.0148 | 0.733 | 0.033 | 0.0214 | 0.129 |
| rs16907751 | 8 | 81375457 | C | T | 0.019 | 0.0029 | 1.63E-11 | -0.013 | 0.013 | 0.144 | - | - | - | - | - | - |
| rs2196618 | 8 | 85089437 | G | A | 0.014 | 0.0019 | 1.49E-12 | -0.002 | 0.009 | 0.394 | 0.010 | 0.0164 | 0.563 | 0.000 | 0.0240 | 0.996 |
| rs12546331 | 8 | 87505968 | T | C | 0.011 | 0.0017 | 2.09E-10 | -0.003 | 0.008 | 0.342 | 0.016 | 0.0140 | 0.253 | 0.006 | 0.0202 | 0.755 |
| rs1601817 | 8 | 89462321 | C | A | 0.011 | 0.0018 | 2.38E-09 | 0.001 | 0.008 | 0.476 | 0.001 | 0.0152 | 0.949 | 0.011 | 0.0220 | 0.619 |
| rs12680842 | 8 | 95582606 | A | G | 0.014 | 0.0017 | 3.41E-16 | 0.005 | 0.008 | 0.252 | - | - | - | - | - | - |
| rs1383592 | 8 | 106430676 | A | G | 0.012 | 0.0021 | 4.92E-09 | -0.022 | 0.009 | 0.009 | -0.015 | 0.0170 | 0.373 | -0.009 | 0.0249 | 0.723 |
| rs3808477 | 8 | 116670347 | C | T | 0.018 | 0.0019 | 8.73E-22 | 0.021 | 0.008 | 0.006 | 0.016 | 0.0153 | 0.307 | 0.012 | 0.0222 | 0.580 |
| rs72673947 | 8 | 118884379 | G | A | 0.022 | 0.0031 | 4.11E-13 | -0.036 | 0.013 | 0.002 | -0.041 | 0.0233 | 0.080 | -0.037 | 0.0344 | 0.285 |
| rs2954021 | 8 | 126482077 | G | A | 0.011 | 0.0017 | 2.41E-10 | 0.009 | 0.008 | 0.126 | 0.003 | 0.0140 | 0.807 | -0.031 | 0.0202 | 0.129 |
| rs11997238 | 8 | 132871608 | G | A | 0.019 | 0.0030 | 5.53E-10 | 0.005 | 0.014 | 0.351 | 0.020 | 0.0256 | 0.445 | 0.034 | 0.0358 | 0.343 |
| rs16906845 | 8 | 138215228 | G | A | 0.024 | 0.0036 | 1.32E-11 | -0.032 | 0.016 | 0.020 | -0.033 | 0.0292 | 0.253 | -0.083 | 0.0435 | 0.058 |
| rs10099330 | 8 | 143383694 | G | A | 0.012 | 0.0017 | 3.22E-12 | 0.002 | 0.008 | 0.405 | -0.020 | 0.0137 | 0.137 | -0.012 | 0.0199 | 0.539 |
| rs7037266 | 9 | 6942940 | C | A | 0.011 | 0.0018 | 1.24E-09 | -0.003 | 0.008 | 0.346 | -0.013 | 0.0143 | 0.382 | 0.022 | 0.0208 | 0.299 |
| rs12336441 | 9 | 11619764 | A | T | 0.013 | 0.0017 | 4.68E-13 | -0.022 | 0.008 | 0.002 | -0.008 | 0.0144 | 0.603 | -0.036 | 0.0208 | 0.085 |
| rs4740619 | 9 | 15634326 | T | C | 0.019 | 0.0016 | 3.15E-31 | -0.007 | 0.008 | 0.162 | -0.001 | 0.0136 | 0.916 | -0.014 | 0.0198 | 0.492 |
| rs1411431 | 9 | 16728721 | A | C | 0.023 | 0.0026 | 1.46E-18 | 0.001 | 0.011 | 0.482 | 0.022 | 0.0199 | 0.278 | -0.050 | 0.0294 | 0.091 |
| rs10965780 | 9 | 23341715 | G | C | 0.011 | 0.0017 | 8.56E-10 | 0.002 | 0.008 | 0.425 | - | - | - | - | - | - |
| rs2183824 | 9 | 28412078 | T | C | 0.023 | 0.0017 | 2.29E-41 | 0.000 | 0.008 | 0.480 | 0.016 | 0.0149 | 0.292 | 0.040 | 0.0215 | 0.066 |
| rs12238336 | 9 | 29716655 | C | T | 0.012 | 0.0017 | 3.76E-12 | 0.006 | 0.008 | 0.200 | 0.006 | 0.0139 | 0.680 | 0.038 | 0.0202 | 0.058 |
| rs17720922 | 9 | 31030917 | T | C | 0.013 | 0.0022 | 4.18E-09 | 0.012 | 0.010 | 0.111 | 0.016 | 0.0175 | 0.369 | 0.021 | 0.0256 | 0.414 |
| rs6476617 | 9 | 37200103 | G | A | 0.015 | 0.0017 | 1.53E-17 | -0.003 | 0.008 | 0.338 | 0.014 | 0.0145 | 0.354 | -0.015 | 0.0208 | 0.473 |
| rs2134858 | 9 | 73837155 | C | T | 0.012 | 0.0017 | 5.87E-12 | -0.011 | 0.007 | 0.070 | -0.020 | 0.0136 | 0.137 | -0.014 | 0.0196 | 0.478 |
| rs1634350 | 9 | 81334684 | A | C | 0.011 | 0.0017 | 3.19E-11 | 0.007 | 0.008 | 0.202 | 0.017 | 0.0145 | 0.245 | -0.015 | 0.0213 | 0.472 |
| rs2777768 | 9 | 84186734 | A | G | 0.012 | 0.0019 | 6.38E-10 | 0.005 | 0.009 | 0.302 | 0.012 | 0.0160 | 0.466 | -0.008 | 0.0231 | 0.732 |
| rs1187352 | 9 | 87293457 | C | T | 0.012 | 0.0018 | 2.61E-11 | 0.003 | 0.008 | 0.349 | -0.011 | 0.0147 | 0.441 | 0.032 | 0.0212 | 0.132 |
| rs6559921 | 9 | 88872039 | A | T | 0.011 | 0.0020 | 2.86E-08 | 0.005 | 0.009 | 0.297 | 0.012 | 0.0164 | 0.481 | -0.002 | 0.0238 | 0.947 |
| rs7357754 | 9 | 92207308 | G | A | 0.012 | 0.0017 | 1.81E-12 | -0.011 | 0.008 | 0.082 | -0.002 | 0.0139 | 0.904 | -0.010 | 0.0200 | 0.626 |
| rs3811125 | 9 | 94187247 | C | T | 0.015 | 0.0021 | 9.42E-13 | -0.005 | 0.009 | 0.302 | -0.012 | 0.0162 | 0.465 | -0.043 | 0.0230 | 0.060 |
| rs10761247 | 9 | 96403367 | G | A | 0.011 | 0.0017 | 4.53E-11 | 0.013 | 0.008 | 0.043 | 0.005 | 0.0141 | 0.730 | 0.015 | 0.0203 | 0.465 |
| rs12551906 | 9 | 102119090 | G | A | 0.012 | 0.0018 | 7.72E-11 | -0.002 | 0.009 | 0.417 | 0.013 | 0.0158 | 0.422 | 0.007 | 0.0231 | 0.753 |
| rs7024334 | 9 | 109072075 | T | G | 0.014 | 0.0020 | 4.71E-12 | -0.009 | 0.009 | 0.170 | 0.012 | 0.0163 | 0.480 | 0.011 | 0.0234 | 0.649 |
| rs6477694 | 9 | 111932342 | C | T | 0.013 | 0.0017 | 6.57E-14 | -0.005 | 0.008 | 0.271 | -0.005 | 0.0141 | 0.749 | -0.011 | 0.0205 | 0.588 |
| rs1928295 | 9 | 120378483 | T | C | 0.013 | 0.0016 | 2.23E-16 | 0.008 | 0.008 | 0.159 | 0.031 | 0.0140 | 0.025 | 0.011 | 0.0203 | 0.584 |
| rs10984756 | 9 | 122651784 | G | C | 0.018 | 0.0028 | 4.37E-10 | -0.005 | 0.014 | 0.361 | 0.007 | 0.0247 | 0.768 | -0.020 | 0.0361 | 0.588 |
| rs7871866 | 9 | 131027982 | C | G | 0.018 | 0.0024 | 7.56E-14 | -0.001 | 0.011 | 0.474 | -0.003 | 0.0197 | 0.864 | 0.009 | 0.0288 | 0.761 |
| rs3739514 | 9 | 133783025 | A | G | 0.013 | 0.0020 | 6.64E-11 | -0.002 | 0.008 | 0.406 | 0.016 | 0.0151 | 0.296 | -0.002 | 0.0222 | 0.924 |
| rs10858334 | 9 | 137989785 | G | C | 0.015 | 0.0025 | 4.91E-09 | -0.008 | 0.013 | 0.269 | 0.008 | 0.0226 | 0.711 | -0.008 | 0.0325 | 0.801 |
| rs6602411 | 10 | 10264200 | T | C | 0.015 | 0.0022 | 5.98E-11 | 0.002 | 0.010 | 0.431 | -0.002 | 0.0179 | 0.928 | -0.052 | 0.0265 | 0.049 |
| rs7893571 | 10 | 16750129 | T | G | 0.013 | 0.0018 | 5.83E-12 | -0.009 | 0.008 | 0.136 | -0.014 | 0.0151 | 0.346 | -0.002 | 0.0220 | 0.946 |
| rs1277733 | 10 | 18562538 | T | C | 0.012 | 0.0020 | 4.3E-09 | 0.005 | 0.009 | 0.293 | 0.020 | 0.0169 | 0.241 | 0.028 | 0.0246 | 0.253 |
| rs7084454 | 10 | 21821274 | A | G | 0.020 | 0.0018 | 4.51E-27 | -0.006 | 0.008 | 0.219 | 0.000 | 0.0146 | 0.988 | 0.004 | 0.0209 | 0.839 |
| rs12765914 | 10 | 34013507 | T | C | 0.023 | 0.0031 | 1.96E-13 | -0.014 | 0.013 | 0.151 | -0.013 | 0.0240 | 0.596 | -0.044 | 0.0356 | 0.221 |
| rs10823893 | 10 | 53677313 | A | G | 0.012 | 0.0019 | 1.55E-10 | 0.007 | 0.008 | 0.175 | -0.008 | 0.0143 | 0.598 | 0.001 | 0.0208 | 0.949 |
| rs7070670 | 10 | 61842645 | C | T | 0.013 | 0.0021 | 7.14E-10 | -0.003 | 0.008 | 0.378 | -0.002 | 0.0154 | 0.897 | -0.039 | 0.0222 | 0.081 |
| rs10761785 | 10 | 65318766 | G | T | 0.013 | 0.0016 | 3.47E-16 | 0.000 | 0.008 | 0.484 | - | - | - | - | - | - |
| rs2933451 | 10 | 66626611 | C | A | 0.013 | 0.0020 | 1.98E-10 | -0.010 | 0.008 | 0.108 | -0.024 | 0.0147 | 0.100 | -0.024 | 0.0213 | 0.256 |
| rs12098284 | 10 | 76047464 | T | C | 0.018 | 0.0026 | 9.87E-13 | 0.013 | 0.012 | 0.145 | -0.004 | 0.0222 | 0.870 | 0.034 | 0.0312 | 0.277 |
| rs11001963 | 10 | 78760959 | T | C | 0.011 | 0.0017 | 8.54E-11 | 0.000 | 0.008 | 0.495 | -0.016 | 0.0143 | 0.276 | -0.001 | 0.0207 | 0.959 |
| rs7899106 | 10 | 87410904 | G | A | 0.033 | 0.0037 | 1.72E-18 | 0.003 | 0.018 | 0.438 | -0.017 | 0.0331 | 0.614 | -0.051 | 0.0483 | 0.295 |
| rs2450448 | 10 | 93062111 | A | T | 0.013 | 0.0021 | 3.93E-10 | 0.013 | 0.008 | 0.055 | 0.026 | 0.0151 | 0.091 | 0.014 | 0.0224 | 0.538 |
| rs2439823 | 10 | 99778226 | G | A | 0.017 | 0.0017 | 6.51E-22 | -0.007 | 0.008 | 0.178 | -0.012 | 0.0145 | 0.420 | -0.031 | 0.0210 | 0.145 |
| rs41310284 | 10 | 102447647 | C | A | 0.029 | 0.0032 | 3.98E-19 | -0.009 | 0.014 | 0.264 | 0.032 | 0.0255 | 0.203 | 0.039 | 0.0362 | 0.281 |
| rs4290163 | 10 | 104610926 | T | G | 0.013 | 0.0017 | 3.5E-15 | -0.029 | 0.008 | <0.001 | -0.025 | 0.0140 | 0.073 | -0.052 | 0.0203 | 0.010 |
| rs7903146 | 10 | 114758349 | C | T | 0.018 | 0.0018 | 1.67E-23 | -0.009 | 0.008 | 0.145 | -0.007 | 0.0155 | 0.647 | -0.070 | 0.0220 | 0.002 |
| rs10886017 | 10 | 118672531 | A | C | 0.015 | 0.0019 | 4.47E-15 | -0.015 | 0.009 | 0.043 | - | - | - | - | - | - |
| rs845084 | 10 | 125220036 | A | G | 0.014 | 0.0019 | 3.22E-12 | 0.008 | 0.009 | 0.179 | -0.009 | 0.0160 | 0.595 | -0.003 | 0.0232 | 0.882 |
| rs17636031 | 10 | 126594078 | C | T | 0.015 | 0.0018 | 3.87E-17 | 0.049 | 0.008 | 0.000 | - | - | - | - | - | - |
| rs4880341 | 10 | 133992689 | C | T | 0.013 | 0.0017 | 3.06E-14 | -0.006 | 0.008 | 0.213 | 0.005 | 0.0140 | 0.723 | -0.055 | 0.0203 | 0.007 |
| rs10840606 | 11 | 2234690 | G | A | 0.015 | 0.0023 | 2.21E-10 | 0.155 | 0.010 | <0.001 | 0.104 | 0.0174 | 0.000 | 0.293 | 0.0246 | 0.000 |
| rs3750944 | 11 | 6239344 | G | A | 0.010 | 0.0018 | 4.74E-09 | 0.005 | 0.009 | 0.296 | 0.000 | 0.0158 | 0.987 | 0.013 | 0.0234 | 0.577 |
| rs4256980 | 11 | 8673939 | G | C | 0.019 | 0.0017 | 8.63E-29 | 0.011 | 0.008 | 0.084 | - | - | - | - | - | - |
| rs1982350 | 11 | 13350131 | G | A | 0.016 | 0.0018 | 1.98E-18 | 0.005 | 0.008 | 0.269 | -0.009 | 0.0143 | 0.549 | 0.005 | 0.0207 | 0.822 |
| rs5215 | 11 | 17408630 | T | C | 0.011 | 0.0017 | 1.22E-11 | -0.003 | 0.008 | 0.358 | 0.009 | 0.0142 | 0.510 | -0.021 | 0.0206 | 0.317 |
| rs6265 | 11 | 27679916 | C | T | 0.041 | 0.0021 | 7.4E-89 | -0.006 | 0.010 | 0.273 | -0.012 | 0.0177 | 0.493 | -0.007 | 0.0254 | 0.770 |
| rs2452141 | 11 | 29230734 | T | A | 0.011 | 0.0017 | 7.22E-11 | 0.014 | 0.008 | 0.044 | -0.011 | 0.0144 | 0.469 | -0.014 | 0.0209 | 0.518 |
| rs2065418 | 11 | 30422068 | T | G | 0.014 | 0.0018 | 6.07E-15 | -0.007 | 0.008 | 0.205 | -0.028 | 0.0148 | 0.059 | -0.010 | 0.0214 | 0.649 |
| rs223058 | 11 | 32125855 | G | A | 0.011 | 0.0018 | 3.29E-10 | 0.006 | 0.008 | 0.251 | 0.012 | 0.0150 | 0.428 | 0.011 | 0.0215 | 0.614 |
| rs2862996 | 11 | 43653833 | G | T | 0.022 | 0.0017 | 3.6E-35 | -0.001 | 0.008 | 0.431 | 0.003 | 0.0149 | 0.830 | -0.009 | 0.0214 | 0.673 |
| rs11038428 | 11 | 45404318 | G | T | 0.013 | 0.0018 | 1.8E-13 | -0.010 | 0.009 | 0.118 | -0.024 | 0.0159 | 0.123 | -0.021 | 0.0232 | 0.376 |
| rs7124681 | 11 | 47529947 | A | C | 0.026 | 0.0016 | 3.96E-55 | -0.020 | 0.008 | 0.004 | -0.002 | 0.0144 | 0.878 | -0.021 | 0.0208 | 0.323 |
| rs6591407 | 11 | 56914157 | C | A | 0.012 | 0.0021 | 3.58E-09 | -0.011 | 0.010 | 0.126 | -0.014 | 0.0178 | 0.446 | 0.027 | 0.0260 | 0.293 |
| rs7947143 | 11 | 64090422 | G | A | 0.018 | 0.0023 | 8.13E-14 | -0.019 | 0.011 | 0.034 | -0.016 | 0.0192 | 0.416 | 0.036 | 0.0276 | 0.191 |
| rs10896012 | 11 | 65278461 | C | T | 0.016 | 0.0021 | 1.02E-14 | 0.023 | 0.010 | 0.010 | - | - | - | - | - | - |
| rs592483 | 11 | 69445173 | C | T | 0.014 | 0.0017 | 1.53E-16 | -0.006 | 0.008 | 0.225 | -0.013 | 0.0142 | 0.375 | -0.022 | 0.0204 | 0.288 |
| rs7123876 | 11 | 72444583 | C | T | 0.012 | 0.0019 | 2.92E-10 | -0.005 | 0.009 | 0.277 | -0.005 | 0.0160 | 0.760 | -0.018 | 0.0235 | 0.443 |
| rs12282785 | 11 | 76476030 | C | A | 0.016 | 0.0023 | 1.43E-11 | -0.014 | 0.009 | 0.060 | -0.022 | 0.0164 | 0.174 | -0.028 | 0.0238 | 0.237 |
| rs349088 | 11 | 84814393 | C | A | 0.013 | 0.0017 | 3.5E-14 | -0.011 | 0.008 | 0.069 | -0.016 | 0.0137 | 0.242 | -0.047 | 0.0198 | 0.017 |
| rs61903695 | 11 | 89922417 | G | A | 0.015 | 0.0022 | 4.54E-11 | 0.007 | 0.009 | 0.218 | 0.021 | 0.0164 | 0.196 | 0.002 | 0.0237 | 0.933 |
| rs2605603 | 11 | 93221105 | G | A | 0.010 | 0.0016 | 2.04E-10 | -0.006 | 0.008 | 0.200 | -0.014 | 0.0138 | 0.328 | -0.035 | 0.0200 | 0.081 |
| rs2155645 | 11 | 112912947 | C | T | 0.011 | 0.0019 | 8.17E-10 | -0.005 | 0.009 | 0.290 | -0.005 | 0.0155 | 0.737 | 0.000 | 0.0226 | 0.995 |
| rs12286929 | 11 | 115022404 | G | A | 0.018 | 0.0016 | 1.93E-27 | -0.018 | 0.008 | 0.012 | -0.001 | 0.0142 | 0.949 | -0.030 | 0.0207 | 0.151 |
| rs76942203 | 11 | 116973247 | A | G | 0.026 | 0.0041 | 9.08E-11 | 0.004 | 0.016 | 0.397 | 0.015 | 0.0296 | 0.623 | -0.020 | 0.0437 | 0.654 |
| rs3825061 | 11 | 118944675 | T | C | 0.014 | 0.0017 | 6.15E-16 | 0.009 | 0.008 | 0.135 | 0.016 | 0.0144 | 0.275 | 0.037 | 0.0206 | 0.073 |
| rs11218510 | 11 | 121922587 | G | A | 0.014 | 0.0020 | 6.79E-13 | 0.000 | 0.008 | 0.495 | 0.003 | 0.0146 | 0.831 | -0.032 | 0.0212 | 0.131 |
| rs7944782 | 11 | 130795698 | G | T | 0.014 | 0.0017 | 3.61E-17 | -0.015 | 0.008 | 0.026 | -0.003 | 0.0140 | 0.822 | -0.007 | 0.0202 | 0.738 |
| rs12222235 | 11 | 131984330 | C | T | 0.013 | 0.0018 | 1.78E-12 | -0.013 | 0.008 | 0.061 | -0.042 | 0.0147 | 0.004 | -0.042 | 0.0212 | 0.047 |
| rs12364470 | 11 | 134601012 | G | T | 0.019 | 0.0022 | 2.18E-17 | 0.007 | 0.011 | 0.264 | 0.007 | 0.0203 | 0.732 | 0.021 | 0.0305 | 0.500 |
| rs11611246 | 12 | 939480 | T | G | 0.022 | 0.0020 | 2.04E-28 | 0.009 | 0.009 | 0.169 | - | - | - | - | - | - |
| rs2470397 | 12 | 2106116 | C | T | 0.013 | 0.0022 | 2.8E-09 | 0.008 | 0.010 | 0.208 | - | - | - | - | - | - |
| rs12422552 | 12 | 14413931 | G | C | 0.013 | 0.0019 | 1.78E-11 | 0.025 | 0.009 | 0.002 | - | - | - | - | - | - |
| rs10744146 | 12 | 17212881 | G | A | 0.011 | 0.0017 | 2.54E-11 | -0.012 | 0.008 | 0.052 | -0.024 | 0.0139 | 0.079 | -0.014 | 0.0201 | 0.494 |
| rs7976757 | 12 | 19207948 | C | T | 0.015 | 0.0022 | 2.73E-11 | -0.005 | 0.010 | 0.321 | - | - | - | - | - | - |
| rs11047132 | 12 | 24008435 | G | T | 0.023 | 0.0031 | 1.59E-13 | -0.012 | 0.013 | 0.194 | - | - | - | - | - | - |
| rs11170468 | 12 | 39430048 | A | C | 0.013 | 0.0019 | 1.12E-11 | -0.002 | 0.009 | 0.427 | - | - | - | - | - | - |
| rs11181001 | 12 | 41948196 | A | G | 0.014 | 0.0016 | 1.17E-16 | -0.001 | 0.008 | 0.458 | - | - | - | - | - | - |
| rs7138803 | 12 | 50247468 | A | G | 0.030 | 0.0017 | 3.1E-71 | 0.011 | 0.008 | 0.078 | 0.012 | 0.0141 | 0.408 | 0.023 | 0.0205 | 0.260 |
| rs7134628 | 12 | 53785861 | A | G | 0.017 | 0.0028 | 2.59E-09 | 0.023 | 0.013 | 0.036 | 0.017 | 0.0227 | 0.455 | 0.010 | 0.0332 | 0.767 |
| rs10783779 | 12 | 56491880 | T | G | 0.014 | 0.0018 | 5.25E-15 | -0.007 | 0.008 | 0.198 | - | - | - | - | - | - |
| rs113397893 | 12 | 57611285 | G | A | 0.022 | 0.0036 | 8.7E-10 | 0.001 | 0.016 | 0.464 | - | - | - | - | - | - |
| rs7975187 | 12 | 60964108 | G | A | 0.014 | 0.0021 | 3.86E-11 | 0.000 | 0.009 | 0.491 | - | - | - | - | - | - |
| rs650198 | 12 | 69674595 | C | T | 0.014 | 0.0019 | 6.43E-13 | -0.003 | 0.009 | 0.384 | - | - | - | - | - | - |
| rs61754230 | 12 | 72179446 | T | C | 0.045 | 0.0070 | 1.2E-10 | 0.046 | 0.041 | 0.128 | - | - | - | - | - | - |
| rs11115176 | 12 | 82465797 | T | C | 0.013 | 0.0019 | 6.79E-12 | -0.016 | 0.009 | 0.040 | - | - | - | - | - | - |
| rs704061 | 12 | 89771903 | C | T | 0.014 | 0.0017 | 8.34E-17 | 0.001 | 0.008 | 0.476 | 0.023 | 0.0153 | 0.130 | 0.041 | 0.0224 | 0.064 |
| rs11105839 | 12 | 91237920 | T | A | 0.011 | 0.0017 | 1.25E-11 | -0.005 | 0.008 | 0.263 | - | - | - | - | - | - |
| rs55966114 | 12 | 97584357 | T | C | 0.015 | 0.0024 | 2.47E-09 | 0.024 | 0.010 | 0.006 | - | - | - | - | - | - |
| rs651548 | 12 | 99560183 | A | G | 0.015 | 0.0018 | 2.38E-16 | -0.005 | 0.008 | 0.251 | - | - | - | - | - | - |
| rs6539064 | 12 | 103706754 | C | G | 0.019 | 0.0019 | 1.14E-23 | -0.007 | 0.009 | 0.219 | - | - | - | - | - | - |
| rs12316047 | 12 | 108393845 | G | A | 0.017 | 0.0020 | 1.34E-17 | -0.001 | 0.010 | 0.447 | - | - | - | - | - | - |
| rs6606686 | 12 | 110903380 | G | C | 0.016 | 0.0017 | 3.57E-19 | -0.001 | 0.008 | 0.471 | - | - | - | - | - | - |
| rs7968390 | 12 | 118414697 | A | G | 0.012 | 0.0018 | 2.43E-10 | -0.021 | 0.009 | 0.008 | 0.002 | 0.0157 | 0.900 | -0.032 | 0.0228 | 0.161 |
| rs7961979 | 12 | 121671261 | A | C | 0.018 | 0.0026 | 7.26E-12 | -0.014 | 0.012 | 0.134 | - | - | - | - | - | - |
| rs12369179 | 12 | 122963550 | C | T | 0.034 | 0.0031 | 2.32E-28 | -0.014 | 0.014 | 0.153 | - | - | - | - | - | - |
| rs7133378 | 12 | 124409502 | A | G | 0.013 | 0.0017 | 2.93E-13 | -0.017 | 0.008 | 0.020 | -0.024 | 0.0154 | 0.114 | -0.047 | 0.0224 | 0.034 |
| rs11836108 | 12 | 133391022 | A | G | 0.011 | 0.0018 | 8.37E-10 | 0.004 | 0.008 | 0.321 | - | - | - | - | - | - |
| rs1967772 | 13 | 28036062 | G | A | 0.017 | 0.0019 | 9.4E-20 | -0.030 | 0.009 | <0.001 | 0.006 | 0.0159 | 0.711 | -0.019 | 0.0229 | 0.418 |
| rs1045411 | 13 | 31033232 | C | T | 0.014 | 0.0019 | 6.66E-14 | 0.008 | 0.010 | 0.203 | -0.010 | 0.0172 | 0.553 | 0.013 | 0.0256 | 0.604 |
| rs9595908 | 13 | 33184288 | T | C | 0.015 | 0.0017 | 3.73E-20 | 0.009 | 0.008 | 0.127 | 0.003 | 0.0144 | 0.840 | 0.042 | 0.0208 | 0.042 |
| rs9603697 | 13 | 40783323 | T | C | 0.013 | 0.0018 | 1.69E-13 | 0.007 | 0.008 | 0.187 | 0.000 | 0.0149 | 0.984 | -0.020 | 0.0217 | 0.367 |
| rs41284828 | 13 | 50963685 | G | A | 0.034 | 0.0057 | 3E-09 | 0.023 | 0.021 | 0.136 | 0.039 | 0.0378 | 0.304 | 0.013 | 0.0560 | 0.812 |
| rs12429545 | 13 | 54102206 | A | G | 0.031 | 0.0024 | 1.42E-37 | -0.016 | 0.012 | 0.087 | -0.010 | 0.0212 | 0.628 | -0.019 | 0.0312 | 0.550 |
| rs12431244 | 13 | 58630651 | G | T | 0.020 | 0.0020 | 2.43E-22 | 0.010 | 0.009 | 0.126 | 0.018 | 0.0164 | 0.273 | -0.039 | 0.0237 | 0.097 |
| rs2322622 | 13 | 60497331 | C | T | 0.010 | 0.0017 | 4.64E-09 | -0.019 | 0.008 | 0.008 | -0.001 | 0.0143 | 0.944 | -0.019 | 0.0207 | 0.363 |
| rs1394879 | 13 | 62683659 | C | G | 0.011 | 0.0017 | 1.67E-10 | 0.009 | 0.008 | 0.138 | 0.025 | 0.0144 | 0.088 | -0.007 | 0.0210 | 0.748 |
| rs9540493 | 13 | 66205704 | A | G | 0.013 | 0.0017 | 7.85E-15 | 0.006 | 0.008 | 0.210 | - | - | - | - | - | - |
| rs9571687 | 13 | 67472713 | C | A | 0.012 | 0.0018 | 1.53E-11 | -0.001 | 0.008 | 0.432 | -0.031 | 0.0150 | 0.043 | -0.014 | 0.0216 | 0.523 |
| rs4421883 | 13 | 79561962 | C | T | 0.010 | 0.0017 | 1.79E-09 | 0.000 | 0.008 | 0.490 | 0.034 | 0.0144 | 0.018 | 0.013 | 0.0211 | 0.530 |
| rs77432547 | 13 | 86494817 | G | A | 0.017 | 0.0021 | 1.44E-15 | 0.000 | 0.008 | 0.486 | -0.020 | 0.0155 | 0.196 | -0.028 | 0.0223 | 0.214 |
| rs1927790 | 13 | 96922191 | C | T | 0.014 | 0.0016 | 1.57E-17 | 0.015 | 0.008 | 0.029 | 0.015 | 0.0144 | 0.288 | 0.045 | 0.0208 | 0.032 |
| rs17574378 | 13 | 99116384 | T | C | 0.014 | 0.0019 | 7.25E-14 | -0.003 | 0.008 | 0.347 | 0.017 | 0.0154 | 0.282 | -0.036 | 0.0222 | 0.108 |
| rs9514131 | 13 | 104090848 | G | T | 0.016 | 0.0026 | 2.92E-09 | 0.012 | 0.012 | 0.154 | - | - | - | - | - | - |
| rs12868881 | 13 | 112218924 | A | T | 0.014 | 0.0017 | 1.4E-15 | 0.011 | 0.008 | 0.081 | - | - | - | - | - | - |
| rs10132280 | 14 | 25928179 | C | A | 0.021 | 0.0018 | 2.28E-33 | -0.013 | 0.008 | 0.060 | -0.008 | 0.0154 | 0.599 | 0.026 | 0.0224 | 0.246 |
| rs12885454 | 14 | 29736838 | C | A | 0.018 | 0.0017 | 5.44E-26 | -0.006 | 0.008 | 0.213 | -0.004 | 0.0145 | 0.794 | -0.032 | 0.0209 | 0.128 |
| rs76420714 | 14 | 35628729 | T | C | 0.019 | 0.0032 | 3.6E-09 | 0.008 | 0.013 | 0.257 | 0.030 | 0.0238 | 0.206 | 0.026 | 0.0336 | 0.445 |
| rs872281 | 14 | 40834177 | C | T | 0.015 | 0.0023 | 1.56E-11 | -0.010 | 0.010 | 0.151 | -0.036 | 0.0177 | 0.043 | -0.013 | 0.0259 | 0.625 |
| rs12889085 | 14 | 42885336 | G | A | 0.013 | 0.0019 | 8.28E-12 | -0.015 | 0.008 | 0.030 | -0.018 | 0.0148 | 0.215 | -0.005 | 0.0212 | 0.801 |
| rs1491905 | 14 | 47296286 | T | C | 0.015 | 0.0017 | 3.92E-18 | 0.001 | 0.008 | 0.426 | 0.004 | 0.0138 | 0.797 | -0.001 | 0.0200 | 0.960 |
| rs217669 | 14 | 62360075 | C | T | 0.017 | 0.0021 | 6.27E-16 | 0.009 | 0.009 | 0.175 | 0.014 | 0.0167 | 0.396 | 0.009 | 0.0243 | 0.705 |
| rs12890931 | 14 | 69753369 | G | T | 0.010 | 0.0018 | 4.23E-09 | -0.005 | 0.008 | 0.248 | -0.026 | 0.0143 | 0.066 | -0.008 | 0.0208 | 0.705 |
| rs17105272 | 14 | 77529783 | T | C | 0.012 | 0.0018 | 1.73E-10 | 0.016 | 0.008 | 0.026 | 0.018 | 0.0152 | 0.240 | 0.011 | 0.0220 | 0.622 |
| rs7144011 | 14 | 79940383 | T | G | 0.026 | 0.0020 | 2.37E-40 | 0.003 | 0.009 | 0.389 | 0.013 | 0.0167 | 0.432 | -0.002 | 0.0243 | 0.941 |
| rs7141307 | 14 | 88375040 | C | T | 0.014 | 0.0020 | 5.95E-13 | 0.003 | 0.009 | 0.385 | 0.005 | 0.0163 | 0.784 | -0.002 | 0.0237 | 0.950 |
| rs1951455 | 14 | 91512339 | C | T | 0.015 | 0.0019 | 6.05E-15 | -0.012 | 0.009 | 0.081 | -0.016 | 0.0157 | 0.309 | -0.013 | 0.0227 | 0.555 |
| rs942066 | 14 | 94031914 | G | A | 0.020 | 0.0020 | 2.36E-24 | 0.012 | 0.008 | 0.066 | -0.003 | 0.0145 | 0.825 | -0.002 | 0.0209 | 0.917 |
| rs12147845 | 14 | 101144596 | T | C | 0.019 | 0.0027 | 4.97E-12 | 0.010 | 0.012 | 0.206 | 0.028 | 0.0218 | 0.206 | 0.017 | 0.0320 | 0.594 |
| rs4906263 | 14 | 103249127 | G | C | 0.018 | 0.0018 | 8.11E-23 | -0.005 | 0.008 | 0.284 | -0.001 | 0.0145 | 0.934 | -0.035 | 0.0210 | 0.095 |
| rs11636611 | 15 | 36391965 | T | C | 0.010 | 0.0017 | 8.85E-10 | -0.010 | 0.008 | 0.108 | -0.010 | 0.0147 | 0.477 | 0.016 | 0.0215 | 0.448 |
| rs2577947 | 15 | 42037570 | C | T | 0.014 | 0.0022 | 7.35E-10 | -0.016 | 0.010 | 0.057 | -0.015 | 0.0187 | 0.435 | -0.047 | 0.0270 | 0.080 |
| rs12439798 | 15 | 46584787 | T | G | 0.013 | 0.0017 | 1.21E-13 | 0.011 | 0.008 | 0.078 | 0.020 | 0.0139 | 0.148 | 0.001 | 0.0200 | 0.976 |
| rs12912380 | 15 | 47947291 | C | T | 0.017 | 0.0027 | 1.52E-10 | 0.003 | 0.012 | 0.396 | -0.022 | 0.0224 | 0.337 | -0.011 | 0.0320 | 0.727 |
| rs6493498 | 15 | 51754451 | T | C | 0.014 | 0.0016 | 4.84E-17 | 0.001 | 0.008 | 0.474 | -0.016 | 0.0138 | 0.252 | -0.012 | 0.0199 | 0.550 |
| rs8024806 | 15 | 53473990 | T | C | 0.025 | 0.0035 | 6.73E-13 | -0.031 | 0.016 | 0.027 | -0.016 | 0.0292 | 0.588 | -0.039 | 0.0425 | 0.358 |
| rs1426652 | 15 | 59056712 | A | G | 0.015 | 0.0023 | 1.62E-10 | -0.005 | 0.011 | 0.322 | -0.023 | 0.0195 | 0.234 | -0.001 | 0.0284 | 0.960 |
| rs340025 | 15 | 60908307 | C | T | 0.013 | 0.0016 | 1.4E-14 | 0.009 | 0.008 | 0.119 | 0.011 | 0.0144 | 0.427 | -0.029 | 0.0208 | 0.170 |
| rs12438629 | 15 | 62122539 | C | G | 0.035 | 0.0052 | 2.09E-11 | 0.013 | 0.023 | 0.294 | -0.043 | 0.0413 | 0.297 | -0.005 | 0.0644 | 0.944 |
| rs11629783 | 15 | 66741387 | C | G | 0.014 | 0.0020 | 1.43E-12 | -0.051 | 0.009 | <0.001 | -0.051 | 0.0163 | 0.002 | -0.095 | 0.0235 | 0.000 |
| rs3865018 | 15 | 67892766 | C | T | 0.021 | 0.0018 | 1.86E-29 | -0.016 | 0.009 | 0.033 | -0.018 | 0.0161 | 0.269 | -0.013 | 0.0229 | 0.561 |
| rs730180 | 15 | 73007893 | A | G | 0.017 | 0.0020 | 1.8E-18 | 0.004 | 0.009 | 0.330 | 0.009 | 0.0171 | 0.593 | 0.040 | 0.0249 | 0.110 |
| rs12914489 | 15 | 74187937 | A | G | 0.017 | 0.0027 | 8.78E-11 | 0.030 | 0.012 | 0.007 | 0.021 | 0.0221 | 0.345 | 0.096 | 0.0323 | 0.003 |
| rs11856579 | 15 | 78012688 | G | A | 0.016 | 0.0019 | 1.38E-16 | 0.008 | 0.009 | 0.179 | 0.012 | 0.0160 | 0.457 | 0.017 | 0.0231 | 0.466 |
| rs8038464 | 15 | 79463036 | T | A | 0.013 | 0.0017 | 1.56E-13 | -0.013 | 0.008 | 0.052 | -0.015 | 0.0144 | 0.300 | -0.035 | 0.0208 | 0.097 |
| rs12914623 | 15 | 80993570 | G | C | 0.016 | 0.0019 | 2E-16 | 0.024 | 0.009 | 0.003 | 0.021 | 0.0160 | 0.186 | 0.067 | 0.0231 | 0.004 |
| rs7498044 | 15 | 92573639 | G | A | 0.016 | 0.0024 | 4.89E-12 | 0.006 | 0.010 | 0.263 | 0.011 | 0.0174 | 0.542 | 0.001 | 0.0256 | 0.959 |
| rs11633626 | 15 | 95271378 | C | A | 0.016 | 0.0018 | 7.27E-19 | -0.007 | 0.008 | 0.195 | -0.021 | 0.0143 | 0.151 | -0.025 | 0.0208 | 0.233 |
| rs58139454 | 15 | 99230162 | G | C | 0.015 | 0.0023 | 2.57E-10 | 0.016 | 0.009 | 0.046 | 0.008 | 0.0168 | 0.644 | 0.027 | 0.0245 | 0.266 |
| rs11866815 | 16 | 387867 | C | T | 0.015 | 0.0019 | 2.21E-16 | -0.019 | 0.009 | 0.016 | -0.003 | 0.0167 | 0.874 | -0.015 | 0.0237 | 0.519 |
| rs2601777 | 16 | 4035068 | G | A | 0.014 | 0.0017 | 2.84E-17 | 0.010 | 0.008 | 0.108 | 0.015 | 0.0145 | 0.302 | 0.017 | 0.0210 | 0.422 |
| rs2058527 | 16 | 6704749 | G | T | 0.012 | 0.0019 | 1.8E-09 | 0.010 | 0.009 | 0.132 | 0.008 | 0.0158 | 0.616 | -0.002 | 0.0229 | 0.949 |
| rs1990573 | 16 | 9713688 | G | A | 0.013 | 0.0018 | 2.11E-13 | -0.001 | 0.008 | 0.438 | -0.008 | 0.0154 | 0.598 | 0.019 | 0.0225 | 0.390 |
| rs4985155 | 16 | 15129459 | A | G | 0.010 | 0.0017 | 3.39E-09 | 0.017 | 0.008 | 0.015 | 0.030 | 0.0147 | 0.042 | 0.051 | 0.0213 | 0.018 |
| rs12446632 | 16 | 19935389 | G | A | 0.035 | 0.0024 | 3.12E-50 | -0.014 | 0.011 | 0.100 | - | - | - | - | - | - |
| rs2342892 | 16 | 24540806 | T | G | 0.013 | 0.0017 | 1.33E-13 | 0.003 | 0.008 | 0.346 | 0.003 | 0.0149 | 0.833 | 0.026 | 0.0218 | 0.231 |
| rs7498665 | 16 | 28883241 | G | A | 0.029 | 0.0017 | 1.14E-66 | 0.003 | 0.008 | 0.369 | -0.007 | 0.0143 | 0.646 | -0.015 | 0.0206 | 0.482 |
| rs4609871 | 16 | 29932064 | T | C | 0.022 | 0.0017 | 6.62E-38 | -0.025 | 0.008 | <0.001 | -0.039 | 0.0142 | 0.006 | -0.029 | 0.0206 | 0.165 |
| rs7190997 | 16 | 31368178 | T | C | 0.014 | 0.0016 | 2.76E-18 | -0.006 | 0.008 | 0.215 | -0.010 | 0.0138 | 0.471 | -0.019 | 0.0201 | 0.341 |
| rs6500208 | 16 | 49011249 | A | G | 0.015 | 0.0020 | 3.21E-13 | -0.011 | 0.010 | 0.135 | -0.001 | 0.0175 | 0.957 | -0.044 | 0.0259 | 0.086 |
| rs7193144 | 16 | 53810686 | C | T | 0.073 | 0.0017 | 1E-200 | -0.013 | 0.008 | 0.051 | -0.005 | 0.0142 | 0.742 | 0.003 | 0.0204 | 0.884 |
| rs7189122 | 16 | 56471410 | C | T | 0.015 | 0.0022 | 4.31E-11 | -0.008 | 0.010 | 0.224 | -0.009 | 0.0180 | 0.621 | -0.053 | 0.0263 | 0.046 |
| rs11075489 | 16 | 62803841 | C | T | 0.012 | 0.0017 | 1.23E-11 | 0.008 | 0.008 | 0.135 | -0.003 | 0.0139 | 0.849 | 0.013 | 0.0201 | 0.517 |
| rs2307022 | 16 | 68381978 | A | G | 0.013 | 0.0017 | 7.57E-15 | -0.015 | 0.008 | 0.035 | - | - | - | - | - | - |
| rs889398 | 16 | 69556715 | C | T | 0.020 | 0.0016 | 3.23E-32 | 0.002 | 0.008 | 0.418 | - | - | - | - | - | - |
| rs811054 | 16 | 72251132 | T | C | 0.014 | 0.0016 | 2.36E-16 | -0.005 | 0.008 | 0.258 | -0.016 | 0.0141 | 0.245 | -0.017 | 0.0203 | 0.404 |
| rs12449219 | 16 | 77261943 | G | C | 0.016 | 0.0026 | 1.44E-10 | 0.009 | 0.012 | 0.214 | -0.004 | 0.0212 | 0.871 | -0.012 | 0.0300 | 0.691 |
| rs2012502 | 16 | 81728081 | A | C | 0.012 | 0.0017 | 1.9E-12 | 0.000 | 0.008 | 0.485 | 0.016 | 0.0145 | 0.281 | -0.009 | 0.0212 | 0.676 |
| rs7206608 | 16 | 82872628 | G | C | 0.013 | 0.0018 | 1.2E-12 | -0.002 | 0.008 | 0.402 | 0.001 | 0.0147 | 0.956 | -0.016 | 0.0213 | 0.467 |
| rs3923783 | 17 | 1843189 | C | A | 0.022 | 0.0022 | 4.12E-23 | 0.008 | 0.010 | 0.198 | 0.019 | 0.0182 | 0.303 | 0.003 | 0.0264 | 0.900 |
| rs1000940 | 17 | 5283252 | G | A | 0.015 | 0.0018 | 7.8E-18 | -0.010 | 0.008 | 0.118 | -0.022 | 0.0149 | 0.149 | -0.001 | 0.0216 | 0.978 |
| rs1075901 | 17 | 15943910 | C | T | 0.012 | 0.0016 | 4.43E-13 | 0.010 | 0.008 | 0.086 | -0.020 | 0.0140 | 0.146 | 0.022 | 0.0202 | 0.269 |
| rs1320251 | 17 | 21264396 | C | T | 0.018 | 0.0017 | 5.51E-25 | 0.012 | 0.008 | 0.068 | 0.020 | 0.0144 | 0.168 | 0.011 | 0.0208 | 0.602 |
| rs4795195 | 17 | 26318587 | A | G | 0.011 | 0.0019 | 3.33E-09 | -0.008 | 0.009 | 0.192 | -0.006 | 0.0158 | 0.707 | -0.009 | 0.0230 | 0.702 |
| rs1038088 | 17 | 28074563 | G | T | 0.012 | 0.0016 | 1.37E-12 | 0.014 | 0.008 | 0.038 | 0.014 | 0.0140 | 0.330 | 0.001 | 0.0203 | 0.955 |
| rs3930349 | 17 | 31475545 | C | A | 0.014 | 0.0020 | 7.68E-12 | 0.006 | 0.009 | 0.274 | 0.033 | 0.0171 | 0.054 | -0.004 | 0.0248 | 0.876 |
| rs1106908 | 17 | 34942595 | G | A | 0.016 | 0.0016 | 7.6E-23 | -0.002 | 0.008 | 0.414 | 0.009 | 0.0152 | 0.562 | 0.004 | 0.0227 | 0.877 |
| rs7219230 | 17 | 39286180 | T | C | 0.011 | 0.0018 | 4.87E-10 | 0.004 | 0.008 | 0.317 | 0.014 | 0.0152 | 0.373 | 0.009 | 0.0219 | 0.686 |
| rs2670854 | 17 | 41085683 | A | G | 0.011 | 0.0019 | 1.97E-09 | 0.000 | 0.009 | 0.495 | 0.010 | 0.0159 | 0.541 | 0.000 | 0.0231 | 0.993 |
| rs11655587 | 17 | 47140794 | C | T | 0.021 | 0.0020 | 6.87E-26 | -0.009 | 0.008 | 0.133 | 0.002 | 0.0149 | 0.910 | -0.013 | 0.0216 | 0.543 |
| rs11649864 | 17 | 56093061 | A | G | 0.019 | 0.0030 | 2.52E-10 | 0.005 | 0.014 | 0.357 | 0.004 | 0.0264 | 0.888 | 0.045 | 0.0372 | 0.225 |
| rs8075273 | 17 | 61728881 | C | A | 0.014 | 0.0018 | 4.67E-14 | 0.016 | 0.008 | 0.033 | -0.019 | 0.0152 | 0.215 | 0.018 | 0.0220 | 0.422 |
| rs2619976 | 17 | 71754545 | T | C | 0.011 | 0.0018 | 1.99E-09 | 0.010 | 0.008 | 0.107 | -0.005 | 0.0151 | 0.733 | 0.038 | 0.0220 | 0.084 |
| rs3744017 | 17 | 73871467 | A | G | 0.014 | 0.0022 | 2.43E-10 | 0.007 | 0.010 | 0.254 | 0.007 | 0.0191 | 0.717 | 0.020 | 0.0275 | 0.466 |
| rs8081039 | 17 | 75995829 | T | C | 0.023 | 0.0037 | 6.02E-10 | -0.007 | 0.016 | 0.319 | 0.032 | 0.0288 | 0.269 | 0.057 | 0.0424 | 0.180 |
| rs12939549 | 17 | 78611724 | A | G | 0.018 | 0.0016 | 3.68E-28 | -0.014 | 0.008 | 0.042 | 0.003 | 0.0143 | 0.823 | -0.005 | 0.0207 | 0.828 |
| rs7238896 | 18 | 1840658 | G | A | 0.022 | 0.0028 | 2.49E-15 | -0.019 | 0.011 | 0.036 | 0.010 | 0.0197 | 0.613 | -0.005 | 0.0289 | 0.872 |
| rs891387 | 18 | 21103909 | T | C | 0.021 | 0.0017 | 9.26E-35 | 0.017 | 0.008 | 0.013 | 0.022 | 0.0140 | 0.118 | 0.037 | 0.0203 | 0.065 |
| rs1941697 | 18 | 31251276 | A | G | 0.012 | 0.0017 | 8.36E-13 | -0.002 | 0.008 | 0.410 | 0.002 | 0.0136 | 0.893 | 0.006 | 0.0198 | 0.745 |
| rs1356506 | 18 | 40708038 | T | C | 0.014 | 0.0018 | 8.39E-15 | 0.015 | 0.008 | 0.032 | 0.014 | 0.0144 | 0.347 | 0.021 | 0.0209 | 0.325 |
| rs11874040 | 18 | 42595996 | A | G | 0.013 | 0.0019 | 1.54E-11 | 0.006 | 0.008 | 0.242 | 0.000 | 0.0151 | 0.992 | 0.011 | 0.0220 | 0.624 |
| rs9965170 | 18 | 44788274 | G | A | 0.011 | 0.0017 | 1.02E-09 | 0.006 | 0.008 | 0.204 | -0.006 | 0.0136 | 0.659 | 0.010 | 0.0198 | 0.611 |
| rs7239114 | 18 | 45921214 | A | G | 0.012 | 0.0017 | 6.24E-13 | 0.009 | 0.008 | 0.138 | - | - | - | - | - | - |
| rs6567160 | 18 | 57829135 | C | T | 0.055 | 0.0019 | 7.82E-184 | -0.016 | 0.009 | 0.034 | -0.014 | 0.0166 | 0.393 | -0.036 | 0.0240 | 0.131 |
| rs12454712 | 18 | 60845884 | C | T | 0.014 | 0.0018 | 1.05E-14 | 0.003 | 0.008 | 0.341 | - | - | - | - | - | - |
| rs10871589 | 18 | 63285783 | G | A | 0.016 | 0.0020 | 1.03E-15 | 0.001 | 0.008 | 0.455 | -0.002 | 0.0145 | 0.896 | 0.048 | 0.0211 | 0.024 |
| rs594821 | 18 | 76745589 | C | T | 0.019 | 0.0030 | 3.22E-10 | -0.062 | 0.014 | <0.001 | - | - | - | - | - | - |
| rs3746038 | 19 | 1852494 | C | T | 0.017 | 0.0021 | 2.8E-16 | -0.025 | 0.010 | 0.004 | 0.002 | 0.0172 | 0.893 | -0.060 | 0.0248 | 0.016 |
| rs56356382 | 19 | 4064057 | T | C | 0.022 | 0.0024 | 3.21E-19 | -0.001 | 0.010 | 0.457 | -0.041 | 0.0182 | 0.024 | 0.031 | 0.0266 | 0.241 |
| rs2072597 | 19 | 12996740 | G | A | 0.013 | 0.0021 | 1.52E-09 | -0.020 | 0.009 | 0.009 | -0.016 | 0.0154 | 0.295 | -0.008 | 0.0224 | 0.719 |
| rs12462975 | 19 | 30272202 | A | G | 0.019 | 0.0018 | 1.47E-25 | 0.011 | 0.008 | 0.083 | 0.011 | 0.0148 | 0.467 | 0.004 | 0.0213 | 0.851 |
| rs29938 | 19 | 34311481 | C | T | 0.016 | 0.0017 | 3.81E-20 | 0.004 | 0.008 | 0.328 | 0.012 | 0.0150 | 0.438 | -0.007 | 0.0216 | 0.738 |
| rs11672660 | 19 | 46180184 | C | T | 0.034 | 0.0021 | 6.83E-60 | 0.008 | 0.010 | 0.205 | 0.023 | 0.0174 | 0.195 | 0.023 | 0.0261 | 0.377 |
| rs12151152 | 19 | 47563532 | G | A | 0.021 | 0.0020 | 6.51E-27 | -0.024 | 0.008 | 0.001 | -0.042 | 0.0147 | 0.005 | -0.025 | 0.0213 | 0.245 |
| rs4802570 | 19 | 49646476 | C | G | 0.012 | 0.0020 | 3.22E-09 | -0.004 | 0.008 | 0.289 | 0.002 | 0.0146 | 0.884 | 0.044 | 0.0211 | 0.038 |
| rs148108087 | 19 | 51781455 | G | A | 0.030 | 0.0045 | 2.34E-11 | -0.013 | 0.017 | 0.217 | 0.000 | 0.0312 | 1.000 | -0.050 | 0.0453 | 0.275 |
| rs1884389 | 20 | 1410582 | C | T | 0.011 | 0.0017 | 3.72E-10 | -0.003 | 0.008 | 0.332 | -0.014 | 0.0139 | 0.317 | 0.010 | 0.0201 | 0.612 |
| rs615568 | 20 | 3008775 | G | T | 0.010 | 0.0017 | 3.85E-09 | -0.004 | 0.008 | 0.316 | 0.007 | 0.0137 | 0.619 | -0.025 | 0.0199 | 0.216 |
| rs1884897 | 20 | 6612832 | G | A | 0.018 | 0.0017 | 2.7E-28 | -0.004 | 0.008 | 0.300 | 0.019 | 0.0146 | 0.191 | -0.013 | 0.0211 | 0.550 |
| rs16996644 | 20 | 15813475 | G | C | 0.018 | 0.0026 | 1.6E-12 | 0.007 | 0.012 | 0.287 | -0.016 | 0.0218 | 0.472 | 0.014 | 0.0322 | 0.666 |
| rs947088 | 20 | 17171373 | T | G | 0.013 | 0.0019 | 3.51E-11 | 0.001 | 0.008 | 0.457 | - | - | - | - | - | - |
| rs1409818 | 20 | 21381121 | T | C | 0.020 | 0.0028 | 2.59E-12 | -0.014 | 0.013 | 0.132 | -0.038 | 0.0239 | 0.112 | -0.017 | 0.0343 | 0.630 |
| rs8122855 | 20 | 25192049 | A | G | 0.014 | 0.0018 | 4.11E-14 | -0.021 | 0.008 | 0.006 | -0.022 | 0.0148 | 0.136 | -0.027 | 0.0214 | 0.204 |
| rs1987960 | 20 | 30649834 | C | T | 0.029 | 0.0047 | 4.56E-10 | 0.012 | 0.023 | 0.307 | 0.033 | 0.0421 | 0.438 | -0.002 | 0.0594 | 0.968 |
| rs6142067 | 20 | 32556572 | T | C | 0.013 | 0.0017 | 3.55E-13 | -0.022 | 0.008 | 0.002 | - | - | - | - | - | - |
| rs16989232 | 20 | 39291784 | A | G | 0.012 | 0.0017 | 6.19E-12 | -0.004 | 0.008 | 0.315 | -0.010 | 0.0149 | 0.503 | -0.037 | 0.0216 | 0.083 |
| rs6130360 | 20 | 42010996 | A | G | 0.016 | 0.0024 | 7.49E-12 | 0.007 | 0.011 | 0.266 | 0.017 | 0.0198 | 0.395 | -0.048 | 0.0279 | 0.083 |
| rs2425857 | 20 | 44914134 | A | G | 0.012 | 0.0017 | 1.67E-11 | 0.013 | 0.008 | 0.047 | 0.012 | 0.0145 | 0.425 | 0.006 | 0.0210 | 0.779 |
| rs6019483 | 20 | 47495656 | T | A | 0.018 | 0.0023 | 3.68E-14 | -0.008 | 0.010 | 0.227 | -0.016 | 0.0190 | 0.415 | -0.029 | 0.0277 | 0.296 |
| rs17806224 | 20 | 51065854 | G | A | 0.026 | 0.0022 | 7.91E-32 | -0.014 | 0.010 | 0.087 | -0.002 | 0.0185 | 0.899 | -0.019 | 0.0268 | 0.486 |
| rs6023633 | 20 | 53453850 | G | A | 0.013 | 0.0021 | 2.32E-10 | 0.010 | 0.010 | 0.149 | 0.022 | 0.0177 | 0.209 | 0.041 | 0.0259 | 0.112 |
| rs6512302 | 20 | 62691550 | C | G | 0.013 | 0.0020 | 1.52E-11 | 0.005 | 0.009 | 0.288 | 0.015 | 0.0167 | 0.361 | -0.026 | 0.0240 | 0.280 |
| rs2832283 | 21 | 30690558 | A | G | 0.012 | 0.0020 | 4.72E-09 | -0.002 | 0.009 | 0.417 | 0.018 | 0.0166 | 0.290 | -0.033 | 0.0243 | 0.171 |
| rs17193211 | 21 | 38885506 | C | T | 0.021 | 0.0035 | 1.74E-09 | 0.012 | 0.016 | 0.228 | 0.018 | 0.0292 | 0.543 | -0.033 | 0.0425 | 0.440 |
| rs8134638 | 21 | 40644170 | C | T | 0.013 | 0.0020 | 1.57E-11 | 0.007 | 0.008 | 0.189 | 0.014 | 0.0142 | 0.330 | 0.017 | 0.0206 | 0.399 |
| rs4818226 | 21 | 42633065 | G | A | 0.012 | 0.0018 | 1.92E-11 | 0.000 | 0.008 | 0.495 | -0.014 | 0.0146 | 0.337 | 0.015 | 0.0213 | 0.477 |
| rs427943 | 21 | 46570896 | C | A | 0.018 | 0.0017 | 3.6E-25 | -0.001 | 0.008 | 0.458 | -0.011 | 0.0138 | 0.419 | -0.034 | 0.0200 | 0.087 |
| rs140733155 | 21 | 48048773 | G | A | 0.056 | 0.0094 | 2.97E-09 | 0.015 | 0.039 | 0.355 | -0.015 | 0.0718 | 0.834 | 0.068 | 0.1076 | 0.530 |
| rs12628891 | 22 | 38317137 | C | T | 0.012 | 0.0019 | 5.85E-10 | -0.011 | 0.008 | 0.099 | -0.011 | 0.0152 | 0.452 | 0.012 | 0.0219 | 0.581 |
| rs12628051 | 22 | 40654276 | T | C | 0.016 | 0.0018 | 2.9E-19 | 0.026 | 0.008 | <0.001 | 0.036 | 0.0143 | 0.011 | 0.031 | 0.0208 | 0.133 |
| rs713763 | 22 | 48871019 | A | G | 0.014 | 0.0020 | 3.52E-12 | -0.027 | 0.008 | 0.001 | -0.034 | 0.0149 | 0.022 | -0.037 | 0.0217 | 0.089 |


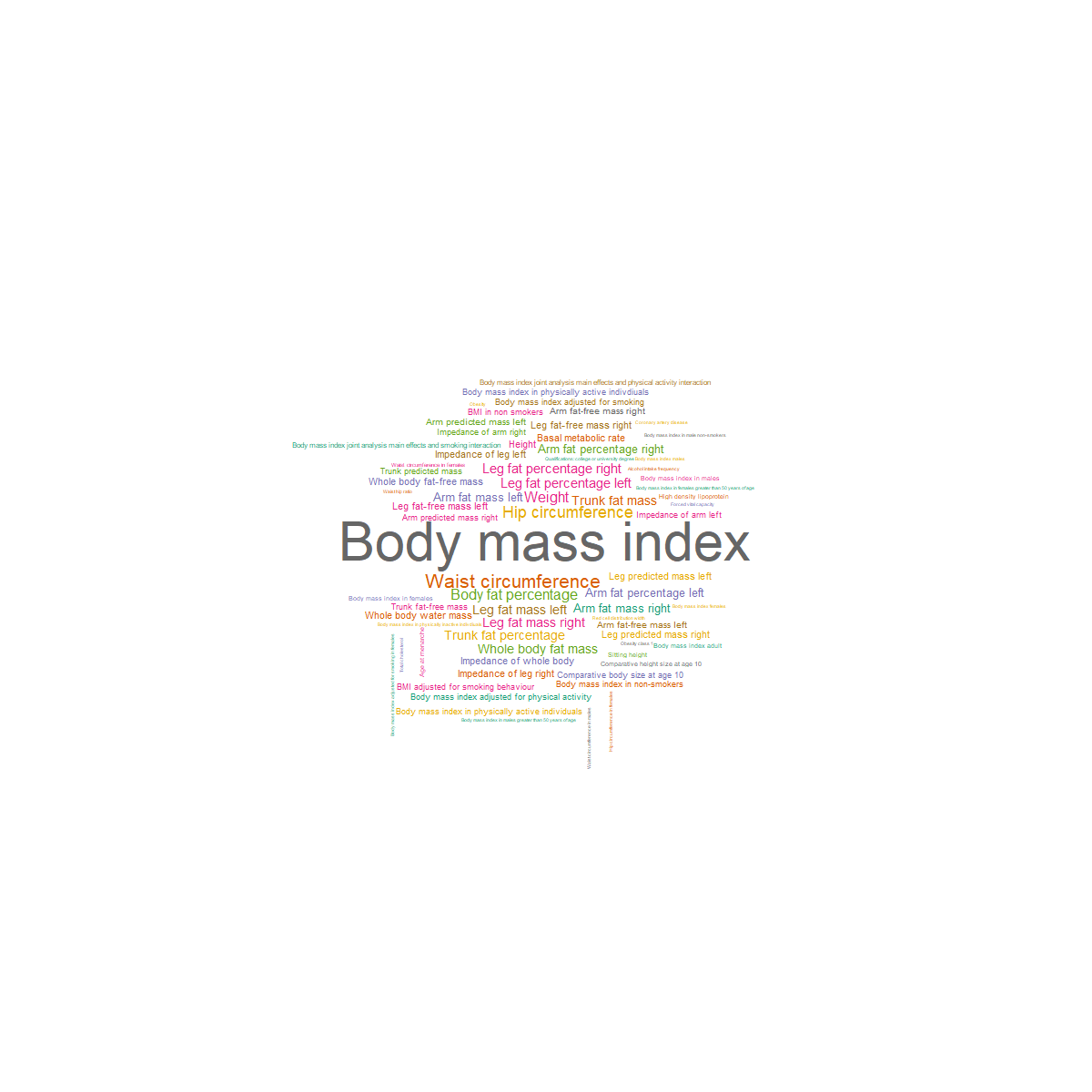


# Supplementary Figure 1: Traits associated with each metabolically unfavourable adiposity SNP used in the Mendelian randomization analyses.

Traits were identified using PhenoScanner resource [1, 2]. Larger words represent a greater frequency of the traits being associated with the SNPs (P threshold=5 x 10^-8^). This figure was created using the wordcloud package in R.

Abbreviations: SNP=single nucleotide polymorphism.


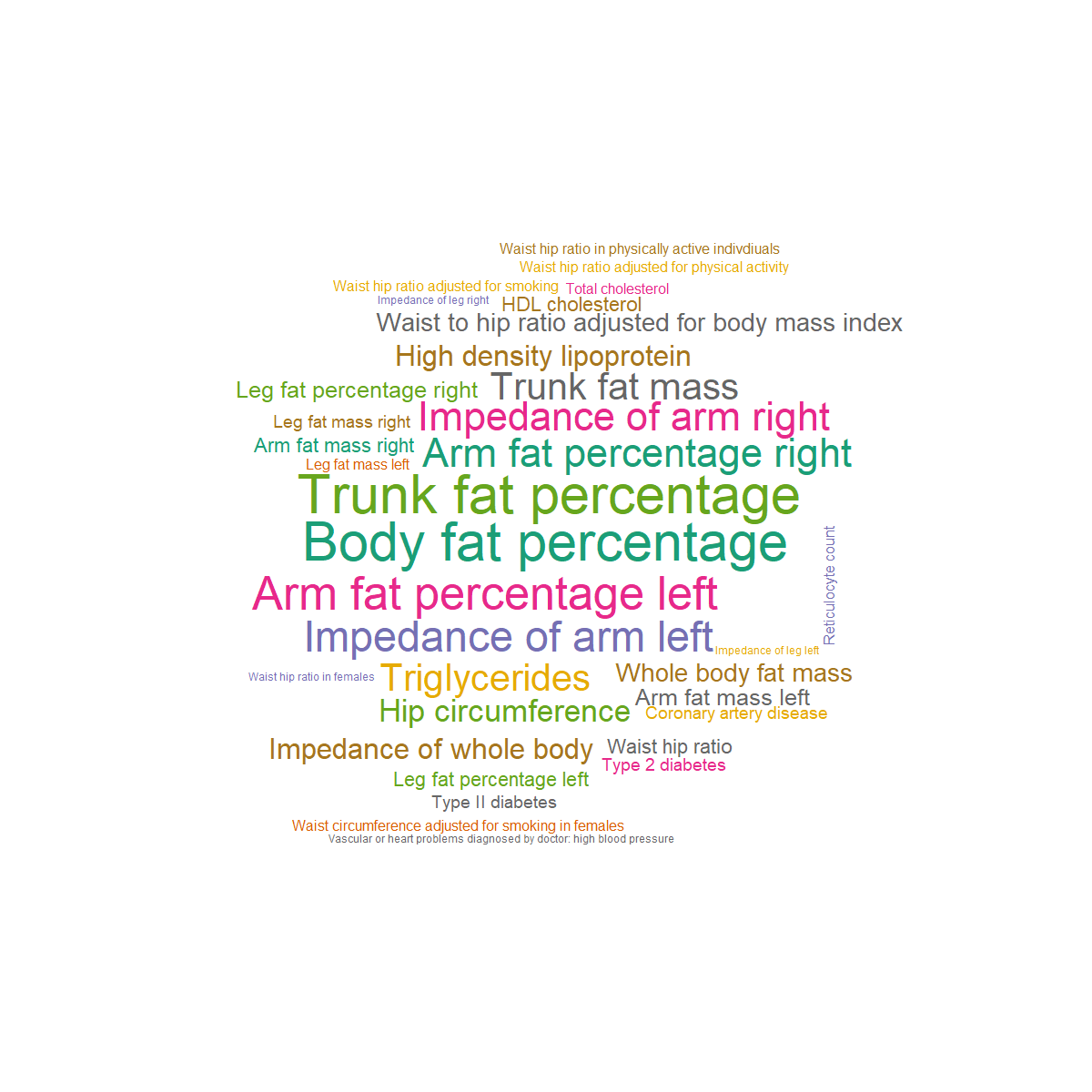


# Supplementary Figure 2: Traits associated with each metabolically favourable adiposity SNP used in the Mendelian randomization analyses.

Traits were identified using PhenoScanner resource [1, 2]. Larger words represent a greater frequency of the traits being associated with the SNPs (P threshold=5 x 10^-8^)[1, 2]. This figure was created using the wordcloud package in R.

Abbreviations: SNP=single nucleotide polymorphism.


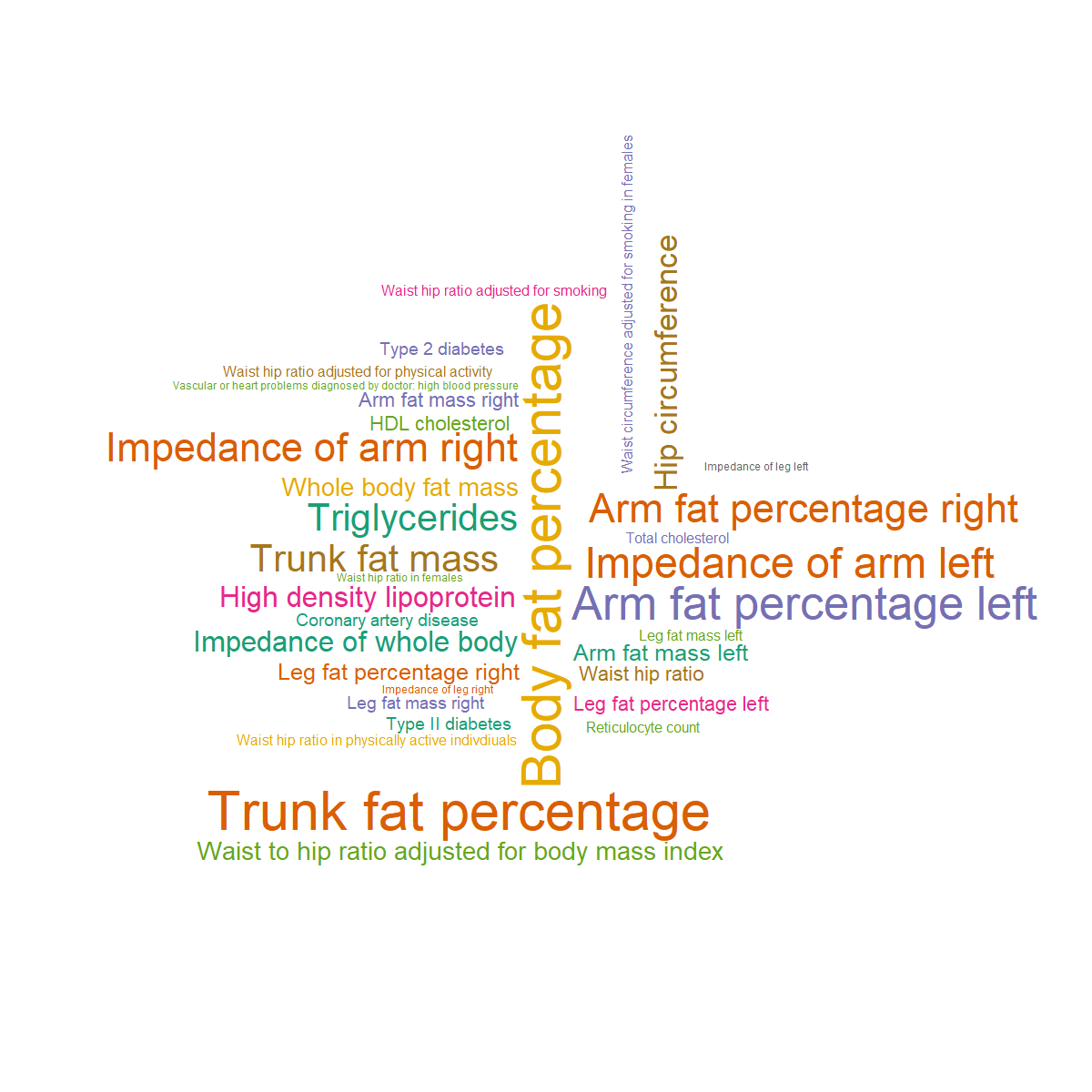


# Supplementary Figure 3: Traits associated with each BMI SNP used in the Mendelian randomization analyses.

Traits were identified using PhenoScanner resource [1, 2]. Larger words represent a greater frequency of the traits being associated with the SNPs (P threshold=5 x 10^-8^). This figure was created using the wordcloud package in R.

Abbreviations: SNP=single nucleotide polymorphism.

**A) B)**


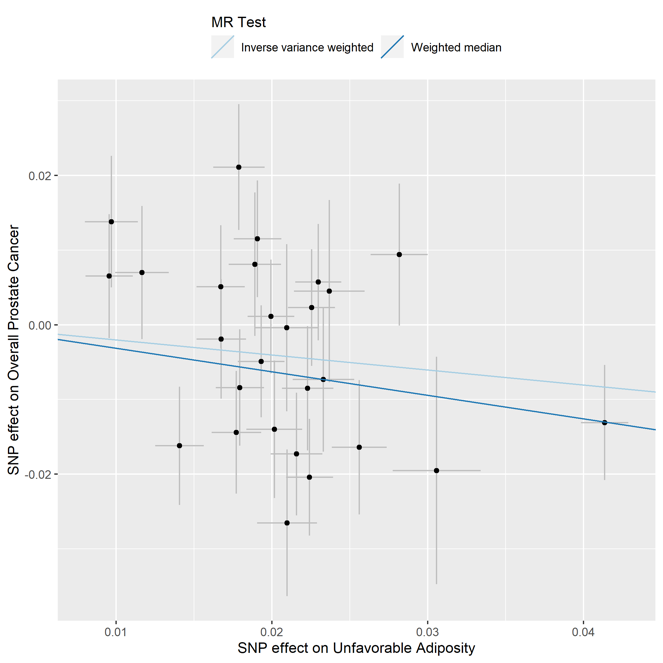

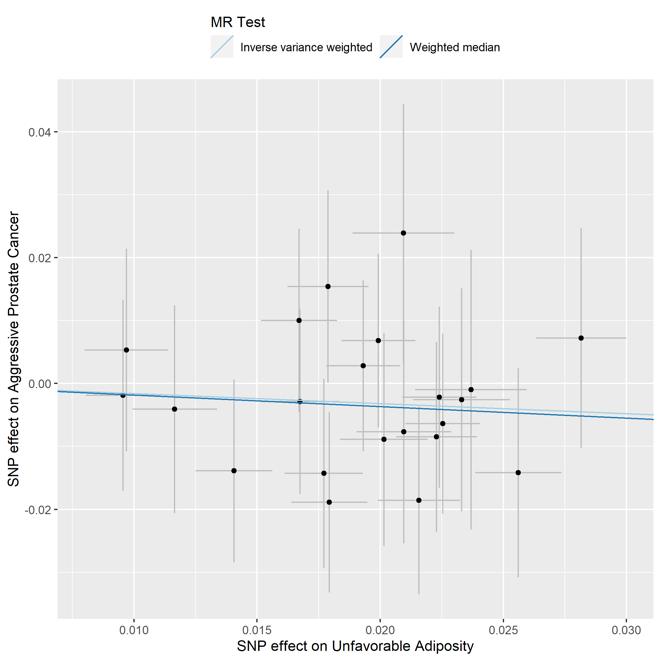


**C)**


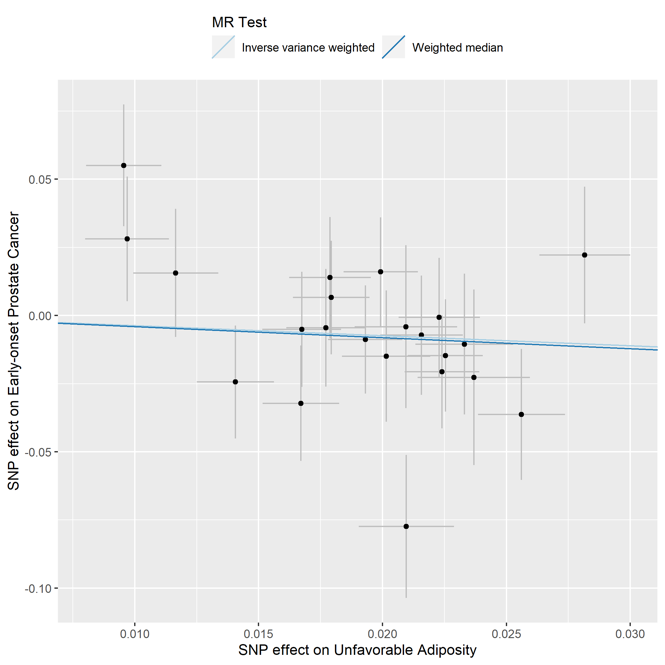


# Supplementary Figure 4: Scatterplot of genetic associations with metabolically unfavourable adiposity against genetic associations with: A) Overall prostate cancer, B) Aggressive prostate cancer, C) Early-onset prostate cancer.

Aggressive cancer defined as Gleason grade 8+, or prostate cancer death, or metastases or PSA >100 ng/mL, early-onset defined as diagnosed. Early-onset prostate cancer defined as prostate cancer cases ≤55 years. Point estimates represent log odds ratios. Error bars represent 95% confidence intervals.

Abbreviations: PSA=prostate-specific antigen; SNP=single nucleotide repeat polymorphism.

**A) B)**


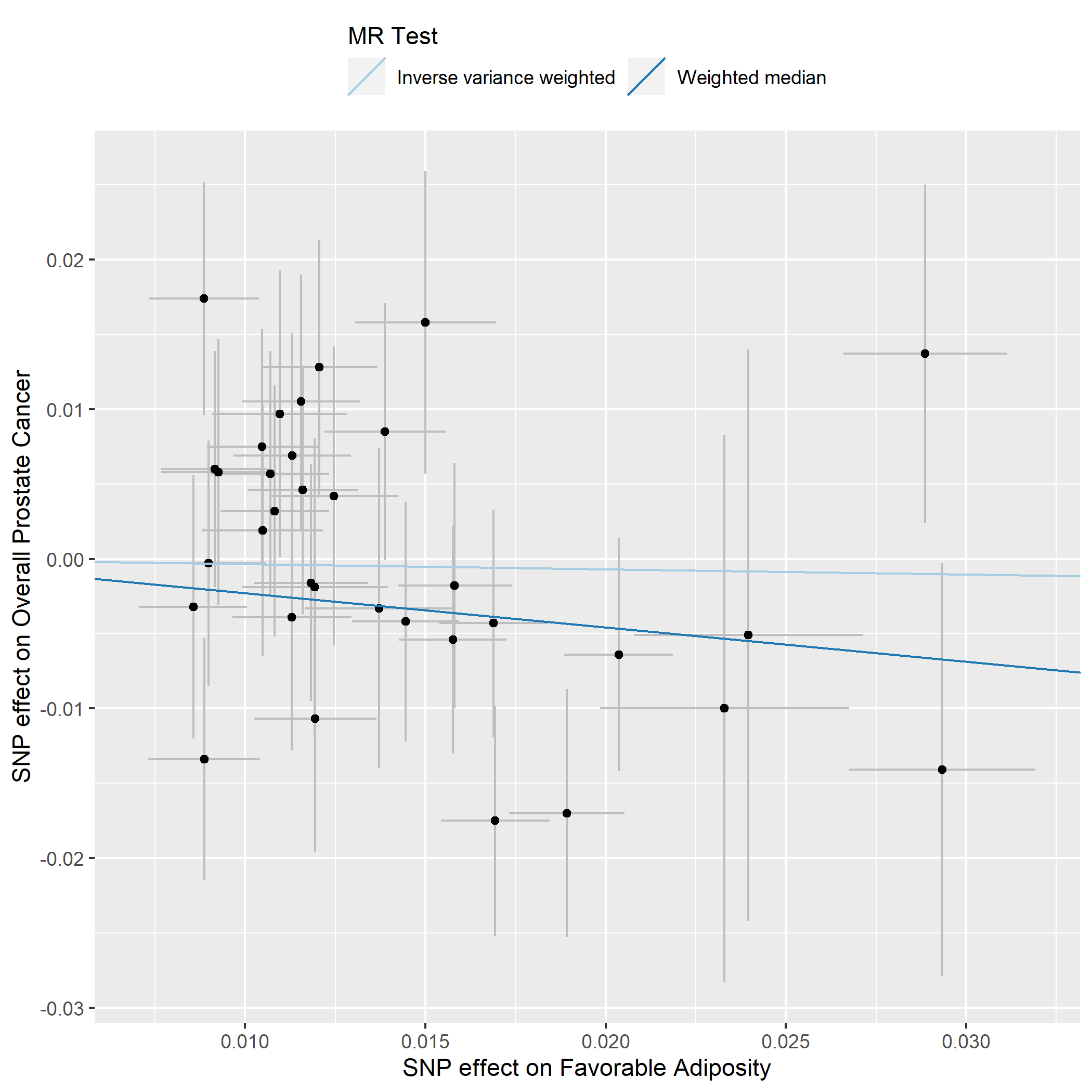

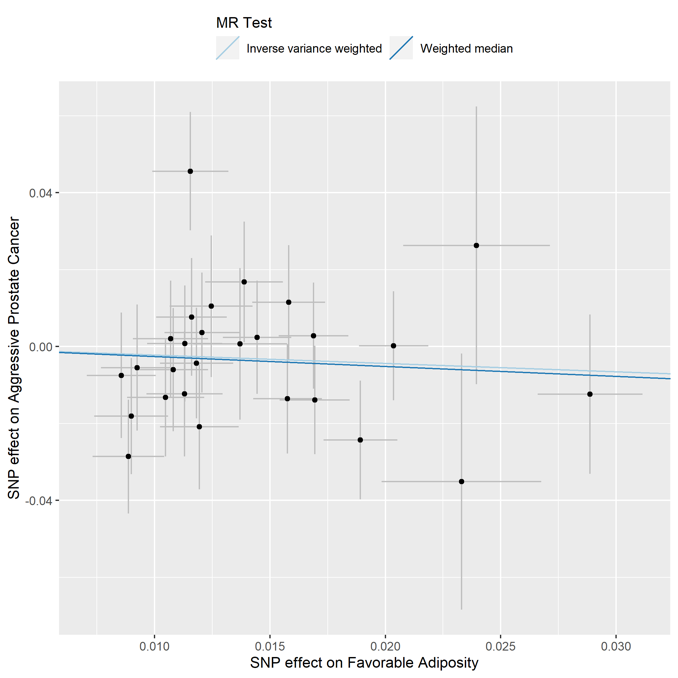


**C)**


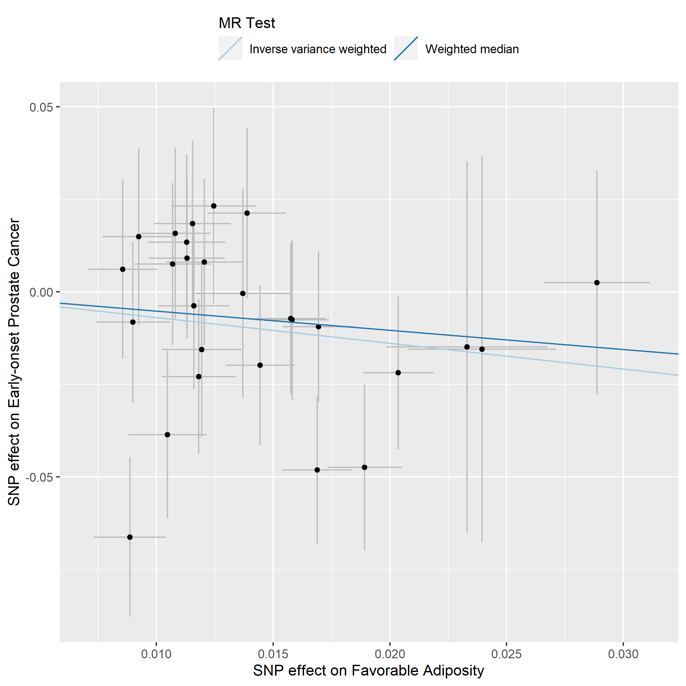


# Supplementary Figure 5: Scatterplot of genetic associations with metabolically favourable adiposity against genetic associations with: A) Overall prostate cancer, B) Aggressive prostate cancer, C) Early-onset prostate cancer.

Aggressive cancer defined as Gleason grade 8+, or prostate cancer death, or metastases or PSA >100 ng/mL, early-onset defined as diagnosed. Early-onset prostate cancer defined as prostate cancer cases ≤55 years. Point estimates represent log odds ratios. Error bars represent 95% confidence intervals.

Abbreviations: PSA=prostate-specific antigen; SNP=single nucleotide repeat polymorphism.

**A) B)**


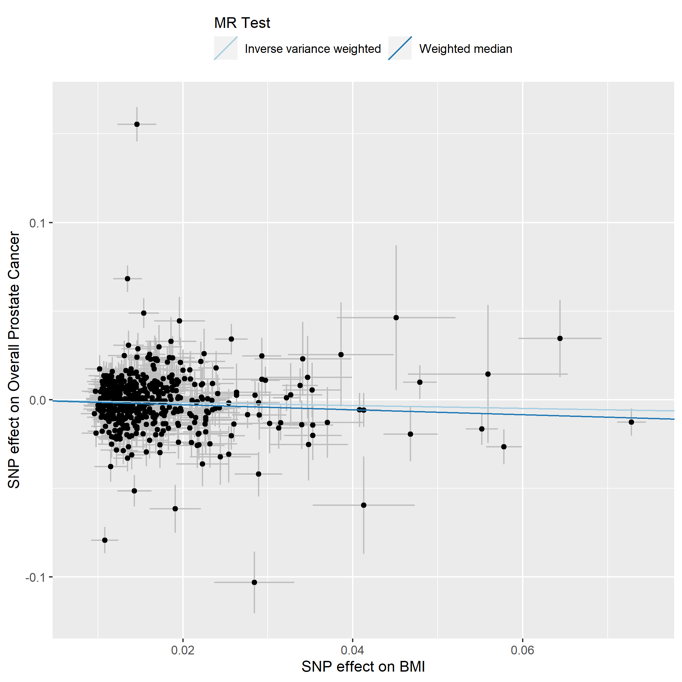

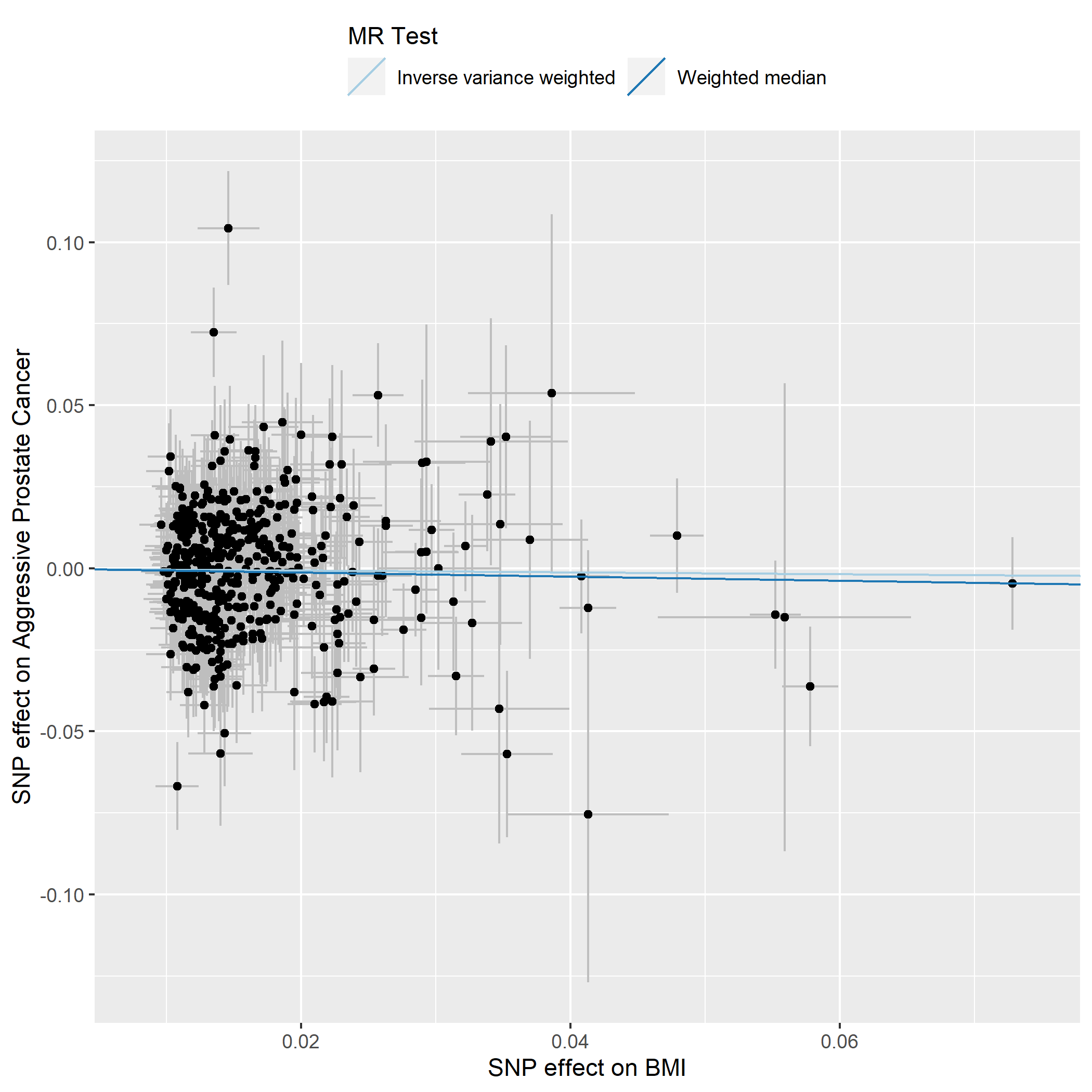


**C)**


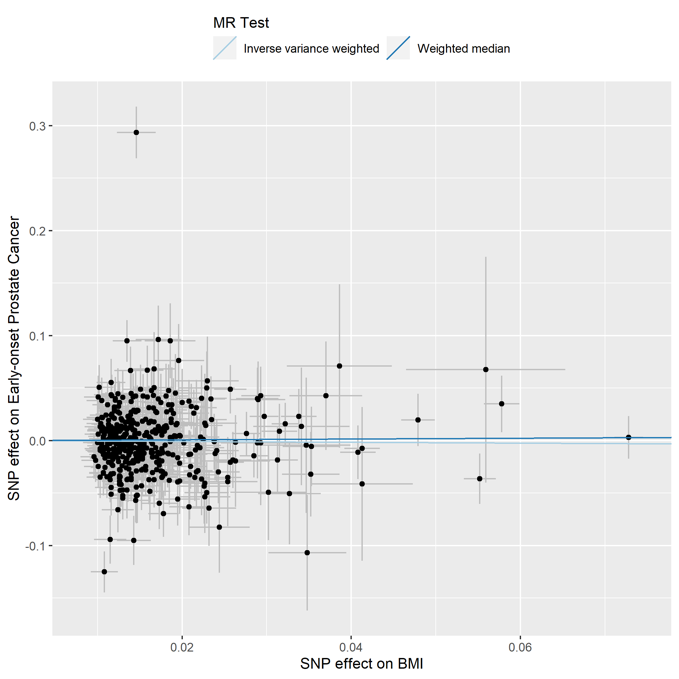


# Supplementary Figure 6: Scatterplot of genetic associations with BMI against genetic associations with: A) Overall prostate cancer, B) Aggressive prostate cancer, C) Early-onset prostate cancer.

Aggressive cancer defined as Gleason grade 8+, or prostate cancer death, or metastases or PSA >100 ng/mL, early-onset defined as diagnosed. Early-onset prostate cancer defined as prostate cancer cases ≤55 years. Point estimates represent log odds ratios. Error bars represent 95% confidence intervals.

Abbreviations: PSA=prostate-specific antigen; SNP=single nucleotide repeat polymorphism.


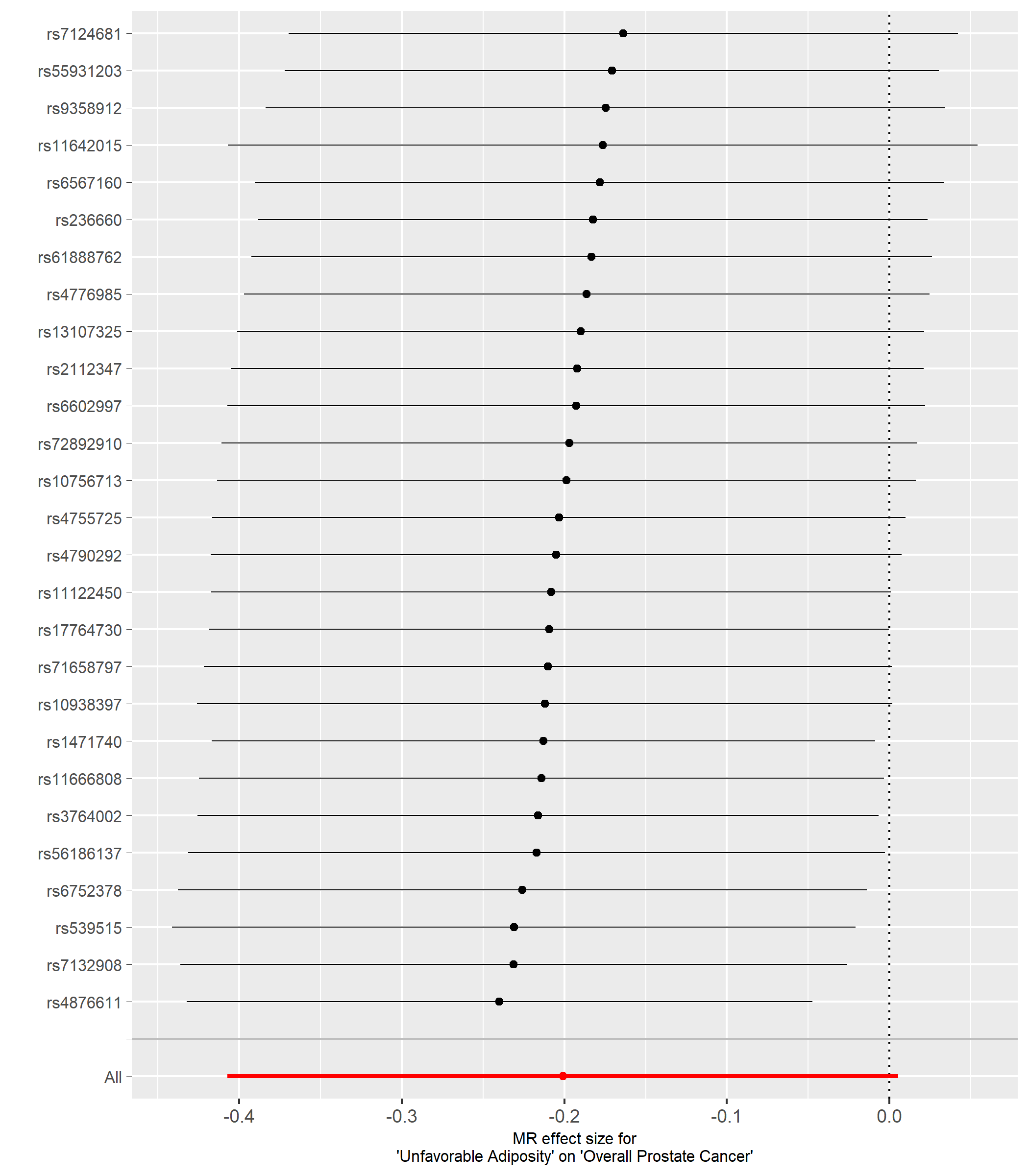


# Supplementary Figure 7: Leave-one-out analysis for MR examining the effect of unfavourable adiposity on overall prostate cancer.


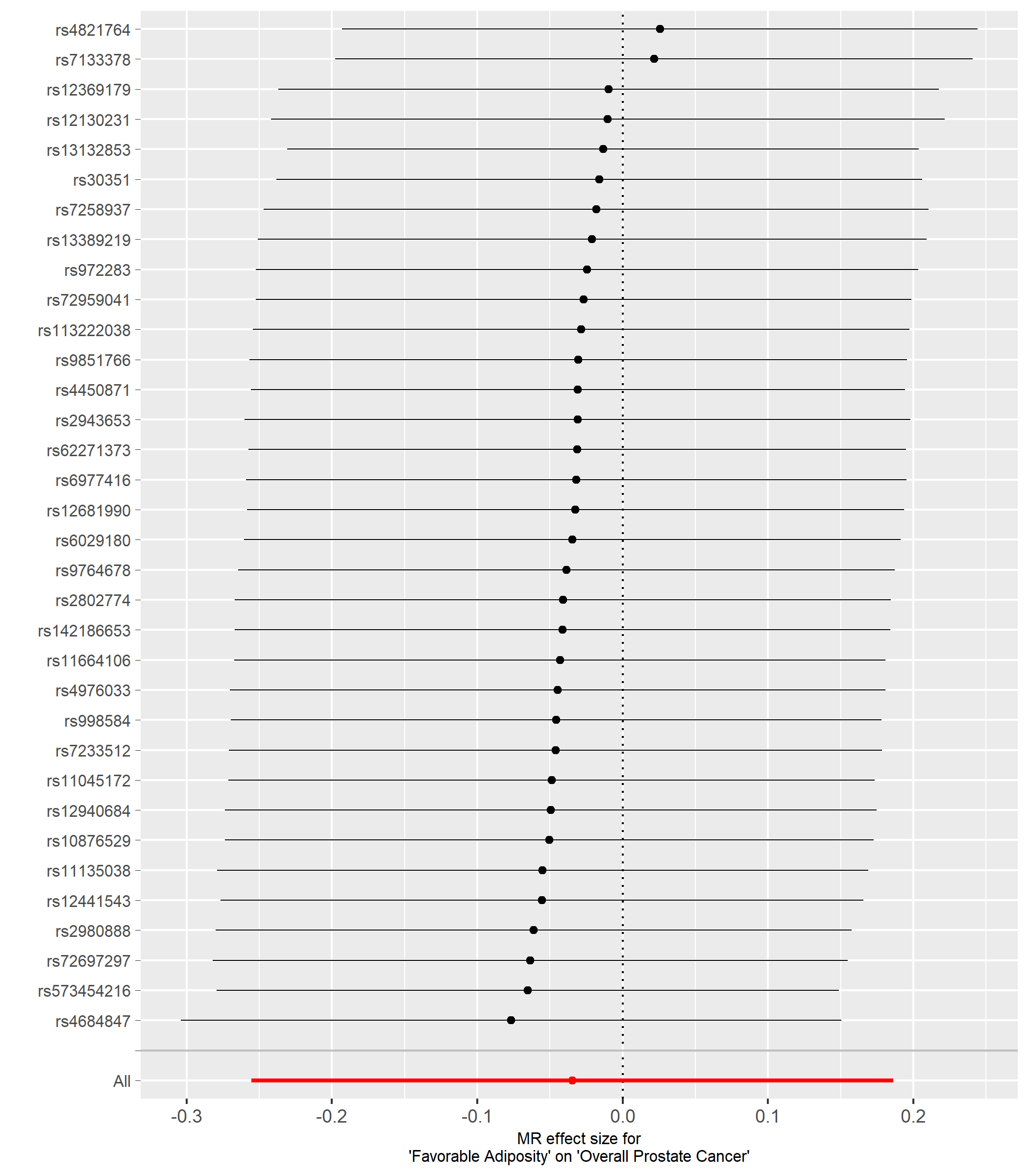


# Supplementary Figure 8: Leave-one-out analysis for MR examining the effect of favourable adiposity on overall prostate cancer.


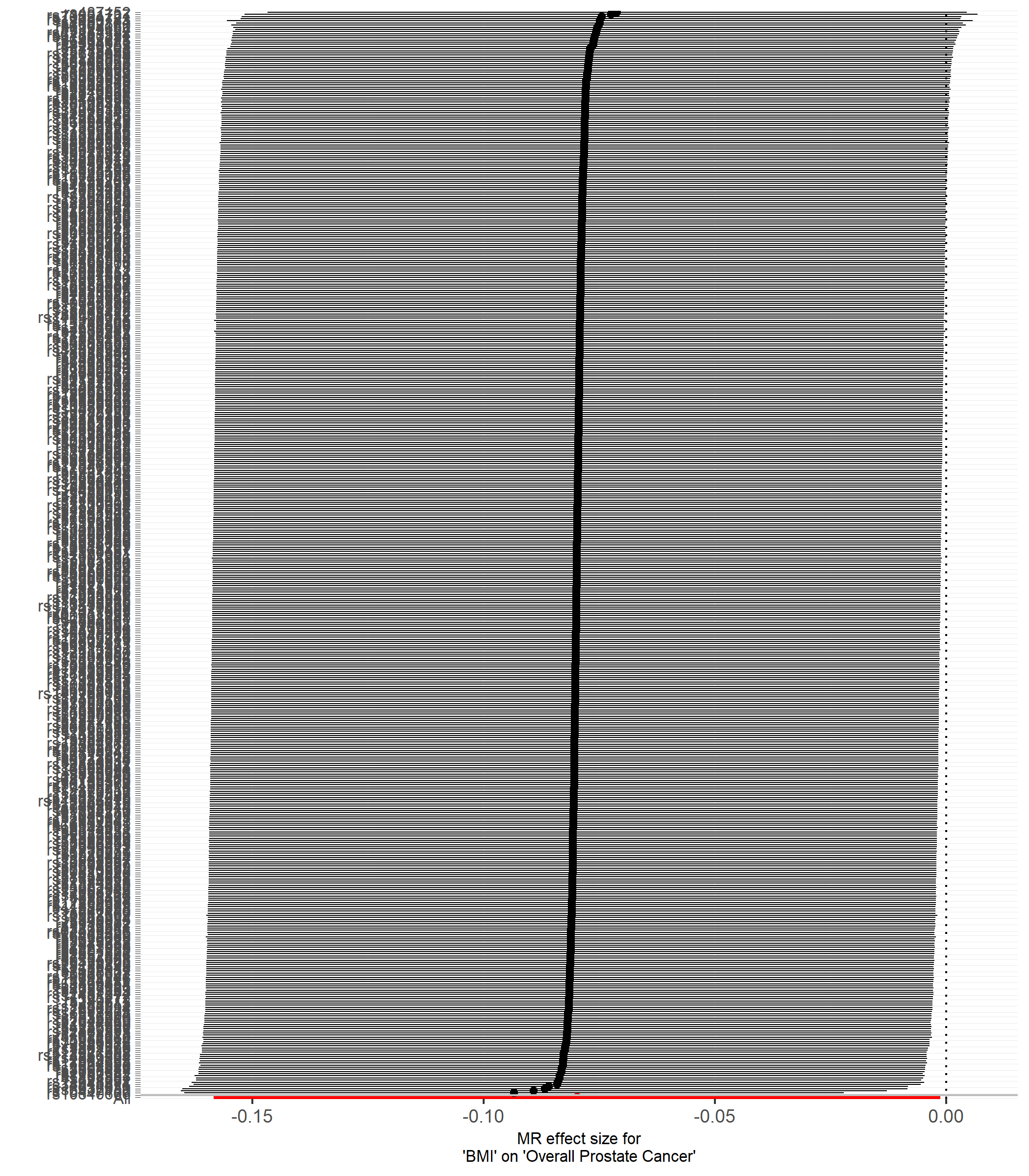


# Supplementary Figure 9: Leave-one-out analysis for MR examining the effect of BMI on overall prostate cancer.


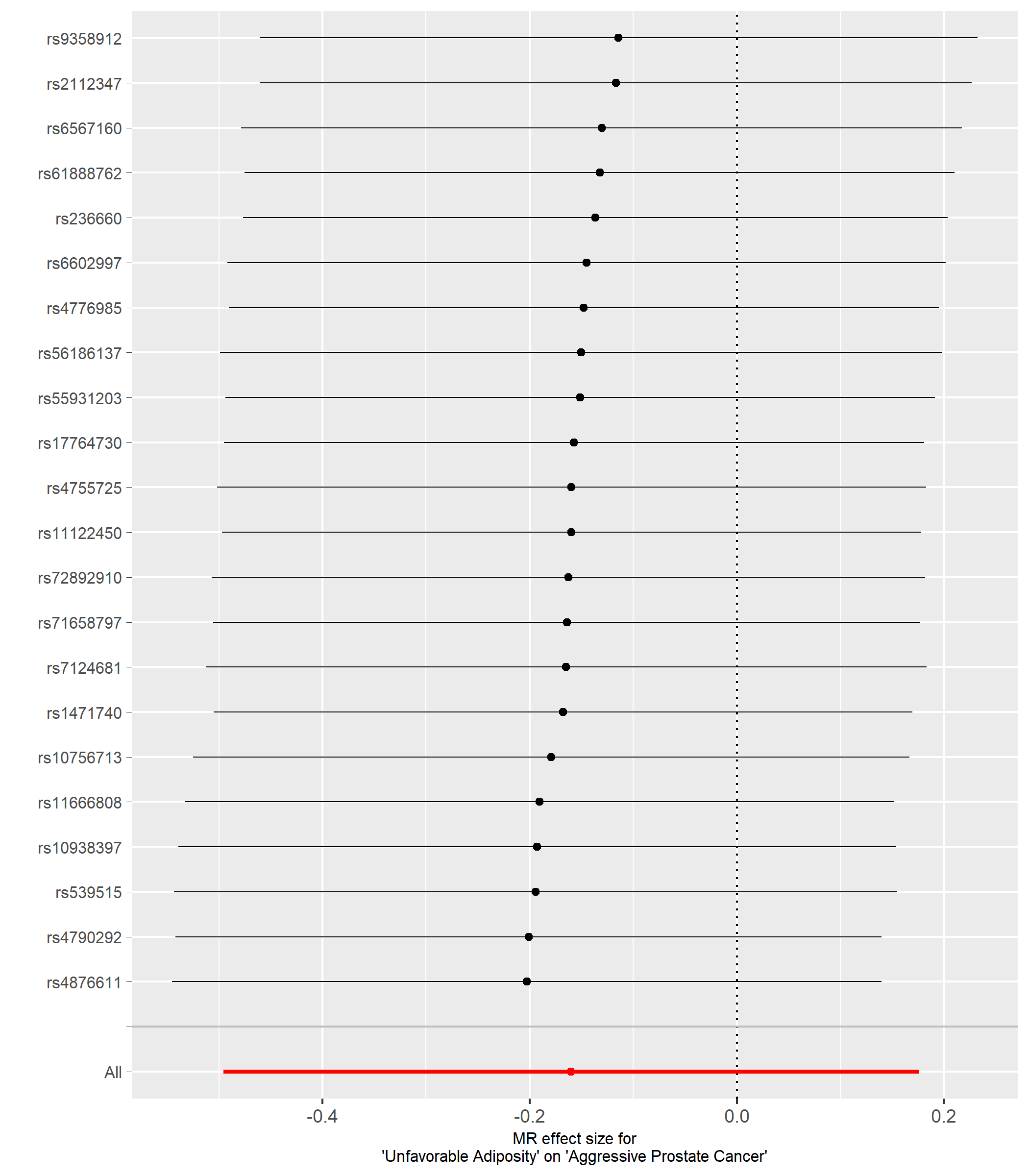


# Supplementary Figure 10: Leave-one-out analysis for MR examining the effect of unfavourable adiposity on aggressive prostate cancer.


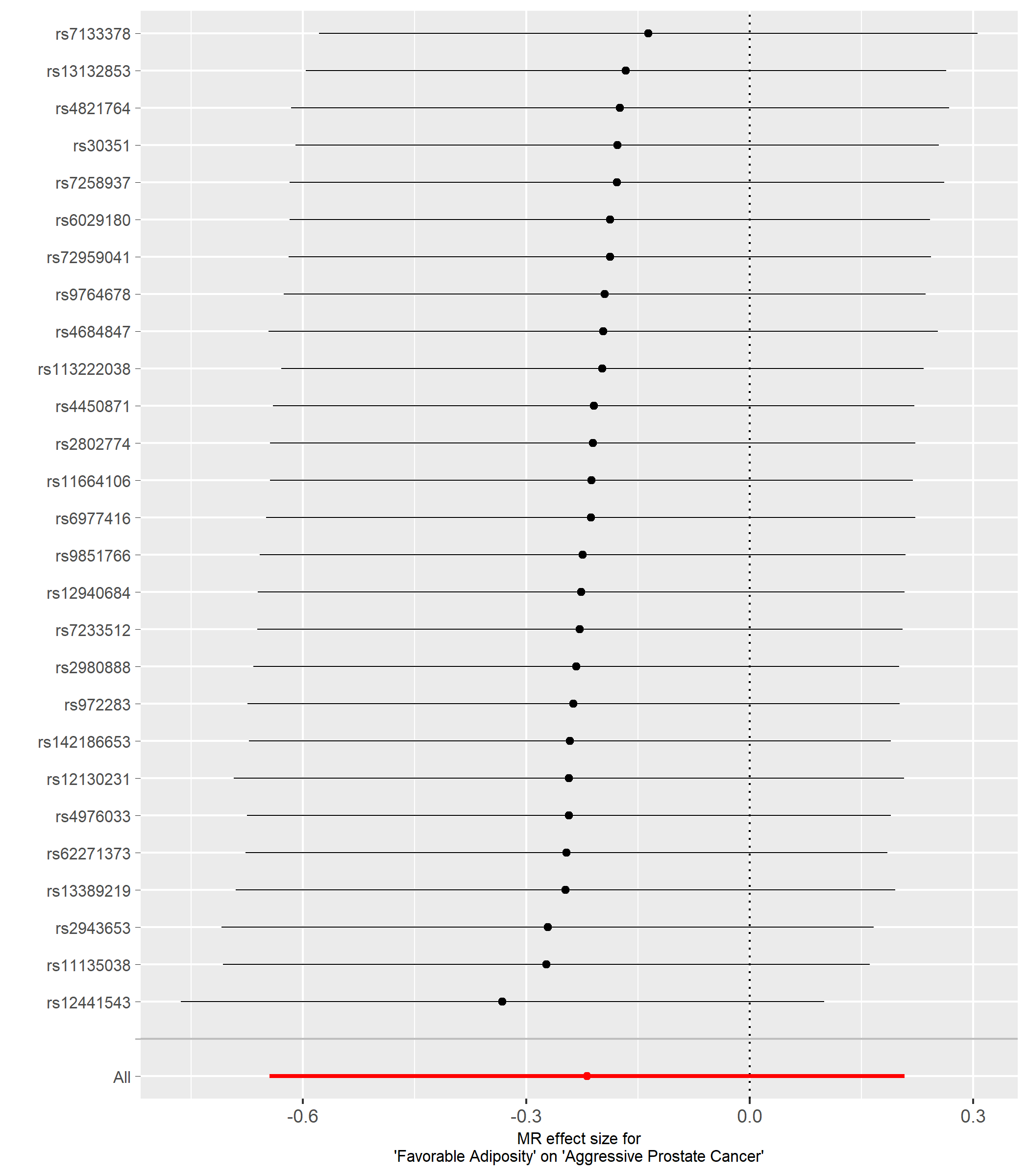


# Supplementary Figure 11: Leave-one-out analysis for MR examining the effect of favourable adiposity on aggressive prostate cancer.


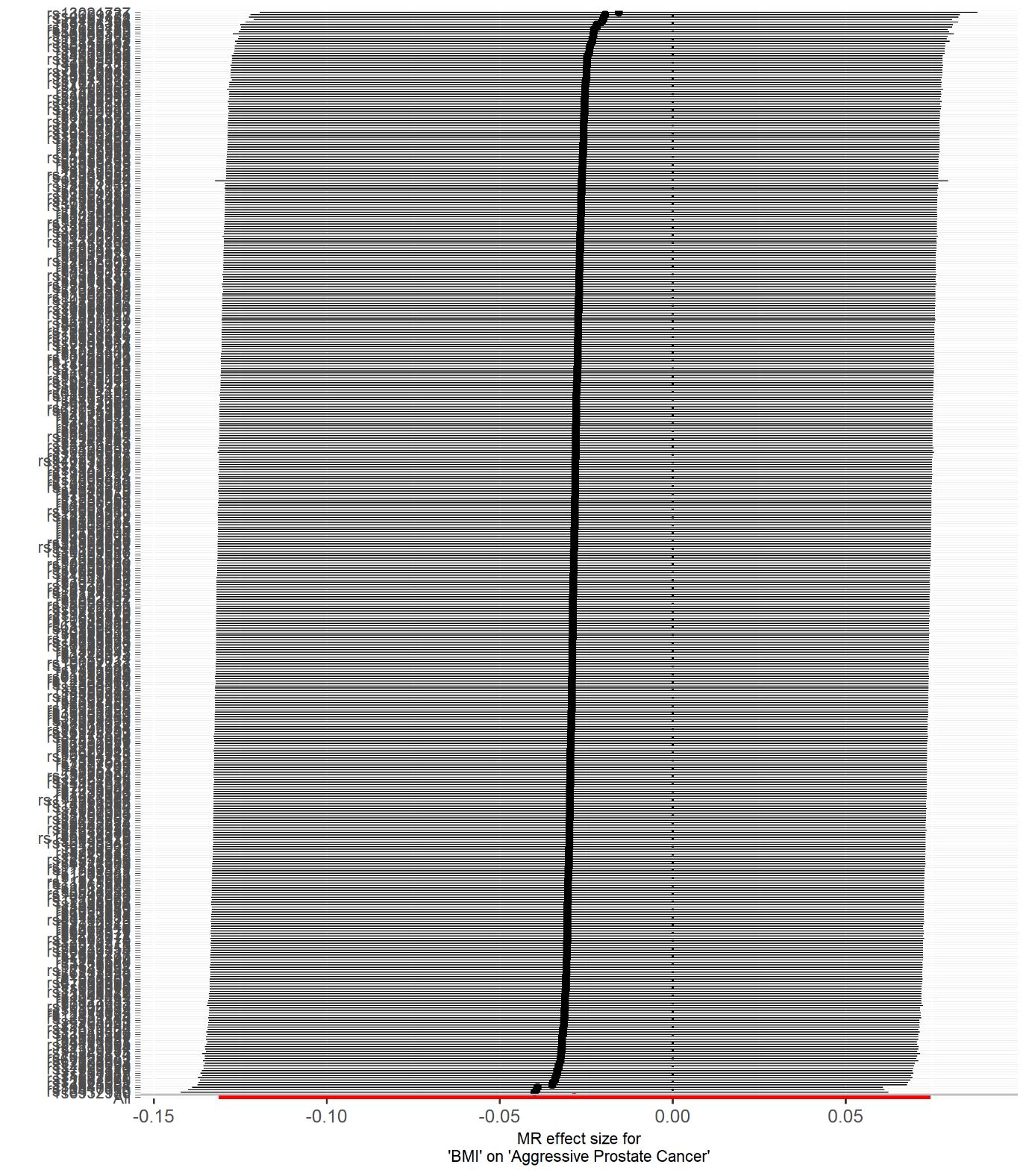


# Supplementary Figure 12: Leave-one-out analysis for MR examining the effect of BMI on aggressive prostate cancer.


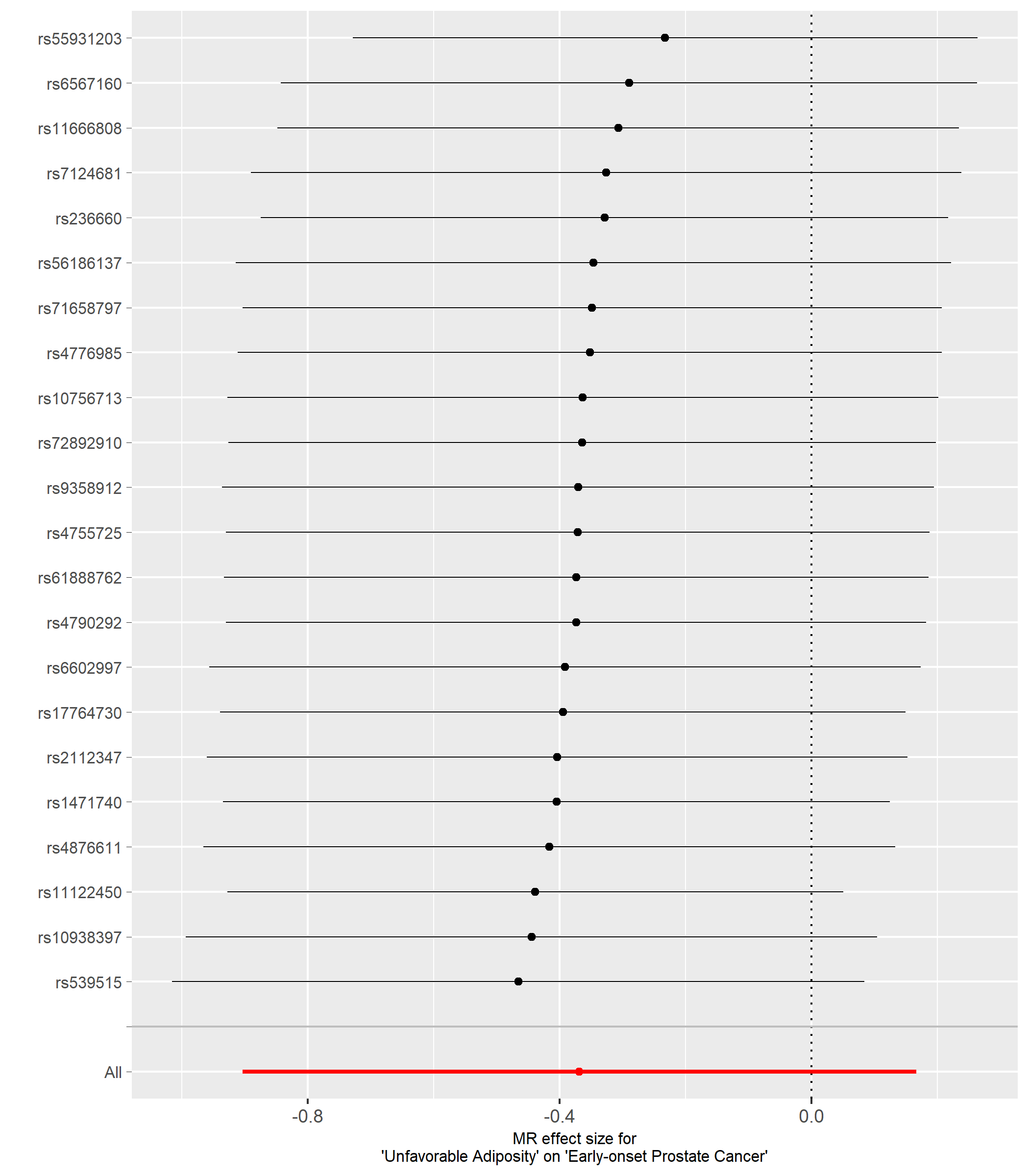


# Supplementary Figure 13: Leave-one-out analysis for MR examining the effect of unfavourable adiposity on early-onset prostate cancer.


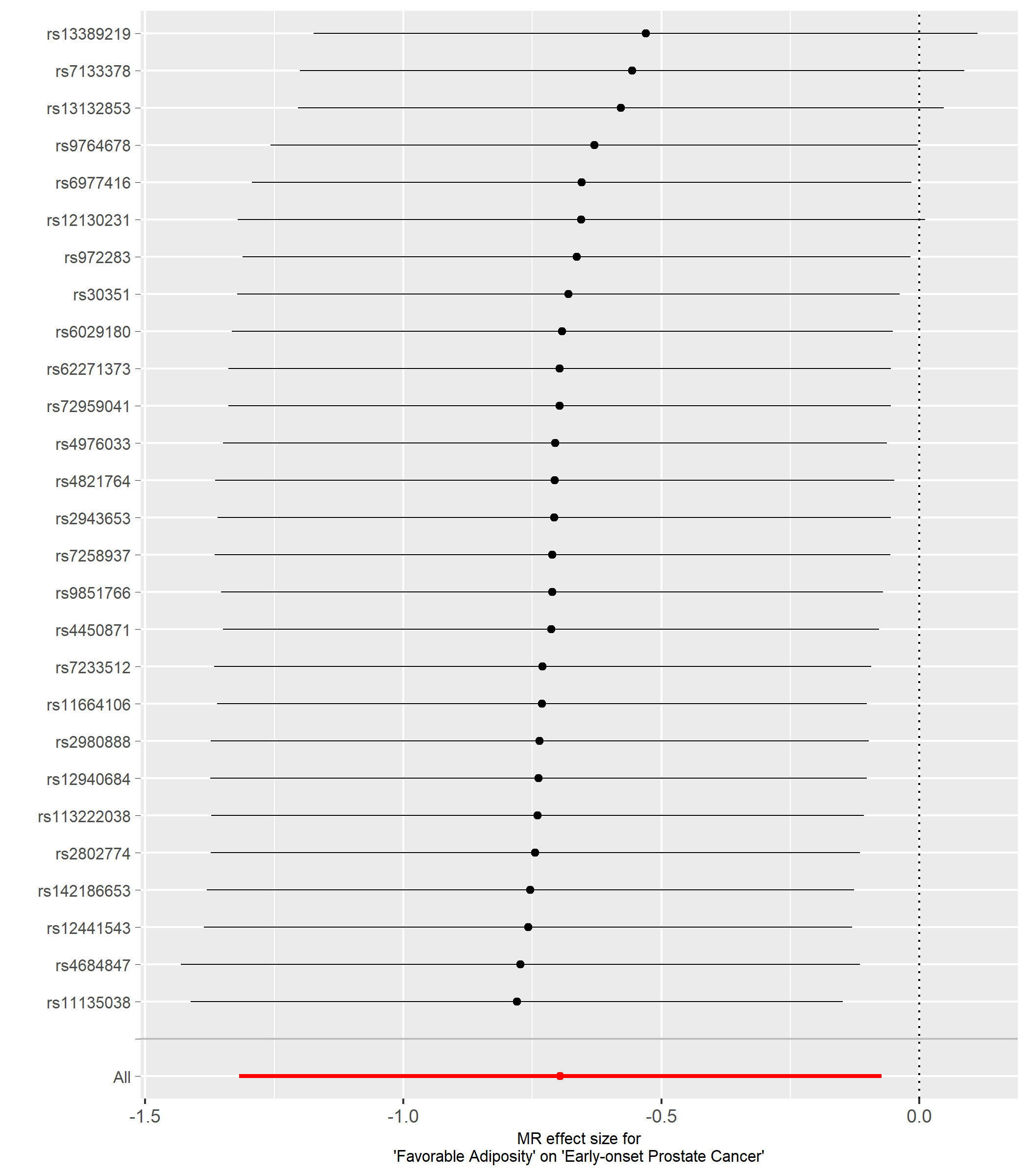


# Supplementary Figure 14: Leave-one-out analysis for MR examining the effect of favourable adiposity on early-onset prostate cancer.


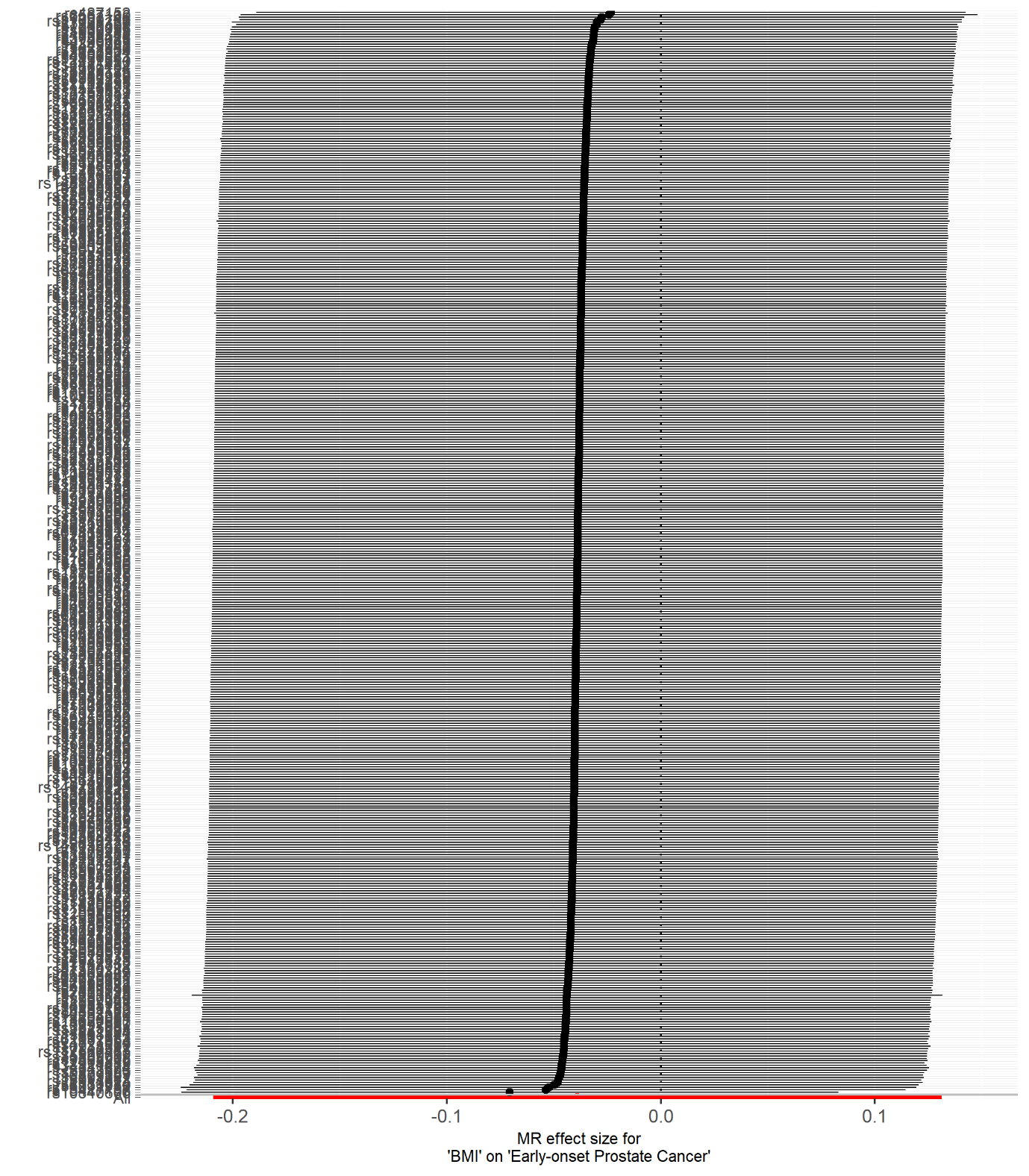


# Supplementary Figure 15: Leave-one-out analysis for MR examining the effect of BMI on early-onset prostate cancer.

# References

1. Kamat MA, Blackshaw JA, Young R, Surendran P, Burgess S, Danesh J, et al. PhenoScanner V2: an expanded tool for searching human genotype-phenotype associations. Bioinformatics. 2019;35(22):4851-3. doi: 10.1093/bioinformatics/btz469. PubMed PMID: 31233103; PubMed Central PMCID: PMCPMC6853652.

2. Staley JR, Blackshaw J, Kamat MA, Ellis S, Surendran P, Sun BB, et al. PhenoScanner: a database of human genotype-phenotype associations. Bioinformatics. 2016;32(20):3207-9. Epub 20160617. doi: 10.1093/bioinformatics/btw373. PubMed PMID: 27318201; PubMed Central PMCID: PMCPMC5048068.
